# Wnt-driven LARGE2 mediates laminin-adhesive O-glycosylation in human colonic epithelial cells and colorectal cancer

**DOI:** 10.1186/s12964-020-00561-6

**Published:** 2020-06-25

**Authors:** Vanessa Dietinger, Cira R. García de Durango, Svenja Wiechmann, Sophie L. Boos, Marlies Michl, Jens Neumann, Heiko Hermeking, Bernhard Kuster, Peter Jung

**Affiliations:** 1 German Cancer Research Center (DKFZ), Heidelberg, Germany; 2 German Cancer Consortium (DKTK), Partner site Munich, Munich, Germany; 3 DKTK Research Group, Oncogenic Signaling Pathways of Colorectal Cancer, Institute of Pathology, Ludwig-Maximilians-Universität München, Munich, Germany; 4 Chair of Proteomics and Bioanalytics, Technical University of Munich, Freising, Germany; 5 Institute of Pathology, Ludwig-Maximilians-Universität München, Munich, Germany; 6 Bavarian Center for Biomolecular Mass Spectrometry, Freising, Germany; 7 DKTK AG Oncogenic Signal Transduction Pathways in Colorectal/Pancreatic Cancer, Deutsches Krebsforschungszentrum (DKFZ) Heidelberg, DKTK Partnerstandort München, Institut für Pathologie der Ludwig-Maximilians-Universität (LMU) München, Thalkirchner Straße 36, D-80337 Munich, Germany

**Keywords:** O-glycosylation, LARGE2, Wnt signaling, Colorectal cancer, Organoid, Colonic stem cell

## Abstract

**Background:**

Wnt signaling drives epithelial self-renewal and disease progression in human colonic epithelium and colorectal cancer (CRC). Characterization of Wnt effector pathways is key for our understanding of these processes and for developing therapeutic strategies that aim to preserve tissue homeostasis. O-glycosylated cell surface proteins, such as α-dystroglycan (α-DG), mediate cellular adhesion to extracellular matrix components. We revealed a Wnt/LARGE2/α-DG signaling pathway which triggers this mode of colonic epithelial cell-to-matrix interaction in health and disease.

**Methods:**

Next generation sequencing upon shRNA-mediated silencing of adenomatous polyposis coli (APC), and quantitative chromatin immunoprecipitation (qChIP) combined with CRISPR/Cas9-mediated transcription factor binding site targeting characterized *LARGE2* as a Wnt target gene. Quantitative mass spectrometry analysis on size-fractionated, glycoprotein-enriched samples revealed functional O-glycosylation of α-DG by LARGE2 in CRC. The biology of Wnt/LARGE2/α-DG signaling was assessed by affinity-based glycoprotein enrichment, laminin overlay, CRC-to-endothelial cell adhesion, and transwell migration assays. Experiments on primary tissue, human colonic (tumor) organoids, and bioinformatic analysis of CRC cohort data confirmed the biological relevance of our findings.

**Results:**

Next generation sequencing identified the LARGE2 O-glycosyltransferase encoding gene as differentially expressed upon Wnt activation in CRC. Silencing of APC, conditional expression of oncogenic β-catenin and endogenous β-catenin-sequestration affected *LARGE2* expression. The first intron of *LARGE2* contained a CTTTGATC motif essential for Wnt-driven *LARGE2* expression, showed occupation by the Wnt transcription factor TCF7L2, and Wnt activation triggered LARGE2-dependent α-DG O-glycosylation and laminin-adhesion in CRC cells. Colonic crypts and organoids expressed *LARGE2* mainly in stem cell-enriched subpopulations. In human adenoma organoids, activity of the LARGE2/α-DG axis was Wnt-dose dependent. *LARGE2* expression was elevated in CRC and correlated with the Wnt-driven molecular subtype and intestinal stem cell features. O-glycosylated α-DG represented a Wnt/LARGE2-dependent feature in CRC cell lines and patient-derived tumor organoids. Modulation of LARGE2/α-DG signaling affected CRC cell migration through laminin-coated membranes and adhesion to endothelial cells.

**Conclusions:**

We conclude that the LARGE2 O-glycosyltransferase-encoding gene represents a direct target of canonical Wnt signaling and mediates functional O-glycosylation of α-dystroglycan (α-DG) in human colonic stem/progenitor cells and Wnt-driven CRC. Our work implies that aberrant Wnt activation augments CRC cell-matrix adhesion by increasing LARGE/α-DG-mediated laminin-adhesiveness.

Video abstract.

**Graphical abstract:**

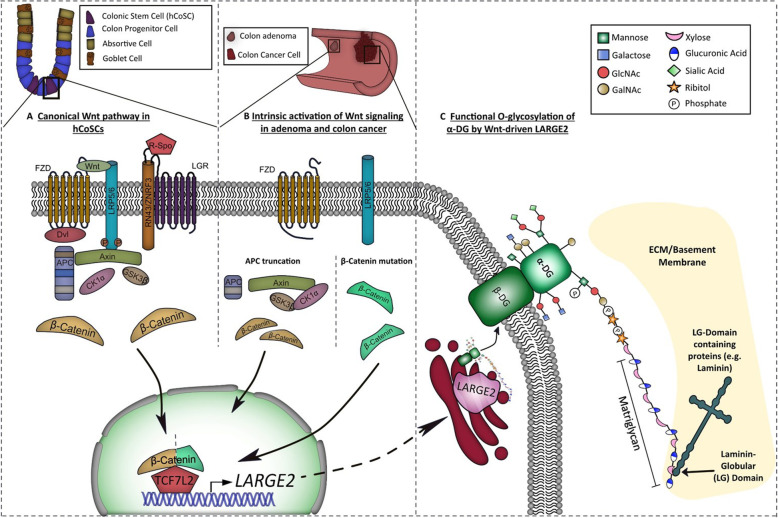

## Background

Wnt signaling plays a pivotal role in human colonic stem cell (hCoSC) and colorectal cancer (CRC) biology [1, 2], and constitutive Wnt activation in CRC frequently arises from mutations in the tumor suppressor gene *APC*. Wnt activity in *APC* mutant CRC cells at least partially depends on the length of truncated APC [3]. The majority of tumors harbor *APC* alleles altered in the mutation cluster region (MCR), and the encoded variants of truncated APC retain one or several 20 amino acid repeat (20*AAR) β-catenin binding sites, thus preventing full Wnt activation [4–6]. During CRC progression, Wnt signaling is frequently augmented by crosstalk with other corrupted signaling pathways [7, 8] or by extrinsic cues from the tumor microenvironment (TME) [9]. A concise characterization of effectors driven by activated Wnt signaling in a dose-dependent manner at different disease stages will help our understanding of CRC progression.

Upon Wnt activation, nuclear translocation of β-catenin and its association with TCF/LEF transcription factors leads to transcriptional regulation of target genes [10]. In the intestinal tract and in CRC, the β-catenin/TCF7L2 complex and its downstream target genes mediate epithelial tissue self-renewal [1, 11]. Importantly, β-catenin was initially found to control cell adhesion at adherens junctions [12]. APC itself interacts with cytoskeletal components, and its genetic alteration affects cell adhesion and migration [13]. Besides this, Wnt signaling drives expression of extracellular matrix (ECM) proteins, such as fibronectin [14] and laminin [15]. In return, differential interactions of intestinal stem cells (ISCs) and CRC cells with the extracellular matrix (ECM) contribute to acquisition of epithelial stemness and metastatic tumor traits, respectively [16].

The outer membrane protein α-dystroglycan (α-DG) and the transmembrane protein β-dystroglycan (β-DG) are proteolytic cleavage products of the same pro-peptide DAG1, and O-glycosylated α-DG functions as a receptor for laminin-domain containing ECM protein ligands, such as laminin, agrin, and neurexin [17, 18]. At least 23 gene products are involved in the process of O-mannosyl-glycan synthesis on α-DG, and their loss of function contributes to the pathology of congenital muscular dystrophies [18]. Once a core glycosyl structure on α-DG has been generated by priming enzymes*,* the glycosyltransferases LARGE1 or its paralog LARGE2 (a.k.a. GYLTL1B) polymerize a glucosaminoglycan disaccharide on phosphorylated O-linked mannose, often referred to as matriglycan, which is essential for α-DG binding to laminin [18–20]. While LARGE1 is essential for α-DG O-glycosylation in heart, brain, and skeletal muscle, a functional necessity for LARGE2 regarding this process exists in kidney and prostate [21, 22]. However, the biology of LARGE2 in the human colon and in CRC is largely unknown.

Here, we report a direct link between canonical Wnt signaling and the LARGE2-dependent, O-glycosylation-mediated laminin adhesion in human colonic epithelium and in CRC. By using the human colon (tumor) organoid model [2, 23] and CRISPR/Cas9-mediated genome editing, we addressed the consequences of physiologic extrinsic and oncogenic intrinsic Wnt activation for this process.

## Methods

### Cell culture

Cell lines KM12c and KM12-L4a were a kind gift from Professor I. Fidler (MD Anderson, TX, USA). HT-29, SW620, LS174T, Colo205, KM12c/L4a, and HEK293T (ATCC, LGC Standards, Wesel, Germany) were maintained in Dulbecco’s Modified Eagle Medium supplemented with 10% FBS (both Gibco, Thermo Fisher Scientific, MA, USA) and 1% penicillin/streptomycin (Gibco). SW480 cells were cultivated in RPMI-1640 medium (Gibco), supplemented with 10% FBS and 1% penicillin/streptomycin (Gibco). RKO cells were maintained in McCoy’s 5A (Sigma, Merck, Darmstadt, Germany) supplemented with 10% FBS and 1% penicillin/streptomycin (Gibco). HMEC-I cells were kindly provided by PD Dr. Thomas Grünewald (DKFZ Heidelberg) and maintained in RPMI-1640 medium supplemented with 50 ng/ml human epidermal growth factor (EGF, PeproTech, NJ, USA) and 1 μg/mL hydrocortison (Sigma, Merck). Cultures were kept at 37 °C, 5% CO_2_ and subcultured as needed. All cell lines were regularly tested negative for Mycoplasma contamination by using the LookOut Mycoplasma PCR detection kit (Sigma, Merck).

### Next generation sequencing (RNA-Seq) analysis and bioinformatic data processing

For RNA-Seq, total RNA was isolated from HT-29 cells using the PureLink RNA purification kit and PureLink DNase (both Invitrogen, Thermo Fisher) according to the manufacturer’s protocol. Quality of isolated RNA was confirmed with the Agilent BioAnalyzer 2100 (Agilent, CA, USA). Sequencing libraries were prepared using the TrueSeq Stranded mRNA Library Prep Kit for Illumina (New England Biolabs, MA, USA) according to the manufacturer’s instructions. 50 bp single-read sequencing was performed on a HiSeq 2000 v4 (Illumina, CA, USA) according to the manufacturer’s protocol. For the bioinformatic data analyses, base calling was done with bcl2fastq 2.19.0.316. For all samples, low quality bases were removed with Fastq_quality_filter from the FASTX Toolkit 0.0.13 (http://hannonlab.cshl.edu/fastx_toolkit/index.html) with 90% of the read needing a quality phred score > 20. Homertools 4.7 [24] were used for PolyA-tail trimming, and reads with a length < 17 were removed. Genomic mapping was performed with TOPHAT2 [25] for filtered reads with human genome assembly 38 and PicardTools 1.78 CollectRNASeqMetrics (https://broadinstitute.githu.io/picard/). Count data were generated by htseq-count [26] using the gencode.v29.annotation.gtf (https://www.gencodegenes.org/) file for annotation. For the comparison with DESeq2 [27], the input tables containing the replicates for the groups to compare were created by a custom perl script. In the count matrix, rows with an average count number < 10 were removed. Then, DESeq2 (version 1.4.1) was run with default parameters. The results tables were annotated with gene information (gene symbol, gene type) derived from the gencode.v29.annotation.gtf file.

### Gene expression data and gene set enrichment analysis (GSEA)

Gene expression data of COAD, READ and PRAD were obtained from GDC-TCGA datasets [28], and another set covering 566 CRC cases was derived from Marisa et al. [29] (GSE35982). Data of 515 transplanted human tumor samples (PDX) from 244 patients were included from the Isella et al. (GSE76402) data set [30]. Information to assign the samples with the CMS subtypes are described in Guinney et al. [31] and the CRIS categories are described in Isella et al. [30]

*LARGE2* gene signatures were generated from GDC-TCGA datasets as well as from the PDX microarray data described by Isella et al. GSEA was performed as described in Subramanian et al. [32] using the GSEApreRanked tool of the GSEA 3.0 Desktop Application (Broad Institute, http://www.gsea-msigdb.org/gsea/index.jsp).

### Chromatin Immunoprecipitation and analysis

SW480 cells were used for ChIP analysis according to manufacturer’s protocol (SimpleChIP® Enzymatic Chromatin IP Kit #9003, Cell signaling Technology). 10 μg of fragmented DNA were incubated over night with either TCF7L2 (1:50, C48H11) or control rabbit IgG (1:500, #2729) antibody (both Cell Signaling Technology). The precipitated chromatin fragments were purified, and analyzed via qRT-PCR using primer listed in Additional file 13.

### CRISPR/Cas9-mediated engineering of APC mutant human adenoma organoids

Isolation of crypts from patient material and 3D cultivation as patient-derived organoids (PDOs) was performed as previously described [2], and colonic PDOs were transfected by lipofection as has been reported [33]. In brief, PDOs grown in WREN medium (Wnt3a, RSPO-3 and Noggin conditioned media derived from L-WNR cells, CRL-3276, ATCC) and Advanced DMEM/F12 (ADF, Gibco) 50:50, supplemented with Glutamax, 10 mM HEPES, N-2 [1×], B-27 without retinoic acid [1×] (Invitrogen, Thermo Fisher), 1 mM N-acetylcysteine (Sigma, Merck), 50 ng/ml recombinant human epidermal growth factor (PeproTech), 7.5 μM SB202190 (Sigma, Merck) and 10 μM Y27632 (SelleckChem, TX, USA) were disaggregated with TripLE Select (Gibco, Thermo Fisher) for 10 min at 37 °C. Single PDO cells were resuspended in WREN medium without antibiotics, supplemented with Rho kinase inhibitor Y-27632 (10 μM) and plated in 24-well low attachment plates (160,000 cells per well) already containing 200 μl of lipocomplexes. Ribonucleoparticles consisting of Cas9 and single guide (sg) RNA were prepared according to manufacturer specifications (IDT, IA, USA), and the cell/enzyme mixture was centrifuged for one hour at 32 °C, followed by 4 h of incubation at 37 °C / 5% CO_2_. Cells were then plated in Matrigel (BD Corning, NY, USA) and kept in WREN medium for three days prior to selection. Selection for ADOs was achieved by omitting Wnt and RSPO from the culture medium (EN medium).

### Patient-derived tumor organoid (PDTO) isolation and culture

Tissue pieces from primary CRC or liver metastasis were cut into small pieces and one fraction was processed for subsequent immunohistochemistry analysis. The remaining tissue was incubated with Normocin (Invivogen) and Antibiotic-Antimycotic (Thermo Fisher) for 15 min at room temperature. Next, pieces were resuspended in disaggregation media (ADF (Thermo Fisher), 5 U/ml Dispase (Stem Cell Technologies, Vancouver, Canada), 75 U/ml Collagenase IV (Gibco, Thermo Fisher) and 10 μM Y-27632 (SelleckChem) and incubated at 37 °C for 30 min before passing through a 1.2 mm needle. Once small cell clusters were obtained, the suspension was passed through 70 μm cell strainer (BD, NJ, USA) and freed from erythrocytes using ammonium-choride buffer. CRC cells were resuspended in Matrigel, plated in 24-well tissue culture plates and overlaid with tumor organoid culture (TOC) medium (ADF supplemented with 10 mM HEPES, Glutamax, 1x B27 (all Thermo Fisher), 1 mM N-acetylcysteine (Sigma, Merck), 50 ng/mL recombinant human epidermal growth factor (EGF, PeproTech), 0.015 μM Prostaglandine E2 (PGE2, Sigma, Merck), 25 ng/mL human Noggin (PeproTech), 7.5 μM SB202190 and 0.5 μM LY2157299 (Selleckchem), and 50 μg/mL Normocin (Invitrogen). 10 μM Y27632 (SelleckChem) was added for 48 h to avoid anoikis. Medium was replaced every 2–3 days. For serial passaging, PDTOs were disaggregated using 0.025% Trypsin (Gibco, Thermo Fisher) and subsequently passed through a 0.8 mm needle. After washing with ADF medium, PDTO cells and small cell aggregates were resuspended in Matrigel and solidified drops were overlaid with TOC medium.

### Immuno-labeling of human CRC cells and PDOs for flow cytometry-assisted cell sorting (FACS)

For surface abundance analysis of O-glycosylated α-DG, cells were detached with 8 mM EDTA/PBS, washed and resuspended in staining buffer (PBS/5% FBS). Cells were stained with IIh6c4 antibody (1:100, Millipore/Merck) for one hour on ice, followed by labelling with a FITC-coupled secondary antibody (1:200, Jackson ImmunoResearch, UK, Cat.No: 515–095-062). Control cells were stained with the secondary antibody alone. DAPI (0.3 μg/ml, Carl Roth, Karlsruhe, Germany) was added to exclude dead cells. Analysis was performed on a LSR Fortessa (BD) instrument, and data analysis was done with FlowJo software (BD).

Labeling and sorting of PDO cells was performed as described previously [34]. In brief, human colonic organoids were disaggregated using Dispase II solution (Sigma, Merck), and cells resuspended in FACS buffer (ADF, 10 mM HEPES, 10 mM Glutamax, 5% FBS, 10 μM Y-27632). Cells were stained with Allophycocyanin (APC)-coupled anti-PTK7 antibodies (1:20, Cat.No: CCK-4, Miltenyi, Bergisch Gladbach, Germany,). Viable, DAPI-negative cell fractions were used to determine auto-fluorescence and to specify the “negative”, non-staining cell population for FACS gating. The brightest 14–15% PTK7+ cells were sorted as the PTK7-high fraction. The PTK7-low population comprised the 25–30% PTK7+ cells adjacent to the PTK7-high population, while the PTK7-negative subpopulation did not stain for PTK7. Cell sorting was performed on a FACS Aria III instrument (BD) and analysis was performed with FlowJo software. After cell sorting, the different fractions were pelleted by centrifugation and re-suspended in 500 μl Trizol (Invitrogen, Thermo Fisher) for RNA isolation and gene expression analyses via TaqMan™ gene expression assays (Applied Biosystems, Thermo Fisher, see Additional File 13 for probe IDs).

Sub-fractionation of human colonic crypt cells was performed essentially as described previously [2] by immuno-labelling of epithelial crypt single cells with anti-EPHB2 antibody (1:100, BD Biosciences, clone 2H9, APC-coupled). Viable control cells (DAPI-negative fraction) were used to define the EPHB2-negative cell population.

### WGA-AE purification, immunoblot analysis of glycoproteins and laminin overlay assay

Whole cell protein lysates were prepared from sub-confluent cultures using WGA Lysis Buffer (50 mM Tris/HCl, pH 7.4 (Sigma), 150 mM NaCl (Carl Roth), 1% Triton X-100 (AppliChem, Darmstadt, Germany) and Protease Inhibitors (Sigma, Merck), and protein concentration was determined with Bradford Reagent (Sigma, Merck). For precipitation of glycoproteins, 1–3 mg of lysate was incubated with 50 μl equilibrated WGA agarose beads (Vector labs, CA, USA) overnight at 4 °C while rotating. Glycoproteins were eluted from WGA beads by heating for 10 min at 70 °C in 60 μl of 2x Laemmli buffer. Whole cell or glycoprotein-enriched lysates were separated on 8–10% SDS-acrylamide gels and proteins transferred to Immobilon PVDF membranes (Millipore, Merck). For the laminin overlay assay, membranes were blocked in 5% skim milk in laminin binding buffer (LBB; 19 mM triethanolamine, 140 mM NaCl, 1 mM MgCl_2_, 1 mM CaCl_2_, pH 7.4) for one hour at room temperature. After washing in 3% BSA/LBB, membranes were incubated with 5 μg/mL Matrigel (contains 60% laminin-111) in 3% BSA/LBB at 4 °C overnight. Unbound residual Matrigel/laminin-111 was washed of the membrane carefully with 5% milk in LBB. For immunodetection, membranes were incubated with the following antibodies: anti-APC (1:500, Ali 12–28, SantaCruz Biotech), anti-actin (1:2000, A2066, Sigma, Merck), anti-tubulin (1:2000, T9026, Sigma, Merck), anti-αDG (1:500, IIh6c4, Millipore, Merck), anti-DAG1 (1:500, 11,017–1-AP, PTGLAB, Manchester, UK), anti-laminin (1:2000, NB300–144, Novus Bio, MN, USA). Chemiluminescence signals from horseradish-peroxidase (HRP) coupled secondary antibodies (1:10000, Jackson ImmunoResearch) were generated using Immobilon Western HRP Substrate (MerckMillipore) and detected with a Laser Scanning System (Odyssey Fc, LI-COR, NE, USA).

### LC-MS/MS analysis of WGA-IP enriched glycoproteins

Glycoprotein enriched lysates were prepared as described above. Proteins bound to WGA agarose beads were eluted with LDS sample buffer (NuPAGE, Thermo Fisher) containing 100 mM DTT for 10 min at 70° C and alkylated with 55 mM CAA for 30 min at RT. WGA eluates were purified and fractionated into six fractions by NuPAGE Bis-Tris Gels (Thermo Fisher) and subsequently subjected to in-gel tryptic digestion according to standard procedures. After drying in a centrifugal evaporator, the samples were stored at − 80 °C until LC-MS/MS analysis.

Nano-flow LC-MS/MS measurement of peptides in eluates was performed using a nanoLC UltiMate 3000 (Thermo Fisher) coupled to a quadrupole-Orbitrap Q Exactive HF mass spectrometer (Thermo Fisher). Peptides were desalted on a trap column (100 μm × 2 cm, packed in-house with Reprosil-Pur C18-AQ 5 μm resin; Dr. Maisch GmbH, Ammerbuch-Entringen, Germany) in 0.1% FA at 5 μl/min and separated on an analytical column (75 μm × 40 cm, packed in-house with Reprosil-Pur C18-AQ, 3 μm resin; Dr. Maisch) using a 22 min linear gradient from 4 to 32% solvent B (0.1% formic acid, 5% DMSO in acetonitrile) in solvent A (0. 1% formic acid, 5% DMSO in water) at a flow rate of 300 nL/min. The mass spectrometer was operated in data dependent acquisition and positive ionization mode. MS1 spectra were acquired over a range of 360–1300 m/z at a resolution of 60,000 in the Orbitrap by applying an AGC of 3e6 or maximum injection time of 10 ms. Up to 10 peptide precursors were selected for fragmentation by higher energy collision-induced dissociation (HCD; 1.7 m/z isolation window, AGC value of 1e5, maximum injection time of 25 ms) using 25% normalized collision energy (NCE) and analyzed at a resolution of 15,000 in the Orbitrap (Thermo Fisher).

### Transwell migration assay

To analyze invasion, cell inserts (0.8 μm pore size), were coated with 1 μg/mL Laminin 111 from Engelbreth-Holm-Swarm (EHS) lathrytic mouse tumor (Santa Cruz Biotechnology, TX, USA) for 1 h at room temperature. 1× 10^5^ cells, deprived of serum for at least 24 h, were seeded in serum free DMEM on laminin in the upper chamber. As chemo-attractant, DMEM, supplemented with 10% FBS, was placed in the bottom chamber. After 24–48 h, cells on the upper side of the membrane were removed using a cotton swab. After fixation in 4% PFA for 20 min, the membrane was air dried and stained with 100 ng/mL DAPI in 0.1% Triton X-100. Images were captured with a Zeiss LSM 700 confocal microscope in five random fields per membrane and migrated cells counted with ImageJ. Experiments were performed three times in duplicates. Relative migration was normalized to the corresponding control.

### CRC cell adhesion assay on HMEC-1 cells

2× 10^5^ HMEC-1 cells per 48-well were seeded to form a confluent endothelial monolayer within 48–72 h. Separately cultured, sub-confluent KM12-L4a-Luc CRC cells (stably transduced with pV2-Luc2, kindly provided by Prof. Andreas Trump, DKFZ Heidelberg, and wt or KO for LARGE2) were detached carefully with Accutase, adjusted to 0,75 * 10^6^ single cells /mL, and 200 μl of this suspension was added to confluent HMEC-I cells for the indicated time. Next, supernatants were removed and short-term co-cultures were washed twice with PBS. Cells were lysed in 50 μl 1x Passive Lysis Buffer (Promega, WI, USA), lysates were spun down and 10 μl was used for measurement on a Berthold Orion II Microplate Luminometer after adding 50 μl Beetle Juice (pjk Biotech, Kleinblittersdorf, Germany). Three experiments were performed independently on different days (biological triplicates) and each sample was measured three times (technical triplicates).

### Immunohistochemistry

Immunohistochemical (IHC) staining was carried out on 2 μm formalin-fixed, paraffin-embedded sections using standard procedures. In brief, antigen retrieval was achieved with target retrieval solution (S1699, Agilent Technologies) via microwave heating. Incubation with the primary antibody IIh6 (Santa Cruz) at a concentration of 4 μg/ml was done at room temperature for one hour. As a detection system, biotinylated anti-mouse IgM (BA2020, Vector Labs) and streptavidin-HRP (RE 7104, Novocastra, Newcastle, UK) was used. Samples were developed via exposure to 3,3`-diaminobenzidine (DAB+, K3468, Agilent) and counterstained with hematoxylin Gill’s Formula (H-3401, Vector Labs). For IHC staining against α-DG on mouse FFPE tissue, Crystal MausBlock (Fa. DCS, Hamburg, Germany, ML125R015) was used to avoid non-specific binding of the secondary antibody. Processed slides were scanned on a Vectra Polaris™ slide scanner using 40-fold scan resolution and snapshots taken via Phenochart 1.0.8 software (both AKOYA Biosciences, MA, USA).

### In situ hybridization

Detection of *Large2* mRNA in mouse ileum normal mucosa and ApcMin-driven adenoma was performed as described before [35]. In brief, RNA probes were created with DIG RNA Labeling Kit (Roche) according to the manufacturers’ protocol. 5 μm thick FFPE sections of mouse tissues were de-waxed and re-hydrated using standard procedures. Proteins were digested by treating the slides with 0.2 N HCl, followed by incubation with 30 μg/mL Proteinase K in PBS at 37 °C. Digestion was stopped with 0.2% Glycine/PBS. Sections were post-fixed in 4% PFA for 10 min, washed in PBS three times and histones acetylated with acetic anhydride solution (1.5% triethanolamine, 0.15% HCl, 0.6% acetic anhydride). Slides were washed and incubated for one hour at 65 °C in pre-hybridization solution, containing 50% Formamide, 5X SSC pH 4.5, 2% Blocking Reagent (Roche), 0.05% CHAPS, 5 mM EDTA, 50 μg/ml Heparin and 50 μg/ml yeast RNA (all Sigma, Merck). Probes were used in a concentration of 500 ng per labeling, diluted in pre-hybridization solution and incubated for 24 h at 65 °C. Post-hybridization washes were performed using 50% Formamide in 2x SSC Buffer pH 4.5, three times á 20 min at 65 °C. Sections were then rinsed in Tris/NaCl buffer and blocked for 30 min in blocking solution (0.5% blocking powder in Tris/NaCl). Sheep anti-DIG antibody (Roche, Basel, Switzerland) was diluted 1:2000 in blocking solution and incubated overnight at 4 °C. Finally, samples were first washed in Tris/NaCl buffer and then in NTM buffer (0.1 M Tris pH 9.5, 0.1 M NaCl, 0.05 M MgCl_2_) before developing the sections in BCIP/NBT Liquid Substrate (Roche) for 48 h. Processed slides were scanned on a Vectra Polaris^TM^ slide scanner using 40-fold scan resolution and snapshots taken via Phenochart 1.0.8 software (both AKOYA Biosciences).

### Statistical analysis

GraphPad Prism software (v7.01) was used for statistical analyses. For calculation of significant differences between two groups of biological replicates, a Student’s t-test (unpaired, two-tailed, 5% FDR) was applied. For qRT-PCR data, standard deviation (SD, *n* ≥ 3) was calculated as indicated. For the comparison of 4–5 patient cohort subpopulations (CMS and CRIS classification), a multiple comparison one-way ANOVA test was applied. For calculation of correlation coefficients, Pearson’s correlation analysis was applied.

## Results

### Silencing of truncated APC activates Wnt target gene expression in CRC cells

**Fig. 1:**
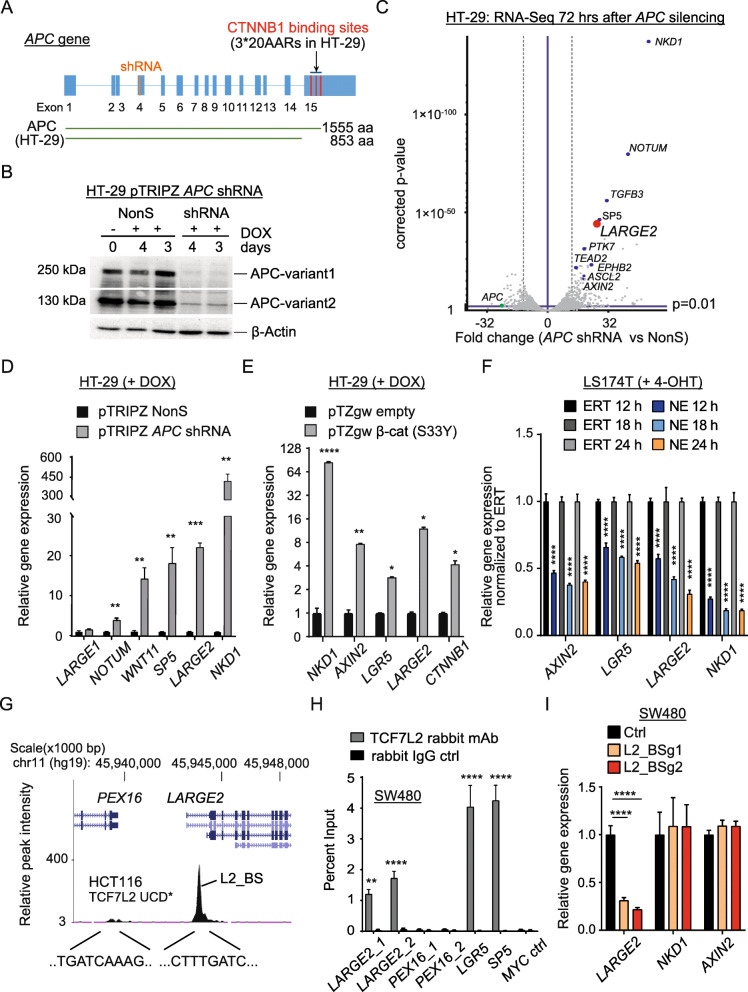
*LARGE2* is a direct target of Wnt signaling in colorectal cancer. **A)***APC* gene structure, exons indicated as blue bars. A short hairpin RNA (shRNA, indicated in orange) was used to silence *APC*. Truncated APC variants in HT-29 CRC cells are shown as green lines. aa: amino acids. **B)** Immunoblot analysis of APC and β-actin on WCL from HT-29 cells treated with 500 ng/ml DOX to induce expression of *APC*-targeting shRNA or a non-silencing shRNA (NonS). **C)** Volcano plot showing genes deregulated 72 h after silencing of *APC* in HT-29 cells, as analyzed via RNA-Seq on biological duplicates. Cut off: normalized *p*-value < 0.01 (horizontal blue line), fold change > 4 (vertical dotted lines). **D**,**E**) qRT-PCR analysis of indicated genes up-regulated upon conditional APC silencing **(D)** or expression of S33Y-mutated CTNNB1 **(E)** in HT-29 cells for 72 h (+ 500 ng/ml DOX). Results are shown as mean ± SD (*n* = 3); * *p* < 0.05; ** *p* < 0.01; **** *p* < 0.0001. **F)** qRT-PCR analysis of indicated genes in LS174T-NE or -E cells after treatment with 400 nM 4-OHT for the indicated times. Results are shown as mean ± SD (n = 3); **** p < 0.0001. **G)** Structure of the proximal *LARGE2* genomic locus (UCSC Browser) showing a potential TCF7L2 binding site (L2_BS) in the first *LARGE2* intron. * Source: HCT-116 TCF7L2 UC Davis ChIP-seq Signal from ENCODE/SYDH (Peggy Farnham lab). **H)** qChIP analysis on genomic DNA from SW480 cells. The amount of DNA immunoprecipitated with TCF7L2 antibody or rabbit IgG-control in each sample is shown as percentage of chromatin input. Results are shown as mean ± SD (n = 3); ** p < 0.01; **** p < 0.0001. **I)** qRT-PCR analysis of indicated genes in stably transduced SW480 cells upon CRISPR/eCas9-mediated targeting of the TCF7L2_BS in the first intron of *LARGE2* via two different guide RNAs. Results are shown as mean ± SD (*n* = 5); **** p < 0.0001

The 1555 amino acid variant of APC in HT-29 and Colo205 CRC cells encompasses three 20 AARs (3*20AAR) sufficient for partial β-catenin inactivation, which translates to low cell-intrinsic Wnt activity [4, 36]. Stable transduction with lentiviruses encoding for a doxycycline (DOX)-inducible short hairpin RNA (shRNA) allowed us to silence *APC* mRNA and protein in these cell lines **(**Fig. 1A,B and Additional file 2A-C**)**. *APC* silencing augmented expression of bona-fide Wnt target genes, such as *AXIN2* [37] and *LGR5* [38] (Additional file 2A,C). Next generation sequencing (NGS, RNA-Seq) 72 h after shRNA-mediated *APC* silencing in HT-29 cells revealed 205 upregulated genes, while the expression of 82 genes was downregulated (> 4 fold change, *p*-value < 0.01) (Fig. 1C and Additional file 3). Besides the known Wnt targets *AXIN2*, *NOTUM*, *SP5*, *NKD1*, and *ASCL2,* we found that additional candidate genes, such as *PTK7* and *LARGE2*, were upregulated in this setting (Fig. 1C and Additional file 3).

### *LARGE2* represents a direct target of Wnt signaling in CRC

Since a direct link between canonical Wnt signaling and O-glycosylation has not been reported yet to our knowledge, we focused our work on *LARGE2*, a gene encoding for a bifunctional O-glycosyltransferase [22]. Induction of *LARGE2* and bona-fide Wnt target genes upon *APC* silencing in HT-29 and Colo205 cells was confirmed by quantitative real-time PCR (qRT-PCR) (Fig. 1D and Additional file 2C, D). Expression of *LARGE1*, the paralog of *LARGE2*, was low (Ct values of 32–33 versus ~ 28 for *LARGE2*) in these cells, and *LARGE1* was not affected by *APC* silencing (Fig. 1D). Since APC possesses functionalities beyond controlling Wnt activity [39], we stably transduced HT-29 cells with lentiviruses carrying a DOX-inducible allele of oncogenic β-catenin (CTNNB1-S33Y) [40]. CTNNB1-S33Y triggered expression of *LARGE2,* suggesting that *LARGE2* is driven by canonical Wnt signaling (Fig. 1E). To interfere with β-catenin/TCF functionality in Wnt-active CRC cell lines, we stably transduced LS174T and SW480 to express the CTNNB1-binding domain of TCF7L2 (nTCF: amino acids 1 to 90) fused to a tamoxifen-inducible version of the hormone-binding domain of the estrogen receptor (ERT2) (here referred to as nTCF-ERT2 or, as a cell line suffix, −NE). Cells stably expressing the ERT2 domain (referred to as -ERT) alone served as a control. Upon addition of 4-hydroxy-tamoxifen (4-OHT) to cells expressing nTCF-ERT2, which then sequesters nuclear β-catenin to nTCF7 unable to bind DNA, *LARGE2* expression was downregulated to a similar extent as *LGR5* and *AXIN2* when compared to ERT2 control cells (Fig. 1F**,** Additional file 2E,F).

In silico analysis of putative TCF7L2 genomic DNA binding loci in HCT116 CRC cells via the UCSC genome browser revealed DNA occupation by TCF7L2 within the first intron of *LARGE2* (ENCODE annotation data [41], Farnham-USC, Accession No: ENCSR000EUV) (Fig. 1G). Quantitative chromatin immunoprecipitation (qChIP) analysis in SW480 cells confirmed binding of TCF7L2 to the first intron of *LARGE2*, which contains a canonical TCF7L2 binding motif CTTTGATC [42] (Fig. 1G,H). A second potential binding motif ~ 5 kb upstream of *LARGE2* did not show occupation by these factors (Fig. 1G,H). To demonstrate the specificity of our qChIP assay, we confirmed TCF7L2 within the *LGR5* and *SP5* promoters, while an amplicon upstream of *c-MYC* previously shown to lack TCF7L2 and β-catenin occupancy [43] was not enriched (Fig. 1H). We next assessed the functionality of the TCF7L2 binding site within *LARGE2* (L2_BS) by performing CRISPR/Cas9-mediated mutagenesis: Stable delivery of a *Streptococcus pyogenes* Cas9 derivative engineered for improved specificity (referred to as eCas9) [44] plus two different guideRNAs (BSg1 and BSg2) recognizing a sequence either on the (+) or the (−) strand of the L2_BS was achieved by lentiviral transduction. As a control, a non-targeting tracrRNA was used. Successful targeting of the L2_BS in stably transduced SW480 cell pools was confirmed by a mutation detection assay (Additional file 2G). Indeed, CRISPR/eCas9-mediated L2_BS targeting led to 4- to 5-fold reduced *LARGE2* mRNA levels and abolished L2_BS occupation by TCF7L2 in SW480 (Fig. 1I and Additional file 2H). Accordingly, site directed mutagenesis of the “CTTTGATC” TCF7L2 binding motif to CTTTGGCC [45] within a ~ 600 bp ectopic DNA fragment of *LARGE2* compromised the activity of a luciferase reporter (Additional file 2I). Overall, our data suggest that Wnt signaling triggers *LARGE2* gene expression via the β-catenin/TCF7L2 transcriptional complex in CRC.

### *LARGE2* correlates with active Wnt signaling and hCoSC gene expression in CRC

**Fig. 2:**
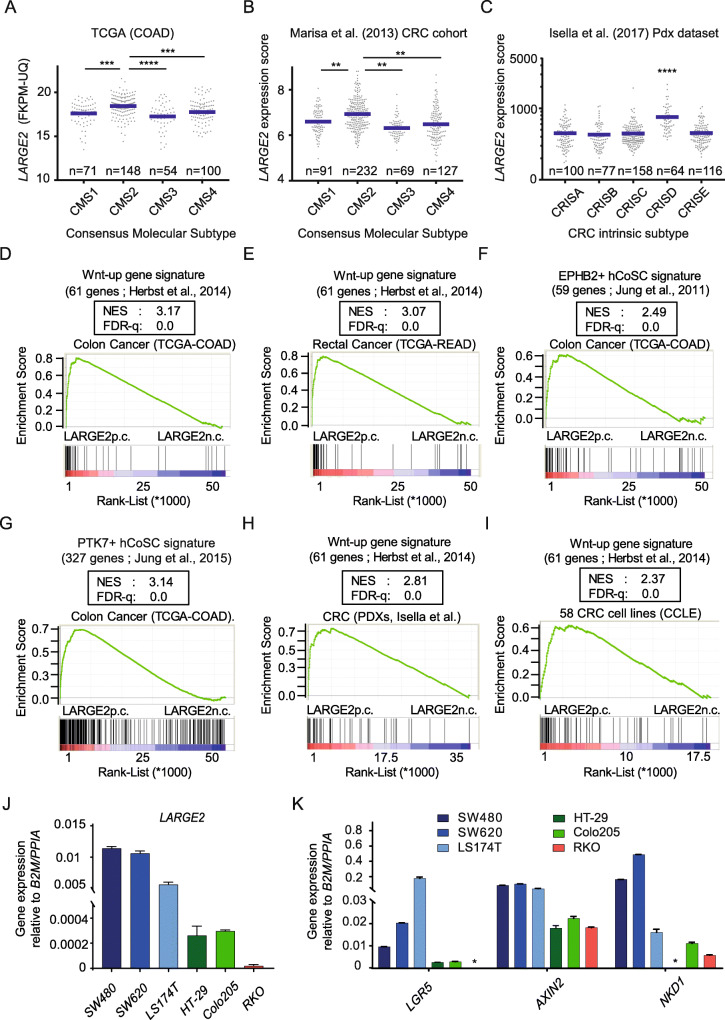
*LARGE2* mRNA levels in CRC correlate with high Wnt activity and hCoSC gene expression. **(A-C)***LARGE2* gene expression in consensus molecular subtypes (CMS) of the TCGA-COAD cohort (*n* = 373) **(A)**, the Marisa et al. (2013) CRC cohort (*n* = 519) **(B)**, and in the CRC intrinsic subtypes (CRIS) of a PDX cohort published by Isella et al. (2017) (*n* = 515) **(C)**. Shown are the mean levels of *LARGE2* (blue horizontal line). Asterisks indicate a significant difference between CMS2 or CRISD and the other subtypes. Significance was calculated by one-way ANOVA testing (** *p* < 0.01, *** *p* < 0.001, **** p < 0.0001). **D-G)** GSEA on *LARGE2* gene signatures derived from TCGA-COAD and TCGA-READ RNA-Seq data sets. Shown are enrichments of Wnt target gene sets upregulated upon siRNA-mediated β-catenin silencing in CRC cells (**D, E**), and gene sets which specify EPHB2^high^**(F)** or PTK7^high^ (**G**) hCoSCs (see method section for details). **NES**: normalized enrichment score, **FDR-q**: False discovery rate q-value. **H,I**) GSEA on *LARGE2* gene signatures derived from microarray data of a PDX CRC cohort published by Isella et al. 2017 (**H**) and the 58 CRC lines included in the CCLE database (**I**). p.c.: positive correlation, n.c.: negative correlation. **J,K)** qRT-PCR analysis of *LARGE2* gene expression (**J**) or expression of bona-fide Wnt target genes (**K**) in the indicated CRC cell lines. Analysis was performed in duplicates (two independent RNA samples per cell line). Error bars indicate ±SD. * gene expression not detectable by qRT-PCR

Considering a well-accepted segregation of colon cancer into four distinct sub-groups known as the consensus molecular subtypes (CMS) [31], we wondered if expression of *LARGE2* was associated with one of these categories. When analyzing two independent, CMS classified colon cancer patient cohorts (TCGA-COAD [46] and Marisa et al. (2013) [29]), we found highest *LARGE2* mRNA levels in CMS2, specified by an active WNT/MYC program [31] (Fig. 2A,B). Since contaminating stroma cells within tissue samples were shown to bias this transcriptional classification of CRC [47, 48], Isella and colleagues defined five purely epithelial CRC Intrinsic Subtypes (CRIS A-E) from gene expression profiles of 515 xeno-transplanted human tumor samples (PDXs), which they had derived from 244 individuals suffering from CRC [30]. By analyzing these data, we found *LARGE2* gene expression was explicitly enriched in CRIS-D tumors characterized by an intestinal stem cell (ISC) phenotype and high Wnt activity [30] (Fig. 2C). Moreover, Isella and colleagues used their algorithm to re-stratify the TCGA-COAD [46] and the Marisa et al.(2013) [29] cohorts into the five CRIS classes [30]. Also here, *LARGE2* was enriched in the highly Wnt-active and ISC-like CRIS subtype D (Additional file 4A,B).

Next, we used NGS RNA-Seq data derived from 458 colon cancer and 167 rectal tumors from TCGA as provided by the NCI-GDC [28], and we generated pre-ranked lists of genes according to their Pearson correlation with *LARGE2* gene expression (data not shown). By GSEA, we found that two independent sets of Wnt target genes [15, 49] and a recently described oncogenic/intrinsic Wnt signature [50] were enriched among the genes positively correlated with *LARGE2* expression (Fig. 2D,E and Additional file 4C-E). Since Wnt signaling represents a major driver of stemness in colonic epithelium and CRC [51], we studied the enrichment of two published gene sets specifying EPHB2^high^ and PTK7^high^ hCoSCs [2, 34] in the *LARGE2* signature of CRC: Both hCoSC-specific gene sets were enriched among the genes positively correlated with *LARGE2* expression (Fig. 2F,G and Additional file 4F,G). We obtained similar results with a pre-ranked list of *LARGE2* correlated genes derived from the CRIS gene expression data set (*n* = 515 PDX CRC samples) [30] (Fig. 2H and Additional file 4H-J) and from microarray data (*n* = 58 established CRC cell lines) obtained from the Cancer Cell Line Encyclopedia (CCLE) [52] (Fig. 2I and Additional file 4 K,L). Accordingly, HT-29 and Colo205, specified by low intrinsic Wnt activity [36], and RKO cells, wild-type for *APC* and *CTNNB1* [53], displayed lower levels of *LARGE2*, *LGR5, AXIN2,* and *NKD1* expression when compared to highly Wnt-active SW480 and SW620 cells which harbor shorter APC variants [4], or LS174T which express oncogenic mutant β-catenin [54] (Fig. 2J,K). These data show that *LARGE2* is enriched in colorectal tumors characterized by active Wnt signaling, an ISC phenotype, and a hCoSC gene expression program.

### LARGE2 is essential and sufficient for matriglycan formation on α-DG in CRC

**Fig. 3:**
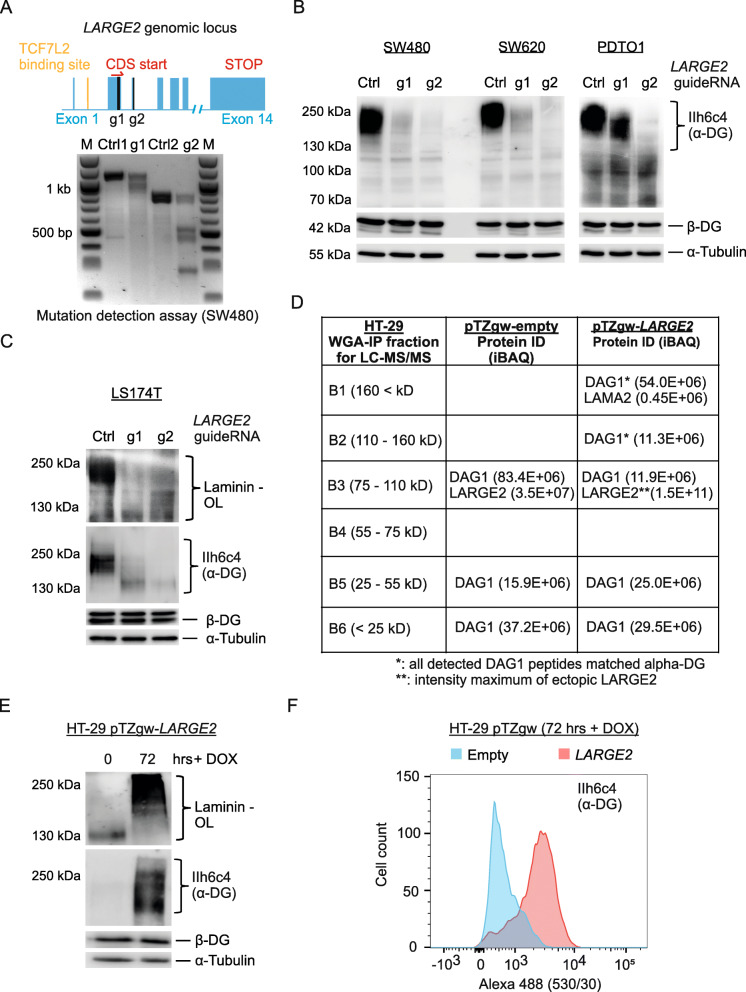
LARGE2 mediates functional O-glycosylation of α-DG in CRC. **A) Upper panel**: *LARGE2* exons indicated as blue bars. GuideRNA 1 and 2 (g1, g2) target sites in black, TCF7L2 binding site in yellow, start of coding sequence (CDS start) in Exon2 and STOP in Exon 14 (both in red). **Lower panel**: Mutation detection assay on genomic DNA from SW480 cells edited via CRISPR/Cas9 to achieve *LARGE2* KO (g1, g2) or control (Ctrl). M: Marker. **B)** KO of *LARGE2* by CRISPR/Cas9 in SW480 and SW620 cells (left panel) and PDTO1 (right panel) using g1 or g2. Western Blot analysis of WGA-enriched glycoproteins was used to detect α-DG, WCL were used to detect β-DG and tubulin. **C)** Immunoblot analysis and laminin overlay (Laminin-OL) of WGA-enriched glycoproteins after KO of *LARGE2* via CRISPR/Cas9 in LS174T cells. β-DG and tubulin were analyzed on WCLs. **D)** LC-MS/MS analysis on HT-29 control (empty) or *LARGE2* over-expressing cells. Cell lysates enriched for glycoproteins were run on SDS-PAGE and divided into 6 MW fractions B1-B6. Proteins of interest (DAG1, α-DG, LARGE2, and LAMA2) and their iBAQ (Intensity Based Absolute Quantification) are shown. **E)** Immunoblot analysis and laminin overlay (Laminin-OL) of WGA-enriched glycoproteins upon conditional ectopic expression of *LARGE2* in HT-29 cells (500 ng/ml DOX for 72 h). WCL were used to detect β-DG and tubulin. **F)** Flow cytometry analysis on control infected HT-29 cells (Empty, blue profile) or *LARGE2* over-expressing cells (red profile) after treatment with DOX for 72 h. Cells were stained with IIh6c4 antibody and anti-mouse Alexa 488 secondary antibody. See Additional file 6E for secondary antibody control

To address the relevance of LARGE2 for functional α-DG O-glycosylation in CRC, we generated targeted knock-outs (KOs) of *LARGE2* in CRC cell lines and a patient-derived tumor organoid (PDTO) line PDTO1 (Additional file 5) by CRISPR/eCas9-mediated genome editing using two guide RNAs directed against different regions of the *LARGE2* open reading frame (Fig. 3A, upper panel). Due to the lack of commercially available antibodies against LARGE2, we confirmed successful gene editing by performing a mismatch detection assay (Fig. 3A**,** lower panel). Next, we purified glycoprotein-enriched fractions from *LARGE2* wild-type and *LARGE2* KO whole cell lysates (WCLs) via wheat germ agglutinin (WGA) agarose-affinity enrichment (−AE). Immunoblot analysis with an O-glycosylation sensitive antibody against α-DG (IIh6c4) revealed that targeting *LARGE2* indeed diminished O-glycosylated α-DG at ~ 150–250 kDa in SW480, SW620 (Fig. 3B**,** left panel), PDTO1 (Fig. 3B, right panel), and LS174T (Fig. 3C). Transient delivery of Cas9 plus a sgRNA against *LARGE2* in SW620 cells yielded the same result (Additional file 6A). Importantly, *LARGE2* targeting abolished laminin binding capacity of WGA-AE purified glycoprotein fractions in the laminin overlay assay, suggesting that LARGE2 is essential to create functional matriglycan on α-DG in CRC (Fig. 3C). Loss of LARGE2 did not affect *DAG1* mRNA or β-dystroglycan (β-DG) protein (Fig. 3B,C, Additional file 6A,B**)**.

To answer if LARGE2 alone was sufficient for matriglycan formation on α-DG in CRC cells characterized by low Wnt activity and hence low endogenous *LARGE2* levels, we stably equipped HT-29 cells with a DOX-inducible *LARGE2* cDNA by lentiviral transduction. In this model, addition of DOX for 72 h led to ~ 10–25 fold higher levels of *LARGE2* mRNA when compared to Wnt-active SW480 and PDTO1 cells or Wnt-activated HT-29 cells (Additional file 6C). Since ectopic LARGE2 has been reported to also O-glycosylate laminin-binding and IIh6c4-reactive glypican 4 (GPC4) in mouse ES cells [55], we performed quantitative mass spectrometry (LC-MS/MS) analysis on WGA-AE purified glycoproteins from these cells. Due to the shift in molecular weight (MW) of proteins upon O-glycosylation by LARGE2, we fractionated electrophoretically separated samples into 6 MW windows prior to LC-MS/MS analysis (Additional file 6D). LARGE2 was detected in the 75–110 kDa window, in accordance with its calculated MW of 81.8 kDa, and DAG1 peptides from control HT-29 cells occurred at 0–55 kDa and 75–110 kDa (Fig. 3D). Upon ectopic expression of LARGE2, peptides matching the α-DG domain of DAG1, but not β-DG, appeared at 110–160 kDa and with higher intensity at 160–300 kDa (Fig. 3D, Additional file 7**)**. This 160–300 kDa fraction also contained unique peptides for LAMA2 (Laminin subunit alpha 2), but LAMA2 was absent in control samples **(**Fig. 3D**)**. This shows that HT-29 cells express LAMA2, which, upon LARGE2-mediated O-glycosylation of α-DG, co-precipitates with the WGA-α-DG complex. Notably, only α-DG-specific peptides consistently shifted over two MW windows with high intensity upon *LARGE2* overexpression, and GPC4 was overall undetectable in HT-29 cells. This points to α-DG as the main substrate of LARGE2 in this cellular background and verified our findings from immunoblot analysis using the O-glycosylation sensitive antibody IIh6 and a laminin-overlay assay on WGA-AE purified protein fractions (Fig. 3E). Furthermore, elevated cell surface abundance of O-glycosylated α-DG upon ectopic LARGE2 expression was visualized by flow cytometry-assisted cell sorting (FACS) analysis after live cell immunolabelling (Fig. 3F, Additional file  6E). Our findings suggest that LARGE2 is both essential and sufficient for the attachment of laminin-binding matriglycan on α-DG in CRC.

### Wnt signaling modulates functional O-glycosylation of α-DG via induction of *LARGE2* in CRC

**Fig. 4:**
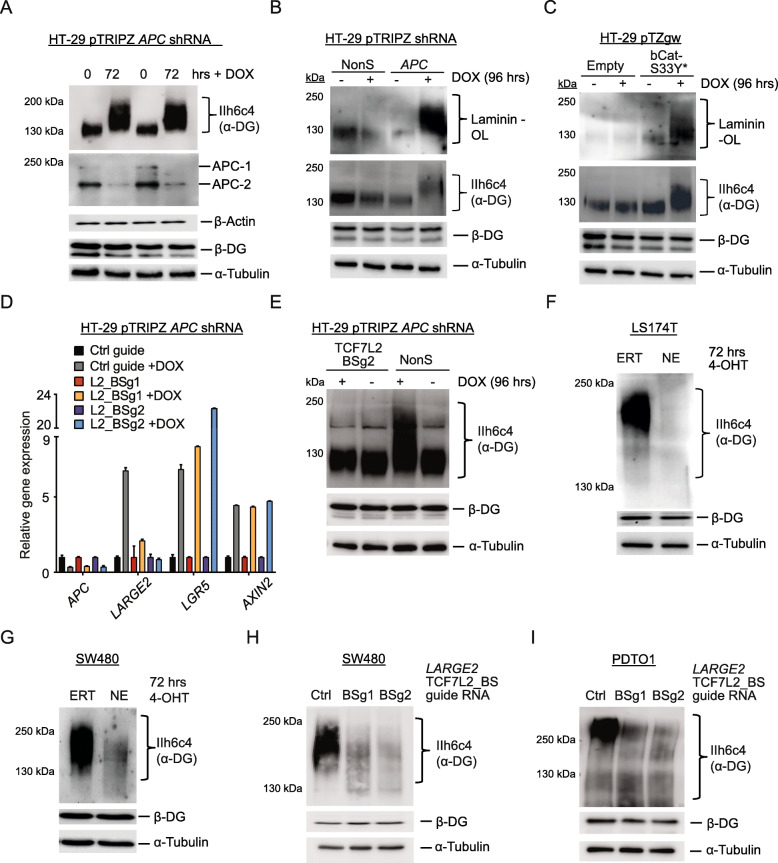
Wnt signaling modulates LARGE2-dependent O-glycosylation of α-DG in CRC. **A)** Immunoblot analysis of O-glycosylated α-DG (WGA-AE purified). APC, β-DG, and α-tubulin were analyzed on WCL from HT-29 cells after silencing of *APC* for 72 h. **B,C)** Immunoblot analysis and laminin overlay (OL) on WGA-AE purified α-DG from HT-29 cells after silencing of *APC***(B)** or ectopic expression of CTNNB1-S33Y **(C)**. β-DG and α-tubulin were analyzed on WCL. **D)** qRT-PCR analysis of indicated genes in HT-29 cells, carrying a DOX-inducible shRNA of APC, after CRISPR/Cas9-mediated targeting of the TCF7L2-BS (L2_BSg1 and g2) in *LARGE2* intron 1. Error bars indicate SD (n=3). **E)** Immunoblot analysis of O-glycosylated α-DG (WGA-AE purified) in HT-29 wild-type or mutant for the TCF7L2-binding site in *LARGE2* intron 1 (L2_BSg2) after silencing of APC for 96 h. WCLs were used to detect β-DG and tubulin. **F,G)** Immunoblot analysis of WGA-AE purified O-glycosylated α-DG in LS174T-NE **(F)** and SW480-NE cells **(G)** relative to their ERT2 controls upon treatment with 400 nM 4-OHT for 72 h. WCLs were used to detect β-DG and tubulin. **H,I)** Immunoblot analysis on WGA-AE purified α-DG from SW480 **(H)** and PDTO1 **(I)** cells upon CRISPR/eCas9-mediated targeting of the TCF7L2-BS in the first intron of *LARGE2* via guideRNAs. β-DG and α-tubulin were analyzed on WCL

We next asked whether modulation of Wnt signaling would affect LARGE2-dependent functional O-glycosylation of α-DG. Indeed, shRNA-mediated silencing of *APC* or conditional ectopic expression of oncogenic CTNNB1-S33Y in HT-29 cells increased α-DG O-glycosylation (Fig. 4A-C) and laminin binding capacity on the overlay assay (Fig. 4B,C). Importantly, *LARGE2* mRNA induction and the increased MW matriglycan formation on α-DG upon *APC* silencing depended on the integrity of the endogenous TCF7L2-BS within intron 1 of *LARGE2* in CRISPR/Cas9-edited HT-29 cell lines (Fig. 4D,E, see Additional file 6F for Sanger sequencing results on the mutated TCF7L2-BS). In accordance, interfering with β-catenin/TCF functionality in SW480-NE and LS174T-NE led to reduced levels of O-glycosylated α-DG when compared to ERT2 control cells (Fig. 4F,G**)**. *DAG1* mRNA and β-DG protein did not significantly change upon Wnt modulation in CRC cells (Fig. 4A-G, Additional file 6G,H). Furthermore, CRISPR/eCas9-mediated targeting of the TCF7L2-BS within *LARGE2* in SW480 and in the CRC organoid line PDTO1 (Additional file 5), which led to decreased *LARGE2* mRNA levels (Fig. 1I and Additional file 6I), compromised O-glycosylation of α-DG (Fig. 4H,I). Taken together, these data suggest that Wnt signaling regulates the functional O-glycosylation of α-DG in CRC cells, and this process depends on Wnt/TCF7L2-mediated induction of *LARGE2* gene expression.

### *LARGE2* gene expression and α-DG O-glycosylation are enriched in the Wnt-driven stem/progenitor compartment of human colonic epithelium

**Fig. 5:**
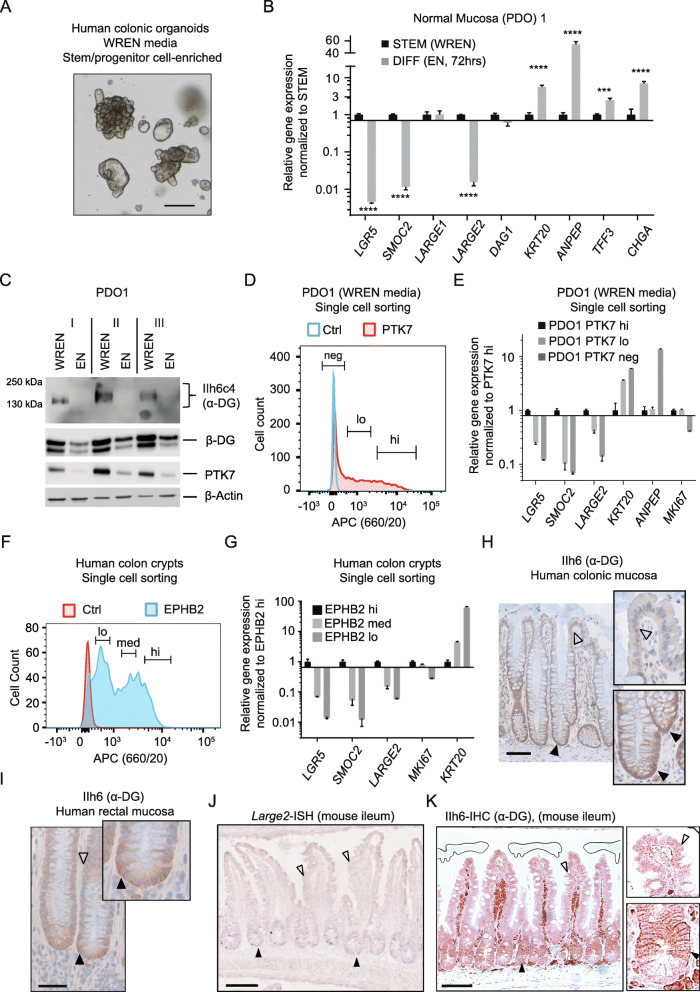
*LARGE2* expression of α-DG O-glycosylation are enriched in the Wnt-driven stem/progenitor compartment of human colon epithelium. **A)** Human colonic organoids (PDOs) embedded in Matrigel and maintained in WREN (Wnt, R-Spondin, EGF, Noggin) medium. Scale bar represents 20 μm. **B)** qRT-PCR analysis of the indicated genes in PDO1 maintained in WREN (=STEM) or differentiation medium (DIFF, EN). Results are shown as mean ± SD (*n* = 3). *** *p* < 0.001; **** *p* < 0.0001. See Additional file 8A,B for experiments on PDO2 and PDO3. **C)** Immunoblot analysis of α-DG from WGA-AE purified glycoproteins of PDO1, cultivated in WREN or EN media for 72 h. WCL were used for analysis of β-DG, PTK7, and β-actin. **D-G)** FACS profile and Taqman™ qRT-PCR analysis from PDO single cells -stained with APC-coupled antibody for PTK7 **(D,E)** or from human crypt epithelial cells, stained against EPHB2 **(F,G).** Control staining for viable cells (DAPI) was performed to define the PTK7 or EPHB2-negative fraction (Ctrl). **In D**) **neg**: PTK7 negative, **lo**: PTK7 low, **hi**: PTK7 high. **In F) hi**: EPHB2 high, **med**: EPHB2 medium, **low**: EPHB2 low cell fraction. Error bars in **E** and **G** indicate mean ± SD (n = 3 technical replicates). See Additional file 8D,E for data on PDO2 and PDO3. **H,I)** Immunohistochemistry (IHC) analysis of α-DG (IIh6 antibody) on FFPE human colonic **(H)** or rectal **(I)** mucosa. Black arrowheads: crypt base specific staining; clear arrowheads: fading or loss of staining. Scale bars represent 100 μm (**H**) and 50 μm (**I**). **J, K)** In situ hybridization (ISH) with a *Large2*-specific probe **(J)** and IHC staining of glycosylated α-DG **(K)** on mouse small intestinal FFPE tissue (Ileum). Scale Bar represents 100 μm. Black arrowheads: staining in crypts; clear arrowheads: lack of staining in villi. See Additional File 9 for control staining (no probe) and additional data

To analyze the status of *LARGE2* and α-DG in self-renewing hCoSCs, which depend on active Wnt signaling [56, 57], we isolated human colonic crypts from fresh tissue specimen and propagated them as patient-derived hCoSC-enriched organoids (PDOs) in a 3-dimensional (3-D) matrix (Matrigel®) overlaid with self-renewal promoting WREN culture media (Wnt3a, R-Spo3, EGF, Noggin), similar to what has been described previously (Fig. 5A) [2, 23]. Indeed, PDOs displayed expression of *LARGE2* (qRT-PCR: Ct values 24–25 versus 32–33 for *LARGE1*) and, as visualized by immunoblotting, IIh6c4-reactive O-glycosylated α-DG at a MW of ~ 130–140 kDa (Fig. 5B,C, Additional file 8A-C). This MW of α-DG was lower than what we had observed in LS174T, SW480/620, and PDTO1 (Fig. 3B,C). Transfer of PDOs to culture medium devoid of Wnt and R-Spondin factors (EN medium), which triggers multi-lineage differentiation [2, 23], strongly decreased *LARGE2* mRNA levels, similar to expression of bona-fide ISC markers *LGR5* [38] and *SMOC2* [58] (Fig. 5B**,** Additional file 8A,B). As expected, expression levels of *KRT20* (pan-differentiation), *ANPEP* (enterocytes), *TFF3* (goblet cells), and *CHGA* (enteroendocrine cells) were increased (Fig. 5B, Additional file 8A,B). In contrast to *LARGE2*, *LARGE1* and *DAG1* mRNA levels did not change during PDO differentiation (Fig. 5B, Additional file 8A,B). Importantly, differentiation of PDOs, indicated by lower levels of the hCoSC marker PTK7 [34], abolished α-DG O-glycosylation (Fig. 5C**,** Additional file 8C). Interestingly, we also observed a reduction of β-DG protein upon PDO differentiation (5C, Additional file 8C).

As has been reported previously, human PDOs maintained in WREN medium display a heterogeneity of stem and progenitor cells specified by relatively high or low surface abundance of the hCoSC marker PTK7 [34]. To examine *LARGE2* expression in these PDO sub-populations, we performed FACS sorting to isolate PTK7-high, PTK7-low, and PTK7-negative cells from PDOs derived from three different individuals: Strongest *LARGE2* gene expression occurred in PTK7-high cells, which also showed higher levels of *LGR5* and *SMOC2* mRNA when compared to PDO cells low or negative for PTK7 (Fig. 5D,E, Additional file 8D,E). Lowest *LARGE2* expression was detected in PTK7-negative PDO cells characterized by relatively highest levels of the differentiation markers *KRT20* and *ANPEP* (Fig. 5E, Additional file 8D,E). We received a similar result after sub-fractionation of primary mucosal tissue-derived crypt epithelial cells by FACS according to the surface abundance of EPHB2, which is highest in hCoSCs located at the crypt bottom [2] (Fig. 5F). Here, expression of *LARGE2*, *LGR5* and *SMOC2* was most pronounced in the stem cell-enriched EPHB2-high fraction and stepwise declined in EPHB2-medium and EPHB2-low cells which represent transient amplifying cells at different stages of differentiation [2] (Fig. 5G). As expected, *KRT20* levels showed an inverse gradient with highest expression in the EPHB2-low cell fraction (Fig. 5G). Next, we performed immunohistochemical (IHC) staining on formalin-fixed paraffin-embedded (FFPE) human colonic and rectal tissue for O-glycosylated α-DG with the glycosylation-sensitive antibody IIh6. IHC staining was optimized on FFPE sections of human heart muscle tissue (Additional file 8F,G). By applying this protocol, we observed strongest immuno-reactivity against O-glycosylated α-DG at the membrane of stem/progenitor cells of human colonic and rectal crypts while differentiated upper crypt cells did not show this feature (Fig. 5H,I). These data suggest that LARGE2/α-DG signaling is differentiation-dependent in human colonic epithelium and mainly occurs in the Wnt-driven stem/progenitor cell compartment.

### A differentiation-dependent LARGE2/α-DG axis is conserved in mouse small intestinal epithelium

To address if the differentiation dependent expression of *LARGE2* observed in human PDOs was conserved among species, we performed in situ hybridization (ISH) with a *Large2*-specific probe on FFPE sections of mouse intestinal (ileum) tissue. Mild expression of *Large2* mRNA mainly occurred at the bottom two-thirds of intestinal crypts, while intestinal villi, which harbor terminally differentiated cells, did not show expression of *Large2* (Fig. 5J, Additional file 9A). We observed a similar gradient of membranous O-glycosylated α-DG by performing IHC on a serial FFPE section (Fig. 5K**,** see Additional file 9B for antibody controls on mouse tissue). Therefore, *Large2* mRNA levels and O-glycosylation of α-DG correlate with a non-differentiated status of mouse small intestinal epithelial cells.

### *LARGE2* expression and O-glycosylation of α-DG depend on *APC* functionality in human adenoma organoids

**Fig. 6:**
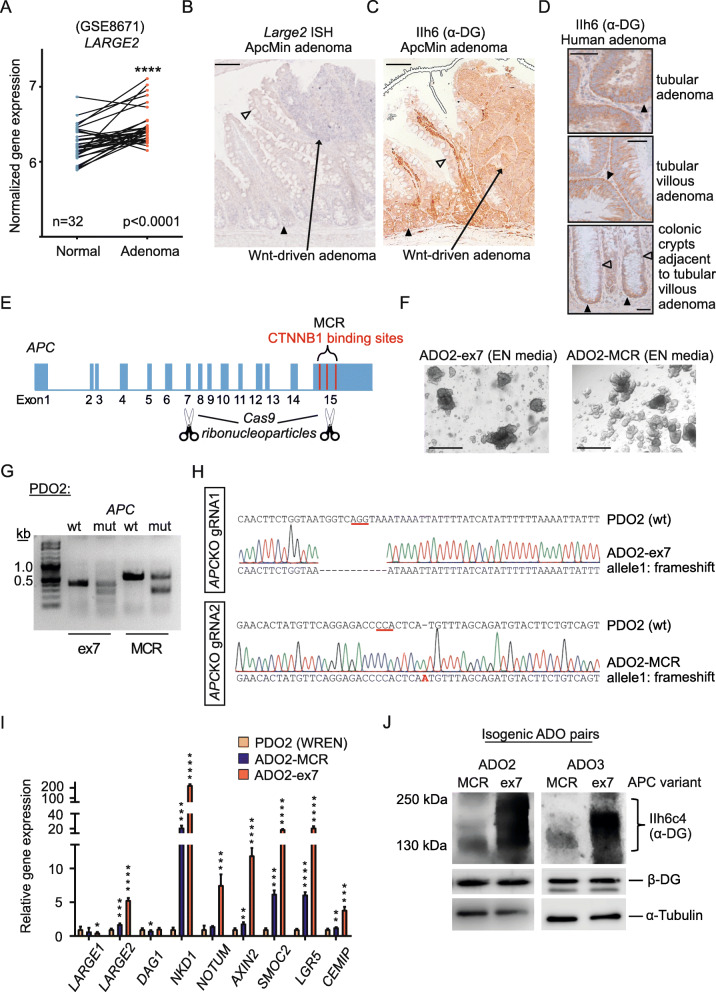
*LARGE2* expression and α-DG O-glycosylation in engineered human adenoma organoids depends on the functionality of truncated APC. **A)***LARGE2* gene expression in a human dataset GSE8671 (*n* = 32), comparing normal human mucosa to matched adenoma tissue (**** p < 0.0001). **B)** ISH analysis using a *Large2*-specific probe on APCmin mouse FFPE adenoma tissue and adjacent intestinal crypts/villi (ileum). Black arrowhead: staining in normal crypts. Clear arrowheads: absent staining. Scale bar represents 100 μm. See Additional file 9E for control. **C,D)** IHC staining for α-DG (IIh6 antibody) on Wnt-driven adenoma tissue of an APCmin mouse (**C**) or on human adenoma and adjacent normal tissue (**D**). Black arrowheads: membranous staining in crypt and adenoma regions, clear arrowheads: absent staining in upper crypts. Scale bar represents 100 (**C**) and 20 μm (**D**). **E,F)***APC* gene structure, *CTNNB1* binding sites (20AAR sequences) in Exon 15. Two Cas9 ribonucleoparticles were assembled to target *APC* of PDOs in either Exon 7 (ex7) or 15 (MCR) (**E**), which yielded two isogenic adenoma organoid lines (ADO-ex7 and ADO-MCR) maintained in EN media (**F**). Scale bars represent 500 μm. **G, H)** Mutation detection assay (**G**) and Sanger sequencing (**H**) on genomic DNA derived from human normal mucosa (PDO2) organoids and isogenic adenoma organoid lines (ADO-ex7 and ADO-MCR). **I)** qRT-PCR analysis of the indicated genes from normal colon organoids (PDO2, in WREN media) and the isogenic ADO lines (ADO2-MCR and ADO2-ex7). Gene expression is relative to PDO2. Samples derived from one patient were analyzed three times. The results are shown as mean ± SD. ** *p* < 0.01; *** p < 0.001; **** p < 0.0001. See Additional File 9F-H for data on PDO3. **J)** Immunoblot analysis of glycosylated α-DG on WGA-AE purified glycoproteins from two isogenic adenoma organoid pairs ADO2 and ADO3 (ex7 versus MCR) WCL was used for detection of DAG1, β-DG, and tubulin

According to public microarray data derived from 32 patient-matched human colonic mucosa-adenoma pairs (GSE8671) [59], *LARGE2* levels were slightly elevated in human adenoma specimen (Fig. 6A). ISH against *Large2* on FFPE sections derived from ApcMin mice [60] revealed elevated abundance of *Large2* mRNA in adenoma (Fig. 6B**,** Additional file 9C,D), which also showed membranous O-glycosylated α-DG as visualized by IHC staining (Fig. 6C). In human adenoma, we detected basal and membranous α-DG via IHC staining on FFPE sections of tubular and tubular-villous adenomas which are typically driven by an intrinsically activated Wnt program (Fig. 6D). Colonic crypts in the vicinity of the dysplastic tissue displayed the expected restriction of O-glycosylated α-DG to the stem/progenitor cell compartment at the crypt bottom (Fig. 6D**,** lower panel).

We next asked whether different truncation mutations of *APC* would differentially affect the level of *LARGE2* and O-glycosylated α-DG. Therefore, we performed CRISPR/Cas9-mediated genome editing on two benign PDO lines to introduce truncation mutations either in the MCR (Exon 15, guide RNA as described in [33]) or in Exon 7/8 (guide RNA as described in [61]) of *APC* (Fig. 6E). As a selective measure, engineered human adenoma organoid lines (ADOs) were propagated in culture media lacking Wnt and RSPO factors (EN media) (Fig. 6F). Genome editing was confirmed by a mismatch cleavage assay and by Sanger sequencing (Fig. 6G,H, Additional file 9F,G). In those ADOs where *APC* had been mutated in the MCR (ADOs-MCR), so as to maintain 2*20AAR repeats in APC, expression of known Wnt targets was markedly lower when compared to ADOs carrying the short APC variant (ADOs-ex7) (Fig. 6I, Additional file 9H). Importantly, the *APC* exon7 truncation mutation caused a more than 3-fold higher expression of *LARGE2* when compared to ADOs-MCR, while expression levels of *LARGE1* and *DAG1* were equal in both types of ADOs (Fig. 6I, Additional file 9H). Importantly, elevated levels of *LARGE2* in ADOs-ex7 were associated with higher MW matriglycan structures on α-DG (between 130 and 250 kDa) when compared to their *APC*-MCR mutant but otherwise isogenic counterparts (~ 130–150 kDa) (Fig. 6J), or PDOs (~ 130–150 kDa, Fig. 5C), while β-DG levels were equal in the two ADO types (Fig. 6J). These data show that complete functional ablation of APC leads to aberrantly activated Wnt/LARGE2/α-DG signaling in human pre-malignant ADOs.

### *LARGE2* gene expression is overall elevated in full-blown primary and liver metastatic CRC

**Fig. 7:**
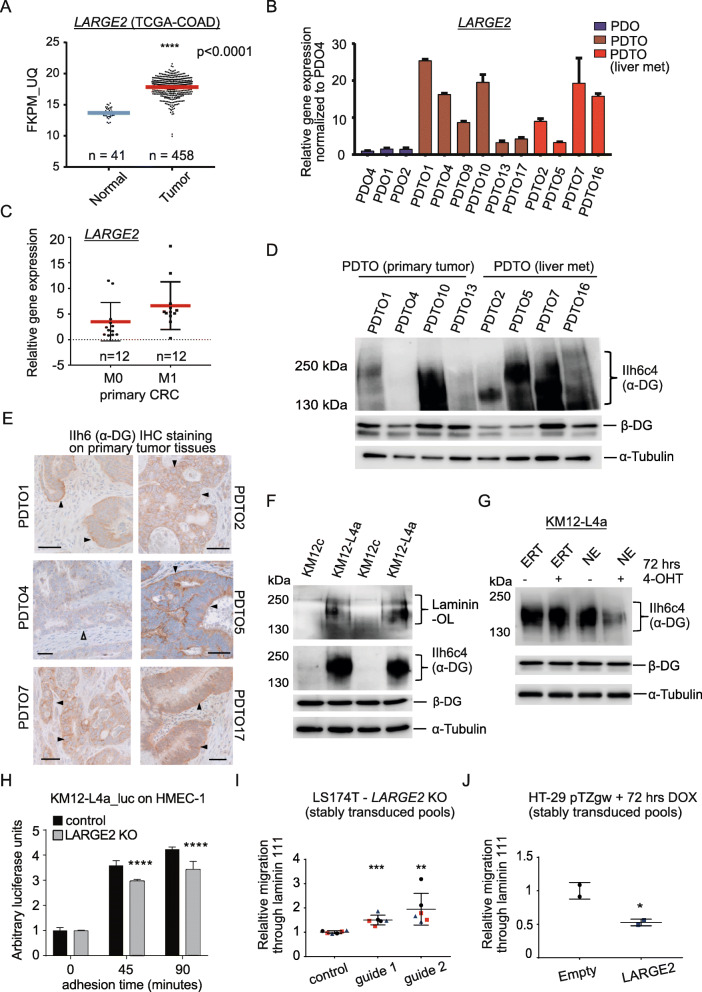
*LARGE2/*α-DG signaling is elevated in primary and liver metastatic CRC cells and affects CRC cell adhesion and migration. **A)***LARGE2* gene expression analysis on human TCGA-COAD dataset**:** comparison of normal human mucosa samples and tumor tissues. (**** p < 0.0001). **B)** qRT-PCR analysis of *LARGE2* in PDOs and PDTOs: colonic organoids (PDOs, blue), primary tumor organoids (PDTO, dark red), and liver metastasis-derived tumor organoids (mPDTO, light red). Shown are mean values ± SD (n = 3 technical replicates). **C)** TaqMan™ qRT-PCR analysis of *LARGE2* expression on primary FFPE tissue samples comparing M0 and M1 CRC cases. **D)** Immunoblot analysis of glycosylated α-DG on WGA-AE purified glycoproteins from primary and metastatic (m)PDTOs. β-DG and α-Tubulin were detected on input WCL. **E)** IHC analysis of glycosylated α-DG (IIh6 antibody) on FFPE CRC tissues matching to the indicated PDTOs. Black arrowheads: specific staining. Clear arrowhead: negative staining. Scale bars: 50 μm. **F,G)** Immunoblot analysis of glycosylated α-DG and laminin overlay (Laminin-OL) on WGA-AE purified glycoproteins from KM12c and KM12-L4a cells (**F**) and from KM12-L4a-NE or -E cells non-treated or after treatment with 400 nM 4-OHT for 72 h (**G**). β-DG and α-Tubulin were detected on WCL. **H)** Adhesion of KM12-L4a cells, stably transduced with a luciferase (luc)-encoding lentivirus and either wild-type or LARGE2 KO, was quantified at the indicated time points by measuring luc activity. T = 0 represents the baseline control and was also used for sample normalization. The results are shown as mean ± SD (n = 3), **** p < 0.0001. **I,J)** Transwell migration assays of CRC cells through laminin-111 coated membranes. LS174T cell pools harboring a *LARGE2* knockout (**I**), and HT-29 cells overexpressing *LARGE2* (**J**) were compared to their control cell pools (*: *p* < 0.05; **: p < 0.01; ***: p < 0.001)

Examination of *LARGE2* gene expression data derived from The Cancer Genome Atlas (TCGA, COAD and READ cohorts) [28, 46] revealed markedly elevated *LARGE2* mRNA levels in advanced CRC when compared to normal tissue (Fig. 7A, Additional file 10A). To validate these data on patient-derived, purely epithelial CRC cells, we analyzed *LARGE2* expression in 10 PDTO lines (Additional file 5). Importantly, all analyzed PDTOs were diagnosed as microsatellite stable and did not depend on Wnt-activating growth factors for long-term culture (> 2 month), indicative of cell-intrinsically activated Wnt signaling. 6 PDTOs were established from primary tumor tissue and 4 PDTOs from already liver metastasized CRC (see Additional file 5)**.** As an approximation for non-differentiated human colonic epithelia, *LARGE2* gene expression was determined in three PDO lines. In accordance with TCGA data, *LARGE2* mRNA levels were heterogeneously elevated in PDTOs relative to PDOs (~ 2- to 25-fold), which at least in part might be due to different levels of intrinsic Wnt activity in PDTOs (Fig. 7B)*.* To better address if *LARGE2* was differentially expressed between non-metastatic (M0) or liver-metastatic (M1) primary CRC tissues, we isolated total RNA from FFPE tumor-enriched areas from M0 (*n* = 12) or M1 (n = 12) CRC cases (Additional file 11). Analyses of these samples by TaqMan™ qRT-PCR assays showed a weak but non-significant tendency towards elevated *LARGE2* in M1 CRC (*p* = 0.083) (Fig. 7C). Similarly, RNA-Seq data derived from the TCGA-COAD cohort [46] did not show differential expression of *LARGE2* between M0 (*n* = 365) and M1 (*n* = 71) tumors (Additional file 10), suggesting that the elevated *LARGE2* gene expression level found in CRC cohorts and PDTOs is not affected by metastatic disease progression.

### α-DG O-glycosylation is heterogeneous in a panel of CRC organoids

To address the status of O-glycosylated α-DG in our PDTO CRC panel, we performed immunoblot analysis on WGA-AE purified protein fractions: 7 out of 9 PDTOs showed clear IIh6c4 reactivity, the MW of O-glycosylated α-DG varied between ~ 130–140 kDa (PDTO2) and ~ 250 kDa (PDTO1, PDTO17), and glycosylated α-DG was almost absent or low in PDTO4 and PDTO13, respectively (Fig. 7D**,** Additional file 10 E,F). IHC staining against O-glycosylated α-DG on FFPE CRC tissues from which the PDTOs originated gave concordant results (Fig. 7E**,** Additional file 10G**)**. Elevated *LARGE2* gene expression in PDTO1, 10, and PDTO16 co-occurred with strong intensity and/or higher than “normal” MW versions of α-DG as seen in PDOs (compare to Fig. 4), and PDTO13 showed relatively low levels of *LARGE2* and O-glycosylated α-DG. However, this correlation was not strictly given in all analyzed PDTO lines: PDTO2 and PDTO7 expressed high levels of *LARGE2* but showed α-DG at a comparable MW as seen in non-differentiated PDOs, and PDTO5 and PDTO17 displayed high MW α-DG variants in a background of mildly elevated *LARGE2* levels (Fig. 7B,E**,** Additional file 10F). Therefore, it is likely that also other factors besides LARGE2 affect the complexity/MW of α-DG-attached matriglycan in a subset of CRC. These data suggest that matriglycan formation on α-DG represents a common feature of primary and liver-metastasized CRC cells.

### Wnt/LARGE-dependent O-glycosylation of α-DG affects adhesion to endothelial cells in liver metastatic CRC cells

To address the status of O-glycosylated α-DG in a well-characterized model of liver metastasis, we took advantage of the Fidler cell lines [62] KM12c, that shows a poor capability to colonize the liver after intrasplenic injection, and its liver metastatic derivative KM12-L4a. Here, we observed an enrichment of O-glycosylated α-DG and laminin-binding capacity in KM12-L4a-derived glycoprotein fractions when compared to KM12c (Fig. 7F). This is in accordance with data from Frame M.C. and colleagues, who reported enhanced adhesiveness of KM12-L4a to laminin and other ECM-components relative to KM12c [63]. Importantly, CRISPR/Cas9-mediated KO of *LARGE2* in KM12-L4a cells or acute blockade of Wnt signaling in KM12-L4a-NE, which down-modulated expression of *LARGE2* as early as 24 h, interfered with O-glycosylation of α-DG (Fig. 7G, Additional file 10H,I). Interestingly, the adhesion of CRC cells to the laminin-rich ECM of the liver sinusoid has been demonstrated to play an important role in the formation of metastasis [64]. By performing an endothelial cell adhesion assay [65], we observed that the *LARGE2* KO-mediated loss of laminin-binding α-DG in KM12-L4a cells indeed interfered with their ability to rapidly attach to a confluent layer of HMEC-I immortalized human endothelial cells [66] (Fig. 7H). This might hint to a potential role of O-glycosylated α-DG in the adhesion of circulating CRC cells to the laminin-rich ECM of blood vessels [67]. Future studies should therefore focus on the biological consequence of enhanced laminin-adhesiveness in the context of high Wnt/LARGE2/α-DG signaling for the liver colonization capacity of CRC cells.

### LARGE2/α-DG interferes with CRC cell migration through laminin-coated membranes

Intriguingly, O-glycosylated α-DG was described to interfere with cellular migration and invasiveness in renal and prostate cancer [68]. In accordance with this observation, we observed that *LARGE2* KO-mediated loss of functional α-DG in LS174T and SW620 cells (Fig. 2B,C) augmented in vitro cellular migration through laminin-coated membranes in a transwell migration assay (Fig. 7I and Additional file 10 J). Accordingly, ectopic expression of *LARGE2* in HT-29 cells, which led to the formation of high MW matriglycan on α-DG (Fig. 2E), interfered with cell migration in this setting (Fig. 7J). In agreement with what has been published previously for other tumor entities, our data point to a rather inhibitory role of the laminin adhesion-mediating Wnt/LARGE2/α-DG signaling pathway for cell migration and could therefore act as a limiting factor for the early dissemination of CRC cells from the primary tumor.

## Discussion

Here, we describe a direct link between Wnt signaling and O-linked glycosylation in human colonic epithelial cells and in CRC. Extrinsic and intrinsic modulation of canonical Wnt activity triggered *LARGE2* gene expression, and *LARGE2* affected the abundance, complexity, and laminin-binding capacity of O-glycosylated α-DG. As a component of the intestinal basement membrane (BM), laminin plays an important role in cell polarity, and different types of laminin are expressed in crypt and villus regions [69]. Recent efforts to adapt the human PDO model for growth in chemically defined designer matrices showed that laminin-111 (the major ingredient of Matrigel® Matrix) affects ISC functionality [70]. Our finding that human colonic stem/progenitor cells feature a Wnt/LARGE2-dependent, laminin-binding matriglycan structure on α-DG reveals a potentially relevant molecular trait of hCoSCs in this context. The upward migration of intestinal cells along the vertical crypt axis represents a tightly regulated process that involves epithelial cell-substratum interactions mediated by integrins, cytokines, and the ECM components laminin and collagen IV [71]. Notably, integrins and dystroglycan collaborate in myelin stabilization on peripheral nerves, and they play a redundant role during laminin-dependent epithelial polarization of epiblasts [72, 73]. Since the knock-out of β-integrin alone does not alter epithelial anchorage to the BM in the intestine of adult mice [74], several redundantly acting cell-matrix interactions might assure intestinal epithelial homeostasis. From our results, we suggest that also the Wnt/LARGE2-dependent matriglycan structure on α-DG might contribute to sustain the attachment of intestinal stem/progenitor cells to their laminin-rich BM.

Clinical data show that a large fraction of colorectal tumors with genetic alterations in *APC* express at least one truncated APC variant, which retains one to three 20AAR repeats able to bind and partially control β-catenin [6, 46, 75]. The consequence of this residual APC functionality is a sub-maximal, “just-right” dose of Wnt signaling supposed to optimally facilitate initial cellular transformation: Mice carrying *APC* alleles mutated within the MCR indeed display a more severe pre-malignant polyposis when compared to ApcMin mice expressing a shorter APC variant [76]. In accordance with these findings, we observed a slower proliferation phenotype in CRISPR/Cas9-engineered ADO-ex7 organoid lines when compared to their isogenic ADO-MCR correspondents with lower intrinsic Wnt activity (data not shown). The stronger than “just-right” Wnt dose in pre-malignant ADO-ex7 organoids provoked elevated *LARGE2* gene expression and the formation of “above-normal” molecular weight matriglycan structures on α-DG (> 140 kDa), suggesting that LARGE2/α-DG-mediated laminin adhesion might be oncogenic Wnt dose-dependent at this earliest, pre-malignant stage of CRC. Future studies should therefore clarify the consequences of aberrantly increased cellular adhesion to laminin for the initiation and progression of sporadic adenoma and polyposis.

We noticed that *LARGE2* expression in full-blown CRC and patient-derived PDTOs was strongly elevated when compared to normal tissues and pre-malignant APC-MCR mutant ADOs, which might reflect an upregulation of tumor cell intrinsic Wnt activity during tumor progression. For instance, crosstalk with other oncogenic signaling pathways has been reported to potentiate Wnt signaling in CRC [8, 77]. Alternatively, Wnt signaling-independent transcription factors might collaborate with the β-catenin/TCF7L2 complex in CRC to drive *LARGE2* gene expression and α-DG O-glycosylation beyond the levels found at pre-malignant disease stages. Nevertheless, CRISPR/Cas9-mediated mutagenesis of the endogenous TCF7L2 binding site in intron 1 of *LARGE2* reduced *LARGE2* mRNA levels and the occurrence of high MW matriglycan on α-DG in CRC cells and PDTOs. Together with our analyses on CRC cohorts, which showed a strong correlation of *LARGE2* with the Wnt program, these data suggest that Wnt signaling represents the major driver of LARGE2-dependent matriglycan formation on α-DG in CRC.

While Wnt-driven CRC cells showed enhanced *LARGE2* expression, matriglycan-complexity on α-DG, and affinity towards laminin, studies on prostate and renal cancer provided evidence that O-glycosylated α-DG interferes with cellular migration and invasiveness [21, 68]. We obtained concordant results from in vitro laminin migration assays when modulating the LARGE2/α-DG axis. This points to Wnt/LARGE2/α-DG signaling as a rather tumor-restrictive mechanism at early invasive disease stages, where CRC cells need to overcome the boundaries of the BM to achieve tumor dissemination. Notably, accumulation of nuclear β-catenin in CRC represents a feature of so-called “migrating cancer stem cells” [16, 78]. Furthermore, Wnt signaling in CRC is potentiated by tumor-associated fibroblasts at the tumor stroma interface [9], and patients with tumors characterized by strong expression of the ISC gene signature are prone to suffer from disease recurrence [35]. Since *LARGE2* was strongly associated with these pro-tumorigenic CRC traits according to our analyses, some highly Wnt-driven tumors might develop LARGE2-independent strategies in order to restrict or overcome a potentially migration-inhibitory effect of Wnt/LARGE2/α-DG signaling. As introduced, the physiological process of α-DG O-glycosylation follows a complex order of events, and the action of multiple (> 23) enzymatic activities is necessary to create the glycosylation core on which LARGE1 or LARGE2 synthesize functional matriglycan. While we did not observe downregulation of either of these factors at the transcriptional level when analyzing NGS data derived from TCGA [46] (data not shown), approximately 18% of CRC cases (source: TCGA via cBioPortal, http://www.cbioportal.org) showed missense or truncating mutations in one or more of the respective genes (see Additional file 12). This might prevent or partially restrict matriglycan formation on α-DG in the context of high Wnt activity and could explain the here observed heterogeneous pattern and different MW variants of O-glycosylated α-DG in a panel of PDTOs. How distinct variants of O-glycosylated α-DG differentially affect the invasive behavior of CRC cells in a context of high Wnt activity needs to be further addressed.

Albeit our in vitro data point to a rather anti-cell migratory effect of Wnt/LARGE2/α-DG signaling, adhesion of CRC cells to ECM components of the liver sinusoid plays an important role in the formation of liver metastasis: E.g. therapeutic interference with integrin α2-collagen IV or BCAM-laminin interactions prevented the liver metastatic spread of CRC cells in mouse models [64, 79]. Indeed, we observed that several PDTOs derived from liver metastatic CRC had retained at least a stem/progenitor cell-like or an even elevated complexity of O-glycosylated α-DG and a basal plus membranous localization of this laminin-binding feature. Together with our data on the liver-metastatic KM12c cell derivative KM12-L4a [62], which show that Wnt/LARGE2/α-DG signaling augments CRC cell adhesion to blood vessel endothelial cells, we speculate that CRC cell (sub)-populations highly positive for O-glycosylated α-DG might get either selected for or simply become enriched due to their enhanced blood-vessel adhesiveness during liver colonization. From our in vitro functional data on Wnt/LARGE2/α-DG signaling, we can only speculate that the here identified Wnt/LARGE2/α-DG signaling pathway might play a dual role in CRC progression by limiting early tumor cell migration/dissemination while presumably facilitating liver colonization of intravasated, circulating CRC cells. However, future experiments using appropriate in vivo models that closely recapitulate the complete liver metastatic process are needed to clarify the biological relevance and a potentially dual role of the here identified Wnt/LARGE2/α-DG signaling pathway in metastatic CRC progression.

## Conclusions

The expression of the LARGE2 O-glycosyltransferase encoding gene is regulated by physiologic, extrinsically stimulated Wnt signaling and also by an aberrantly activated Wnt pathway in the context of CRC cell intrinsic, Wnt-activating oncogenic mutations. Since localization of the Wnt transcription factor TCF7L2 to a canonical binding motif within the *LARGE2* gene is necessary for Wnt-mediated *LARGE2* mRNA induction, *LARGE2* can be classified as a direct Wnt target gene.

LARGE2 levels are overall increased in full-blown CRC when compared to non-differentiated, benign colonic epithelial cells. In CRC, the expression of LARGE2 correlates with Wnt signaling intensity, an intestinal stem cell phenotype, and expression of human colonic epithelial stem cell genes in different cohorts of colon cancer patients.

According to our quantitative mass spectrometry data, α-DG presumably represents the main target of LARGE2 in CRC, and our experimental data show that Wnt signaling mediates the functional O-glycosylation of α-DG and, as a biological consequence, the adhesion to laminin in a LARGE2-dependent manner.

LARGE2 gene expression and α-DG O-glycosylation mainly occur in the Wnt-driven stem/progenitor cell compartment at the bottom of human colonic crypts. During CRC-initiation, the length and hence the functionality of truncated APC, which translates to different doses of oncogenic Wnt activity, influences the level of *LARGE2* expression and the O-glycosylation status of α-DG in human adenoma organoids.

Full blown CRC cells and patient-derived tumor organoids show an overall increased but heterogenous occurrence and molecular weight of O-glycosylated α-DG, which at least partially depends on Wnt signaling and LARGE2 functionality.

Functionally, we found that LARGE/α-DG signaling inhibits CRC cell migration but also augments CRC cell adhesion to human blood vessel endothelial cells and might therefore play a context-dependent, dual role in CRC progression.

## Supplementary information

**Additional file 1.** Supplementary materials and methods.

**Additional file 2.** Wnt-signaling in CRC directly stimulates expression of LARGE2. Related to Fig. 1.

**Additional file 3.** Seq analysis after conditional silencing of APC in stably transduced HT-29 cells. Related to Fig. 1C.

**Additional file 4.** LARGE2 gene expression in CRC positively correlates with high Wnt activity and elevated hCoSC gene expression. Related to Fig. 2.

**Additional file 5.** Informations on the patient-derived (tumor) organoid models used in this study.

**Additional file 6.** O-glycosylation of α-DG is mediated by Wnt-signaling through LARGE2. Related to Fig. 3 and Fig. 4.

**Additional file 7.** DAG1 peptide intensities from qLC-MS/MS analysis after overexpression of LARGE2 in HT-29 cells. Related to Fig. 3D.

**Additional file 8.** LARGE2 expression and O-glycosylation of α-DG in human PDOs and intestinal epithelium is enriched in the Wnt-driven stem/progenitor cell compartment. Related to Fig. 5.

**Additional file 9.** LARGE2 expression in mouse adenoma and human engineered adenoma organoid (ADO) pairs carrying different APC truncation mutations. Related to Fig. 5J and Fig. 6.

**Additional file 10.** LARGE2/α-DG expression in primary and liver metastatic CRC. Related to Fig. 7.

**Additional file 11 **Information on the FFPE colorectal cancer tissue samples used for *LARGE2* gene expression analysis. Related to Fig. 7C.

**Additional file 12.** Information on genetic status of several genes within the TCGA CRC cohort from TCGA PanCancer Atlas.

**Additional file 13.** List of used Primers, Oligonucleotides and Plasmids used in this study.

**Additional file 14.** Uncropped images of immunoblot membranes.

## Data Availability

The datasets supporting the conclusions of this article are available in the following repositories: ● RNA-Seq data: Gene Expression Omnibus Accession-No.: **GSE131575, **https://www.ncbi.nlm.nih.gov/geo/query/acc.cgi?acc=GSE131575 ● LC-MS/MS data: PRIDE database, project Accession-No.: **PXD013800, **https://www.ebi.ac.uk/pride/

## Abbreviations

CRC: Colorectal cancer
hCoSC: Human colonic stem cell
ISC: Intestinal stem cell
PDO: Patient-derived organoid
PDTO: Patient-derived tumor organoid
ECM: Extracellular matrix
APC: Adenomatous polyposis coli
α-DG: α-dystroglycan
qChIP: quantitative chromatin immuno-precipitation
WGA: Wheat germ agglutinin
BS: binding site
AE: Affinity enrichment
qRT-PCR: Quantitative real-time PCR
CMS: Consensus molecular subtype
CRIS: Cancer cell intrinsic subtype
OL: Overlay
SD: Standard deviation
SEM: Standard error of the mean
FFPE: Formalin-fixed paraffin-embedded
MCR: Mutation cluster region
4-OHT: 4-hydroxy-tamoxifen
EGF: Epidermal growth factor
DOX: Doxycycline
GW: Gateway
BM: Basement membrane
