# Supplementary material for: Wnt-driven LARGE2 mediates laminin-adhesive O-glycosylation in human colonic epithelial cells and colorectal cancer
Source: Cell Commun Signal. 2020 Jun 25;18:102. doi: 10.1186/s12964-020-00561-6 (PMC7315491; doi:10.1186/s12964-020-00561-6)
Supplement: Supplementary file 2 — Additional file 1. Supplementary materials and methods. [file 12964_2020_561_MOESM1_ESM.docx]

**Additional File 1: Supplementary Methods**

**Clinical Material and FFPE tissue from human CRC and ileum from the ApcMin mouse model**

Biological samples of fresh normal and cancerous tissue specimen were received from individuals undergoing curative colectomy or partial hepatectomy at the Hospital Großhadern, LMU Munich. Samples were taken by a pathologist from residual resected tissue, which was not need for diagnostic purposes, and irreversibly anonymized. This procedure has been classified as uncritical by the ethical committee of the LMU Munich and was specifically approved for our projects (project-No. 591-16-UE and 17-771-UE). Anonymized colorectal cancer specimens (FFPE tissues) from patients that underwent surgical resection at the University of Munich between 1994 and 2017 (LMU, Munich, Germany) were obtained from the archives of the Institute of Pathology. Follow-up data were recorded prospectively by the Munich Cancer Registry (data provided by J. Neumann, LMU, Munich, Germany). Specimens were anonymized, and the study was approved by the institutional ethics committee of the Medical Faculty of the LMU (18-105-UE). FFPE tissue samples from the ApcMin mouse model were provided by the HH lab. Experimentation with these animals was approved by the Government of Upper Bavaria, Germany (AZ 55.2-1-54-2532-4-2014).

**Patient-derived organoid (PDO) culture from human colonic mucosa**

Isolation of crypts from patient material and 3D cultivation was performed as previously described [1]. In brief, cleaned colonic mucosal tissue pieces were incubated in 8 mM EDTA (VWR) for 60 min at 4°C, and vigorous shaking yielded supernatants enriched in colonic crypts. 200-300 crypts were resuspended in 50 µl Matrigel (BD Corning) and plated in 24-well culture dishes. After solidification of matrigel for 15 minutes, crypts were overlaid with WREN medium (Wnt3a, RSPO-3 and Noggin conditioned media (derived from L-WNR cells, CRL-3276, ATCC)) and Advanced DMEM/F12 (ADF, Gibco) 50:50, supplemented with Glutamax, 10 mM HEPES, N-2 [1×], B-27 without retinoic acid [1×] (Invitrogen), 1 mM N-acetylcysteine (Sigma), 50 ng/ml recombinant human epidermal growth factor (EGF, Peprotech), 7.5 μM SB202190 (Sigma) and 10 μM Y27632 (SelleckChem)). Medium was replaced with fresh WREN medium every other day. For serial passaging, PDOs were disaggregated in TripLE select (Gibco) for 5-10 min at 37°C, washed with ADF medium, and re-seeded in Matrigel (BD, growth factor reduced).

**PDTO molecular diagnostics**

Molecular characterization of PDTOs was done at the molecular diagnostics department at the Institute of Pathology of LMU (accredited after DIN 17020 (DAkkS)). In brief, the status of *KRAS*, *NRAS*, *BRAF*, and *PIK3CA* was examined as follows: Partial sequences of *BRAF* exon 15, *KRAS* and *NRAS* exons 2, 3, and 4, and *PIK3CA* exons 9 and 20 were amplified by PCR. Each PCR product was analyzed by established and accredited primer systems via pyrosequencing (Pyromark Q24 Advanced, Qiagen). For analysis of the microsatellite status in PDTOs, microsatellites of the NCI (National Cancer Institute) panel BAT25, BAT26, D5S346 (*APC* locus), D17S250 (*TP53* locus), and D2S123 were amplified by multiplex-PCR. PCR products were electrophoretically separated on the Genetic Analyzer (ABI 3130, Thermo Fisher), and fragment length of PCR amplicons was compared between PDTO and matched normal tissue-derived DNA (fragment length polymorphism analyses). See Additional file 5 for further information.

**Mutation detection assay**

For genotyping of PDOs and engineered ADO-derivatives, genomic DNA was isolated using the GenElute Mammalian Genomic Kit (Sigma-Aldrich, Merck). Primers for PCR amplification using the Q5 HF Master Mix protocol (NEB) are shown in additional file 13. Amplicons were treated as specified in the Alt-R® Genome Editing Detection Kit (IDT) protocol and analysed via agarose gel.

**Plasmids and Cloning**

A pTRIPZ lentiviral vector harboring a shRNA directed against APC (RHS4740-EG324) or a non-silencing shRNA control (RHS4743) (Dharmacon, Thermo Fisher), was used for silencing of APC.

For stable, doxycycline-inducible ectopic expression of genes in PDTOs and CRC cell lines*,* a lentiviral pTZ gateway (gw) vector was generated: pTRIPZ vector was cut with EcoRI-HF and AgeI-HF (NEB) to remove the flanking miR-30 based regions and turboRFP, and de-phosphorylated using Fast-AP (ThermoFisher). A complete Gateway destination vector cassette was then isolated from pLenti CMV-Puro-Dest (Addgene #17452) by enzymatic restriction with MfeI and SgrAI (NEB) and ligated into the linearized TRIPZ backbone using T4 Ligase (NEB) to yield pTZgw. For cloning of *LARGE2* or oncogenic β-catenin (*CTNNB1-S33Y*) encoding cDNAs into pTZgw, the respective ORFs were PCR amplified from human cDNA with attB overhang primers and subsequently re-amplified using attB universal primers via Phusion® High Fidelity Polymerase (NEB). attB-flanked ORFs were cloned into pDONR221 via Gateway® BP Clonase® II (Thermo Fisher) and sequences were confirmed by Sanger Sequencing (M13 fw/rv primer, Eurofins/GATC). Finally, verified cDNAs were transferred from pDONR221 to pTZgw via Gateway® LR Clonase® II (Thermo Fisher).

For CRISPR/Cas9-mediated gene knockout of *LARGE2,* two guide RNAs targeting *LARGE2* in Exon 2 or Exon 3 were designed using the CRISPR design tool crispr.mit.edu (Zhang Lab, MIT). Construction of expression plasmids for *LARGE2* gRNAs was done as described by Ran et al.[2]. In brief, oligo pairs containing the 20-nt guide RNA were annealed and ligated into the lentiviral eCas9 expressing vector pLentiCRISPR-E (gift from Phillip Abbosh, Addgene plasmid #78852). Sequences were verified by Sanger sequencing (Eurofins/GATC, Luxembourg) using the U6seq Fw primer.

FUW-NE and FUW-E vectors were kindly provided by the laboratory of Eduard Batlle at IRB Barcelona. Vector design was as follows: the ERT2 domain from the pCMV-CRE-ERT2[3] was amplified by qRT-PCR and cloned into a modified FUGW lentiviral vector backbone [4]. NTCF (encoding aa 1-90) was then amplified by PCR from the pCDNA3.1-NTCF-NLS [5] and cloned in frame upstream of the ERT2 into the FUW-CMV-ERT2. The final FUW-CMV-NTCF-ERT2 (= NE) lentiviral vector is bicistronic and possesses an IRES sequence downstream of the ERT2 followed by a puromycin resistance cassette for selection of transduced cells.

For the *LARGE2* TCF7L2 reporter construct, a 623 bp DNA fragment containing the TCF7L2 binding motif of *LARGE2* intron 1 was amplified by PCR. The reporter plasmid pBV-Luc (Addgene plasmid #16539) as well as the generated amplicon were digested with EcoRI and HindIII (NEB) and finally assembled via ligation. To create a point-mutated TCF7L2 binding motif, the NEB Q5® Site-Directed Mutagenesis Kit was used as described by the manufacturers’ protocol. Successful mutation was verified by Sanger Sequencing.

**Stable transduction of cell lines and organoids with lentiviral particles**

Lentiviral particles were generated in HEK293T cells from lentiviral vectors encoding for the gene/guide RNA/shRNA of interest and the 2^nd^ generation packaging vectors pMD2.G and psPAX2 (kindly provided by Prof. Andreas Trumpp, DKFZ Heidelberg). Virus containing supernatants were concentrated 20-fold using Lenti-X Concentrator (Clontech, Takara Bio) according to the manufacturer’s protocol. Tumor organoid single cells were plated in suspension on matrigel-coated surfaces prior to infection, and CRC cell lines were directly infected by addition of the concentrated virus particles to the culture medium supplemented with 8 ug/ml Polybrene (Sigma) for 16 hours before-re-embedding cells into 3‑D matrigel. 48 hours later, puromycin selection was started for at least five days (1 µg/mL, Sigma, Merck). Alternatively, virus-containing HEK293T supernatants were passed through 0.45 µm filters (Millipore, MA, USA) and directly used to infect 2-D cultured classical CRC cell lines.

**RNA isolation, cDNA preparation, and qRT-PCR**

Total RNA and complementary DNA (cDNA) was prepared using High Pure RNA Isolation Kit (Roche) and High-Capacity cDNA Reverse Transcription Kit (Applied Biosystems, Thermo Fisher) according to the manufacturer protocols. Real-time PCR was performed using the primaQUANT CYBR qPCR master mix (Steinbrenner Laborsysteme GmbH, Wiesenbach, Germany) on a LightCycler480 (Roche). Relative expression values were normalized to *PPIA* and *B2M* expression and calculated using the ΔΔCt method. Oligonucleotide pairs used for qRT-PCR are listed in additional file 13.

In case of FACS-sorted PTK7 and EPHB2 single cell subpopulations derived from human colon organoids and colonic crypts, respectively, RNA was extracted using Trizol Reagent (Invitrogen, Thermo Fisher) and purified using the RNeasy® MiniKit (Qiagen, Hilden, Germany). qRT-PCR analysis was performed on cDNA using TaqMan^TM^ Gene Expression Assays (Applied Biosystems, see additional file 13) and the primaQUANT qPCR master mix (Steinbrenner Laborsysteme GmbH) following manufacturer’s instructions and using the Roche LightCycler 480-II instrument (Roche).

**Peptide and Protein identification and quantification after LC-MS-MS analysis**

Peptide and protein identification and quantification was performed using MaxQuant [6] (version 1.6.0.16) by searching the tandem MS data against all human canonical and isoform protein sequences as annotated in the Swissprot reference database (42356 entries, downloaded 06.06.2018) using the embedded search engine Andromeda [7]. Carbamidomethylated cysteine was set as fixed modification and oxidation of methionine and N-terminal protein acetylation as variable modification. Trypsin/P was specified as the proteolytic enzyme and up to two missed cleavage sites were allowed. Precursor tolerance was set to 4.5 ppm and fragment ion tolerance to 20 ppm. The minimum peptide length was set to seven and all data were adjusted to 1 % PSM and 1 % protein FDR. Intensity-based absolute quantification (iBAQ) [8] was enabled within MaxQuant. The Perseus software suite [9] (v. 1.6.2.3) was used to filter out contaminants, reverse hits and protein groups, which were only identified by site. As further selection criteria, proteins had to be identified in at least 2 out of 3 replicates in control cells and at higher molecular weight in 3 out of 3 replicates in cells over-expressing *LARGE2*.

**Dual luciferase Reporter Assay**

SW480 cells seeded in 24 well plates were co-transfected with pBV-Luc vector constructs and Renilla luciferase (pRL-TK; Promega, Madison, WI) using Lipofectamine LTX (ThermoFisher Scientific). Reporter activity was measured 36 hours after transfection: Cells were lysed in 50 µl 2x Lysis–Juice and 10 µl cell lysate were mixed with 50 µl Beetle-Juice in a white flat bottom 96-well plate. After measurement on a Berthold Orion II Microplate Luminometer, 50 µl of Renilla-Juice was added to the well and measured as transfection control (Beetle-Juice Luciferase Assay Firefly and Renilla Juice Luciferase Assay, pjk Biotech). Cells were transfected three times, and each sample was measured two times.

1. Jung P, Sato T, Merlos-Suarez A, Barriga FM, Iglesias M, Rossell D, Auer H, Gallardo M, Blasco MA, Sancho E, et al: **Isolation and in vitro expansion of human colonic stem cells.** *Nat Med* 2011, **17:**1225-1227.

2. Ran FA, Hsu PD, Wright J, Agarwala V, Scott DA, Zhang F: **Genome engineering using the CRISPR-Cas9 system.** *Nat Protoc* 2013, **8:**2281-2308.

3. Feil R, Brocard J, Mascrez B, LeMeur M, Metzger D, Chambon P: **Ligand-activated site-specific recombination in mice.** *Proc Natl Acad Sci U S A* 1996, **93:**10887-10890.

4. Lois C, Hong EJ, Pease S, Brown EJ, Baltimore D: **Germline transmission and tissue-specific expression of transgenes delivered by lentiviral vectors.** *Science* 2002, **295:**868-872.

5. van de Wetering M, Sancho E, Verweij C, de Lau W, Oving I, Hurlstone A, van der Horn K, Batlle E, Coudreuse D, Haramis AP, et al: **The beta-catenin/TCF-4 complex imposes a crypt progenitor phenotype on colorectal cancer cells.** *Cell* 2002, **111:**241-250.

6. Cox J, Mann M: **MaxQuant enables high peptide identification rates, individualized p.p.b.-range mass accuracies and proteome-wide protein quantification.** *Nat Biotechnol* 2008, **26:**1367-1372.

7. Cox J, Neuhauser N, Michalski A, Scheltema RA, Olsen JV, Mann M: **Andromeda: a peptide search engine integrated into the MaxQuant environment.** *J Proteome Res* 2011, **10:**1794-1805.

8. Schwanhausser B, Busse D, Li N, Dittmar G, Schuchhardt J, Wolf J, Chen W, Selbach M: **Global quantification of mammalian gene expression control.** *Nature* 2011, **473:**337-342.

9. Tyanova S, Temu T, Sinitcyn P, Carlson A, Hein MY, Geiger T, Mann M, Cox J: **The Perseus computational platform for comprehensive analysis of (prote)omics data.** *Nat Methods* 2016, **13:**731-740.
