# Supplementary material for: Wnt-driven LARGE2 mediates laminin-adhesive O-glycosylation in human colonic epithelial cells and colorectal cancer
Source: Cell Commun Signal. 2020 Jun 25;18:102. doi: 10.1186/s12964-020-00561-6 (PMC7315491; doi:10.1186/s12964-020-00561-6)
Supplement: Supplementary file 3 — Additional file 2. Wnt-signaling in CRC directly stimulates expression of LARGE2. Related to Fig. 1. [file 12964_2020_561_MOESM2_ESM.pdf]

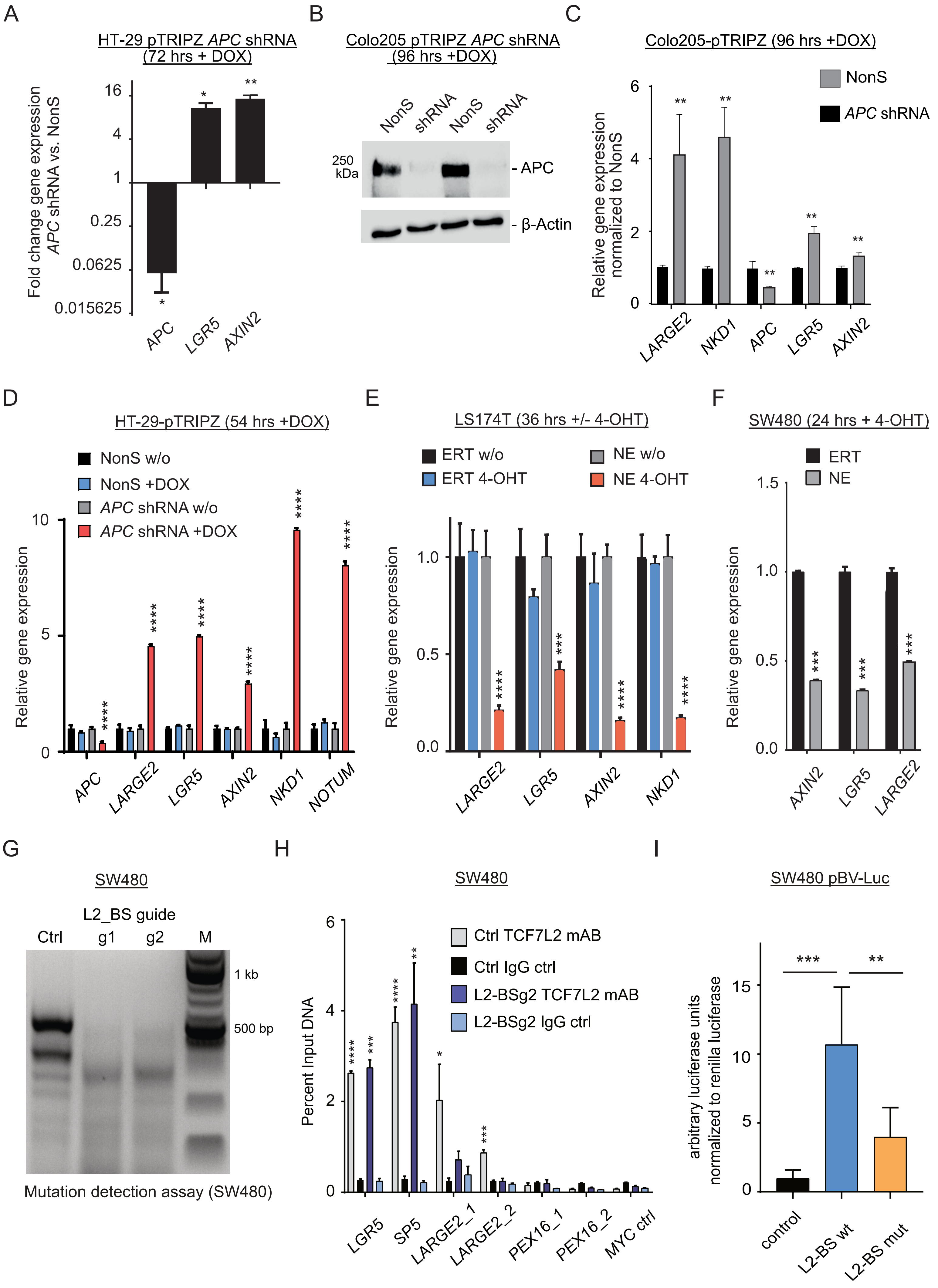

A
HT-29 pTRIPZ APC shRNA (72 hrs + DOX)
Fold change gene expression APC shRNA vs. NonS
16
4
1
0.25
0.0625
0.015625
APC
LGR5
AXIN2
B
Colo205 pTRIPZ APC shRNA (96 hrs +DOX)
NonS
shRNA
NonS
shRNA
250 kDa
- APC
- β-Actin
C
Colo205-pTRIPZ (96 hrs +DOX)
NonS
APC shRNA
Relative gene expression normalized to NonS
LARGE2
NKD1
APC
LGR5
AXIN2
D
HT-29-pTRIPZ (54 hrs +DOX)
NonS w/o
NonS +DOX
APC shRNA w/o
APC shRNA +DOX
Relative gene expression
APC
LARGE2
LGR5
AXIN2
NKD1
NOTUM
E
LS174T (36 hrs +/- 4-OHT)
ERT w/o
ERT 4-OHT
NE w/o
NE 4-OHT
Relative gene expression
LARGE2
LGR5
AXIN2
NKD1
F
SW480 (24 hrs + 4-OHT)
ERT
NE
Relative gene expression
AXIN2
LGR5
LARGE2
G
SW480
L2_BS guide
Ctrl
g1
g2
M
1 kb
500 bp
Mutation detection assay (SW480)
H
SW480
Ctrl TCF7L2 mAB
Ctrl IgG ctrl
L2-BSg2 TCF7L2 mAB
L2-BSg2 IgG ctrl
Percent Input DNA
LGR5
SP5
LARGE2_1
LARGE2_2
PEX16_1
PEX16_2
MYC ctrl
I
SW480 pBV-Luc
arbitrary luciferase units normalized to renilla luciferase
control
L2-BS wt
L2-BS mut

Additional File 2: Wnt-signaling in CRC directly stimulates expression of *LARGE2*

A) qRT-PCR analysis of the indicated genes in HT-29 cells expressing *APC* shRNA, compared to a non-silencing shRNA (NonS) control. Cells were treated with DOX for 72 hours. Shown are mean values ± SD (n=3).* $p < 0.05$, ** $p < 0.01$.

B) Immunoblot analysis of APC and β-actin on WCL from Colo205 cells treated with 500 ng/ml DOX to induce expression of *APC*-targeting shRNA or NonS control.

C, D) qRT-PCR analysis of the indicated genes in Colo205 cells (C) or HT-29 cells (D) expressing *APC*-targeting shRNA. Cells were treated with DOX for 96 and 54 hours, respectively. Mean values ± SD (n=3) are shown. ** $p < 0.01$, **** $p < 0.0001$.

E, F) qRT-PCR analysis of the indicated genes in LS174T (E) or SW480 (F)-NE or -E cells, treated with 400 nM 4-OHT for 24 or 36 hours. Expression was compared to untreated cells (LS174T) or ERT control (SW480). Mean values (n=3) are depicted as mean ± SD., *** $p < 0.001$, **** $p < 0.0001$.

G) Mutation detection assay on genomic DNA from stably transduced SW480 cell pools edited via CRISPR/Cas9 to visualize targeting of the TCF7L2_BS (guides: g1, g2) against control (Ctrl).

H) qChIP analysis on genomic DNA from SW480 cells, wild-type (Ctrl) or mutant for TCF7L2_BS (BSg2). The amount of DNA immunoprecipitated with TCF7L2 antibody or rabbit control IgG in each sample is shown as percentage of chromatin input. Results are shown as mean (n=3) ± SD. * $p < 0.1$; ** $p < 0.05$; *** $p < 0.01$ **** $p < 0.001$.

I) Luciferase reporter assay in SW480 cells, transfected with equimolar amounts of control pBV-Luc vector, the vector containing wt or mutated TCF7L2_BS. A Renilla luciferase vector was used as a transfection control., luciferase activity was measured 36 hrs after transfection. Results are shown as mean (n=3) ± SD. ** $p < 0.01$, *** $p < 0.001$.
