# Supplementary material for: Wnt-driven LARGE2 mediates laminin-adhesive O-glycosylation in human colonic epithelial cells and colorectal cancer
Source: Cell Commun Signal. 2020 Jun 25;18:102. doi: 10.1186/s12964-020-00561-6 (PMC7315491; doi:10.1186/s12964-020-00561-6)
Supplement: Supplementary file 4 — Additional file 3. Seq analysis after conditional silencing of APC in stably transduced HT-29 cells. Related to Fig. 1C. [file 12964_2020_561_MOESM3_ESM.pdf]

Table S1
Page 1

Upregulated genes upon APC silencing in HT29 cells			
ID	Symbol	Log2FoldChange	P-value
ENSG00000140807	NKD1	8.602580674	5.07E-138
ENSG00000180644	PRF1	8.481644466	2.5607E-06
ENSG00000127863	TNFRSF19	8.381922854	8.94884E-06
ENSG00000064300	NGFR	8.321646713	2.32444E-34
ENSG00000124143	ARHGAP40	7.726632799	0.00039067
ENSG00000134258	VTCN1	7.352254249	0.001034598
ENSG00000145832	SLC25A48	7.342235463	0.000578711
ENSG00000212901	KRTAP3-1	7.180547286	0.000121022
ENSG00000185269	NOTUM	7.137887968	1.71427E-80
ENSG00000064692	SNCAIP	7.008623613	3.5781E-12
ENSG00000138316	ADAMTS14	6.999224838	6.61993E-08
ENSG00000158186	MRAS	6.622524955	3.85009E-32
ENSG00000163083	INHBB	6.551174004	3.85781E-07
ENSG00000177494	ZBED2	6.379875635	6.19074E-06
ENSG00000148677	ANKRD1	6.200194548	6.24918E-06
ENSG00000197859	ADAMTSL2	6.175414322	1.32224E-18
ENSG00000197043	ANXA6	6.104449376	2.06357E-21
ENSG00000100593	ISM2	5.343365055	0.001073581
ENSG00000113657	DPYSL3	5.201070519	2.62272E-33
ENSG00000119699	TGFB3	5.039346591	8.62959E-57
ENSG00000123405	NFE2	4.945992665	6.02359E-07
ENSG00000078814	MYH7B	4.884545828	4.09181E-37
ENSG00000064195	DLX3	4.873296004	0.002726643
ENSG00000115596	WNT6	4.865668666	5.41317E-11
ENSG00000155265	GOLGA7B	4.686729917	1.09649E-19
ENSG00000198756	COLGALT2	4.604941281	1.26313E-05
ENSG00000204542	C6orf15	4.440122934	3.0326E-07
ENSG00000110944	IL23A	4.40418074	9.87462E-13
ENSG00000204335	SP5	4.39226625	4.84127E-47
ENSG00000143147	GPR161	4.292967804	3.69664E-07
ENSG00000152229	PSTPIP2	4.282150543	8.3572E-08
ENSG00000156574	NODAL	4.268636635	0.003580412
ENSG00000163864	NMNAT3	4.21450204	0.002788397
ENSG00000125872	LRRN4	4.207032154	0.00033826
ENSG00000165905	LARGE2	4.197424998	5.83497E-45
ENSG00000151790	TDO2	4.16177928	0.000419467
ENSG00000116661	FBXO2	4.05579249	4.80261E-08
ENSG00000162849	KIF26B	4.052303237	3.59831E-13
ENSG00000137285	TUBB2B	4.024336319	3.04917E-06
ENSG00000167552	TUBA1A	3.965094734	7.98863E-28
ENSG00000117115	PADI2	3.940615361	1.57694E-08
ENSG00000112394	SLC16A10	3.916210132	1.40886E-12
ENSG00000144369	FAM171B	3.849232996	0.000164772
ENSG00000165655	ZNF503	3.828123892	7.26616E-15
ENSG00000162946	DISC1	3.755136404	0.009135817
ENSG00000129757	CDKN1C	3.74413193	2.72437E-35
ENSG00000183734	ASCL2	3.722809306	4.91022E-24
ENSG00000102760	RGCC	3.684684589	2.2452E-09

Upregulated genes upon APCsilencing in HT29 cells			
ID	Symbol	Log2FoldChange	P-value
ENSG00000133067	LGR6	3.637082803	3.49356E-06
ENSG00000167749	KLK4	3.605832569	0.002691095
ENSG00000071909	MYO3B	3.597653095	0.002212787
ENSG00000029534	ANK1	3.5578801	0.000173627
ENSG00000111752	PHC1	3.546166766	0.000844523
ENSG00000064201	TSPAN32	3.54102919	6.40192E-07
ENSG00000148468	FAM171A1	3.478764572	0.000384652
ENSG00000198624	CCDC69	3.464214531	3.06696E-09
ENSG00000150687	PRSS23	3.461033515	2.60602E-16
ENSG00000168386	FILIP1L	3.442487888	7.92087E-17
ENSG00000031081	ARHGAP31	3.426654038	0.001629129
ENSG00000165617	DACT1	3.394963271	0.000197769
ENSG00000085741	WNT11	3.32992985	1.22088E-22
ENSG00000163121	NEURL3	3.329803638	0.000236394
ENSG00000147251	DOCK11	3.318623723	8.45706E-05
ENSG00000095637	SORBS1	3.31779005	1.59136E-23
ENSG00000188042	ARL4C	3.316025619	1.46794E-14
ENSG00000175928	LRRN1	3.303454395	5.05584E-11
ENSG00000126778	SIX1	3.301510078	0.002900928
ENSG00000204334	ERICH2	3.247816973	2.84284E-09
ENSG00000128683	GAD1	3.211539088	1.25221E-32
ENSG00000100027	YPEL1	3.18204982	3.41329E-08
ENSG00000184194	GPR173	3.171113232	7.23216E-07
ENSG00000102904	TSNAXIP1	3.144329626	0.005409629
ENSG00000183087	GAS6	3.141731259	2.76034E-09
ENSG00000168874	ATOH8	3.118922279	8.33337E-17
ENSG00000163827	LRRC2	3.092973605	3.04917E-06
ENSG00000168734	PKIG	3.090403583	9.74932E-08
ENSG00000009709	PAX7	3.066677122	0.00143994
ENSG00000084731	KIF3C	3.054514743	2.83417E-16
ENSG00000112655	PTK7	3.042764083	3.65551E-32
ENSG00000124006	OBSL1	3.032608588	1.37319E-08
ENSG00000133216	EPHB2	3.025010537	2.48081E-18
ENSG00000110665	C11orf21	3.017208357	2.07063E-05
ENSG00000204869	IGFL4	3.010297975	1.86142E-16
ENSG00000196628	TCF4	2.992163119	0.00385821
ENSG00000095585	BLNK	2.973615163	7.28056E-07
ENSG00000041982	TNC	2.963289114	9.9644E-15
ENSG00000160360	GPSM1	2.958637938	3.39779E-07
ENSG00000168646	AXIN2	2.931950773	7.96337E-17
ENSG00000204889	KRT40	2.92242472	1.13206E-11
ENSG00000257108	NHLRC4	2.919886562	0.00016286
ENSG00000117122	MFAP2	2.885982335	2.84641E-07
ENSG00000171992	SYNPO	2.877004173	0.00011893
ENSG00000143507	DUSP10	2.868366206	0.000457202
ENSG00000204624	DISP3	2.862716261	0.000296572
ENSG00000184160	ADRA2C	2.852390745	0.002113912
ENSG00000180340	FZD2	2.833675814	2.05044E-16

Upregulated genes upon APCsilencing in HT29 cells			
ID	Symbol	Log2FoldChange	P-value
ENSG00000171243	SOSTDC1	2.810092241	3.19949E-23
ENSG00000117707	PROX1	2.793511856	8.67569E-07
ENSG00000131370	SH3BP5	2.791407584	0.000474945
ENSG00000143434	SEMA6C	2.790280368	0.0001192
ENSG00000101335	MYL9	2.777647736	6.45636E-06
ENSG00000135363	LMO2	2.764288942	0.003483693
ENSG00000115594	IL1R1	2.76248358	0.004557431
ENSG00000182871	COL18A1	2.74042344	2.77553E-17
ENSG00000142619	PADI3	2.737690058	0.004458667
ENSG00000172478	MAB21L4	2.735828425	1.09649E-19
ENSG00000104894	CD37	2.735828155	1.10466E-14
ENSG00000122877	EGR2	2.708316516	0.009307563
ENSG00000019582	CD74	2.705479177	1.52701E-09
ENSG00000091986	CCDC80	2.69957609	0.000619674
ENSG00000108984	MAP2K6	2.688239526	8.05242E-11
ENSG00000139438	FAM222A	2.67794095	1.74346E-23
ENSG00000102575	ACP5	2.672046472	0.000558537
ENSG00000149582	TMEM25	2.659356818	2.17762E-10
ENSG00000103528	SYT17	2.639570346	2.01009E-06
ENSG00000143845	ETNK2	2.625577523	0.003641957
ENSG00000154330	PGM5	2.616409747	0.001003871
ENSG00000183496	MEX3B	2.611344848	1.09334E-10
ENSG00000126016	AMOT	2.602540881	0.006145696
ENSG00000117519	CNN3	2.600542605	1.83357E-11
ENSG00000099953	MMP11	2.59143253	2.98609E-07
ENSG00000108244	KRT23	2.590662348	9.83632E-08
ENSG00000161905	ALOX15	2.587618361	0.000143857
ENSG00000132688	NES	2.584793887	6.90885E-09
ENSG00000101470	TNNC2	2.568570131	0.000432026
ENSG00000163520	FBLN2	2.564563751	0.004836259
ENSG00000183762	KREMEN1	2.549802574	6.70806E-16
ENSG00000166922	SCG5	2.546627877	6.6952E-05
ENSG00000188322	SBK1	2.543519313	1.22283E-13
ENSG00000105419	MEIS3	2.530128635	6.47875E-05
ENSG00000063127	SLC6A16	2.519070448	2.99704E-05
ENSG00000181264	TMEM136	2.504199737	6.85683E-08
ENSG00000213626	LBH	2.502892271	0.000146655
ENSG00000198892	SHISA4	2.501549142	1.07846E-08
ENSG00000165556	CDX2	2.49611389	6.30181E-05
ENSG00000189431	RASSF10	2.491266557	1.22088E-22
ENSG00000211445	GPX3	2.471672355	2.04807E-11
ENSG00000171608	PIK3CD	2.468556942	0.002541775
ENSG00000198933	TBKBP1	2.441221912	6.36429E-05
ENSG00000275896	PRSS2	2.437012862	0.001946202
ENSG00000102547	CAB39L	2.434804025	8.97604E-15
ENSG00000170961	HAS2	2.428882219	1.74429E-12
ENSG00000204950	LRRC10B	2.426935758	2.52172E-05
ENSG00000158806	NPM2	2.420042041	7.50019E-05

Upregulated genes upon APCsilencing in HT29 cells			
ID	Symbol	Log2FoldChange	P-value
ENSG00000132879	FBXO44	2.417558565	1.91066E-07
ENSG00000121653	MAPK8IP1	2.415395945	0.000980426
ENSG00000113758	DBN1	2.411098491	9.59595E-22
ENSG00000157657	ZNF618	2.411002884	2.35336E-09
ENSG00000104081	BMF	2.409065176	7.38962E-10
ENSG00000106038	EVX1	2.404276472	0.005753646
ENSG00000010610	CD4	2.401196396	0.00569163
ENSG00000115112	TFCP2L1	2.39758356	0.000357533
ENSG00000142102	PGGHG	2.380906046	5.78566E-10
ENSG00000100167	SEPT3	2.36881409	1.00614E-06
ENSG00000196132	MYT1	2.361588613	1.37548E-08
ENSG00000198417	MT1F	2.353046319	0.000115224
ENSG00000074219	TEAD2	2.34953883	1.45765E-22
ENSG00000187867	PALM3	2.347467371	4.84483E-05
ENSG00000115507	OTX1	2.346138687	1.58276E-14
ENSG00000153982	GDPD1	2.327746041	0.002212787
ENSG00000160094	ZNF362	2.322680725	9.52382E-07
ENSG00000136425	CIB2	2.320749105	0.000914162
ENSG00000148735	PLEKHS1	2.310077032	0.008428761
ENSG00000134369	NAV1	2.301752102	1.44696E-15
ENSG00000051128	HOMER3	2.282075188	2.45594E-08
ENSG00000151692	RNF144A	2.279505344	0.002603459
ENSG00000130203	APOE	2.275983404	0.009261498
ENSG00000118503	TNFAIP3	2.257376577	0.000315389
ENSG00000205809	KLRC2	2.255839488	3.85097E-06
ENSG00000274180	NATD1	2.255663375	3.21337E-12
ENSG00000124216	SNAI1	2.24513706	1.11904E-08
ENSG00000136490	LIMD2	2.240971365	2.38335E-05
ENSG00000152127	MGAT5	2.225072899	8.48299E-19
ENSG00000071242	RPS6KA2	2.222406752	0.000722787
ENSG00000239521	CASTOR3	2.219900967	1.82282E-10
ENSG00000072195	SPEG	2.218425372	2.26897E-05
ENSG00000124766	SOX4	2.213864709	2.17609E-10
ENSG00000188766	SPRED3	2.206324049	6.79374E-07
ENSG00000131016	AKAP12	2.204023016	0.000188395
ENSG00000063438	AHRR	2.193565987	6.80346E-13
ENSG00000137747	TMPRSS13	2.170903868	2.67881E-06
ENSG00000103196	CRISPLD2	2.162996232	8.22421E-07
ENSG00000204175	GPRIN2	2.159399292	0.000474945
ENSG00000131069	ACSS2	2.152287347	1.49227E-06
ENSG00000163739	CXCL1	2.150080138	3.64392E-05
ENSG00000143365	RORC	2.13972522	0.000720656
ENSG00000253731	PCDHGA6	2.128621178	0.000238468
ENSG00000075618	FSCN1	2.125086149	2.98259E-07
ENSG00000213420	GPC2	2.120444946	9.981E-10
ENSG00000123453	SARDH	2.113210171	0.000417309
ENSG00000184489	PTP4A3	2.103496554	6.87894E-07
ENSG00000035862	TIMP2	2.094519745	0.000296572

Upregulated genes upon APCsilencing in HT29 cells			
ID	Symbol	Log2FoldChange	P-value
ENSG00000012171	SEMA3B	2.088360455	5.68105E-05
ENSG00000140465	CYP1A1	2.080720471	0.002706239
ENSG00000116990	MYCL	2.074421811	4.44412E-10
ENSG00000160469	BRSK1	2.070683199	0.002428153
ENSG00000120885	CLU	2.067374314	6.87953E-06
ENSG00000261934	PCDHGA9	2.056268865	0.000159151
ENSG00000171160	MORN4	2.055049554	0.000261555
ENSG00000139629	GALNT6	2.054137238	1.17241E-08
ENSG00000163545	NUAK2	2.035949841	2.19135E-06
ENSG00000145730	PAM	2.032630978	9.64538E-06
ENSG00000184254	ALDH1A3	2.030614696	8.29048E-07
ENSG00000105767	CADM4	2.015480314	2.01783E-11
ENSG00000089820	ARHGAP4	2.009226032	0.000333205

Downregulated genes upon APCsilencing in HT29 cells			
ID	Symbol	Log2FoldChange	P-value
ENSG00000198088	NUP62CL	-2.006185198	3.4289E-10
ENSG00000115598	IL1RL2	-2.006756954	0.00013471
ENSG00000124107	SLPI	-2.021179432	0.00023257
ENSG00000123095	BHLHE41	-2.031026674	3.5027E-07
ENSG00000123219	CENPK	-2.032463983	0.00072009
ENSG00000205213	LGR4	-2.05210898	1.248E-06
ENSG00000186204	CYP4F12	-2.066840146	0.00434523
ENSG00000152078	TMEM56	-2.067651651	0.00208772
ENSG00000026103	FAS	-2.072048824	6.0608E-05
ENSG00000104537	ANXA13	-2.075074554	1.0916E-06
ENSG00000241635	UGT1A1	-2.079250848	1.1087E-07
ENSG00000114771	AADAC	-2.083743961	0.004211
ENSG00000107159	CA9	-2.084701827	0.00931087
ENSG00000198826	ARHGAP11A	-2.084857509	0.00460269
ENSG00000074410	CA12	-2.101949805	4.4908E-16
ENSG00000173546	CSPG4	-2.109983033	8.0572E-09
ENSG00000162433	AK4	-2.128488392	7.8118E-10
ENSG00000178202	KDELC2	-2.139889178	2.0099E-08
ENSG00000164749	HNF4G	-2.146519604	0.00081192
ENSG00000188993	LRRC66	-2.153379701	0.00026197
ENSG00000215182	MUC5AC	-2.167181039	6.9308E-06
ENSG00000197408	CYP2B6	-2.203119081	0.00020792
ENSG00000242366	UGT1A8	-2.206275932	5.0558E-11
ENSG00000116741	RGS2	-2.215301069	1.4782E-07
ENSG00000171227	TMEM37	-2.229916037	0.00417401
ENSG00000115339	GALNT3	-2.230594674	1.4968E-07
ENSG00000147255	IGSF1	-2.238710903	2.3819E-07
ENSG00000170345	FOS	-2.250174125	1.0629E-13
ENSG00000146477	SLC22A3	-2.254319106	1.6815E-07
ENSG00000000971	CFH	-2.257842011	2.1589E-11
ENSG00000106384	MOGAT3	-2.258620267	3.7738E-08
ENSG00000091409	ITGA6	-2.265340956	3.478E-10
ENSG00000204219	TCEA3	-2.275625015	0.00152552
ENSG00000173467	AGR3	-2.283912139	0.0001692
ENSG00000114248	LRRC31	-2.288037427	0.00122413
ENSG00000120162	MOB3B	-2.288686024	5.4254E-11
ENSG00000102678	FGF9	-2.318020532	6.5843E-05
ENSG00000138193	PLCE1	-2.329875062	8.9488E-06
ENSG00000138587	MNS1	-2.365373138	0.00720807
ENSG00000167653	PSCA	-2.397682868	2.4519E-05
ENSG00000118507	AKAP7	-2.407044271	0.00087028
ENSG00000134827	TCN1	-2.416574065	2.6148E-10
ENSG00000133710	SPINK5	-2.425098096	0.00043503
ENSG00000105388	CEACAM5	-2.467999365	3.4083E-09
ENSG00000100314	CABP7	-2.523521783	2.0009E-05
ENSG00000152952	PLOD2	-2.632761361	5.6929E-19
ENSG00000117983	MUC5B	-2.653682274	0.00616753
ENSG00000141469	SLC14A1	-2.668382689	0.00185439

Downregulated genes upon APCsilencing in HT29 cells			
ID	Symbol	Log2FoldChange	P-value
ENSG00000113722	CDX1	-2.698645974	1.94856E-05
ENSG00000122711	SPINK4	-2.700559365	3.58457E-06
ENSG00000119547	ONECUT2	-2.729203198	0.000153515
ENSG00000113810	SMC4	-2.729782991	0.009922356
ENSG00000089472	HEPH	-2.74079466	1.47848E-08
ENSG00000170381	SEMA3E	-2.835390646	0.00385821
ENSG00000144908	ALDH1L1	-2.835966044	0.000524337
ENSG00000169903	TM4SF4	-2.840114878	3.37385E-19
ENSG00000160182	TFF1	-2.908342635	3.04917E-06
ENSG00000166268	MYRFL	-2.914247816	7.57632E-08
ENSG00000198074	AKR1B10	-2.954632835	9.11808E-06
ENSG00000134193	REG4	-2.993730651	4.82183E-11
ENSG00000166866	MYO1A	-3.02585455	9.63104E-06
ENSG00000134240	HMGCS2	-3.037652515	1.55228E-13
ENSG00000137812	KNL1	-3.051531887	0.004746811
ENSG00000079112	CDH17	-3.055435635	1.31586E-07
ENSG00000163535	SGO2	-3.083993555	0.006059521
ENSG00000100079	LGALS2	-3.161472673	7.78364E-05
ENSG00000172367	PDZD3	-3.205250786	6.62701E-12
ENSG00000109511	ANXA10	-3.205507355	2.01361E-13
ENSG00000086696	HSD17B2	-3.236828004	3.98721E-18
ENSG00000186529	CYP4F3	-3.304182262	0.000248439
ENSG00000145703	IQGAP2	-3.314736975	4.27194E-06
ENSG00000189057	FAM111B	-3.354555064	0.003436299
ENSG00000271447	MMP28	-3.416765137	3.09847E-05
ENSG00000138182	KIF20B	-3.530266008	0.000744477
ENSG00000144674	GOLGA4	-3.53627402	0.003724917
ENSG00000138778	CENPE	-3.550466004	0.000674683
ENSG00000143167	GPA33	-3.560943471	7.3413E-12
ENSG00000139618	BRCA2	-3.564595641	0.001629129
ENSG00000134982	APC	-3.583939355	0.003499252
ENSG00000122483	CCDC18	-3.586350221	0.000395594
ENSG00000163586	FABP1	-4.618716727	0.002603459
ENSG00000142484	TM4SF5	-4.946250774	0.007826525

Additional file 3: RNA Seq analysis after conditional silencing of APC in stably transduced HT-29 cells

HT-29 colorectal cancer cells were stably transduced with lentiviral particles encoding for a doxycyline-inducible shRNA targeting APC or, as a control, a non-silencing (NonS) shRNA. 72 hours after treatment with 500 ng/ml doxycycline, cells were harvested for RNA isolation. Next generation sequencing (RNA Seq) was performed as described in the Materials and Methods section. Shown is the DeSeq2 output file after contrasting biological duplicates of HT-29 *APC* shRNA1 versus HT-29 NonS shRNA samples. Positive fold changes indicate elevated gene expression 72 hours after silencing of *APC*. Significance is indicated as the adjusted p-value. A cut-off was defined as fold-change > 4 and adjusted p-value < 0.01, as described in the main manuscript.
