# Supplementary material for: Wnt-driven LARGE2 mediates laminin-adhesive O-glycosylation in human colonic epithelial cells and colorectal cancer
Source: Cell Commun Signal. 2020 Jun 25;18:102. doi: 10.1186/s12964-020-00561-6 (PMC7315491; doi:10.1186/s12964-020-00561-6)
Supplement: Supplementary file 5 — Additional file 4. LARGE2 gene expression in CRC positively correlates with high Wnt activity and elevated hCoSC gene expression. Related to Fig. 2. [file 12964_2020_561_MOESM4_ESM.pdf]

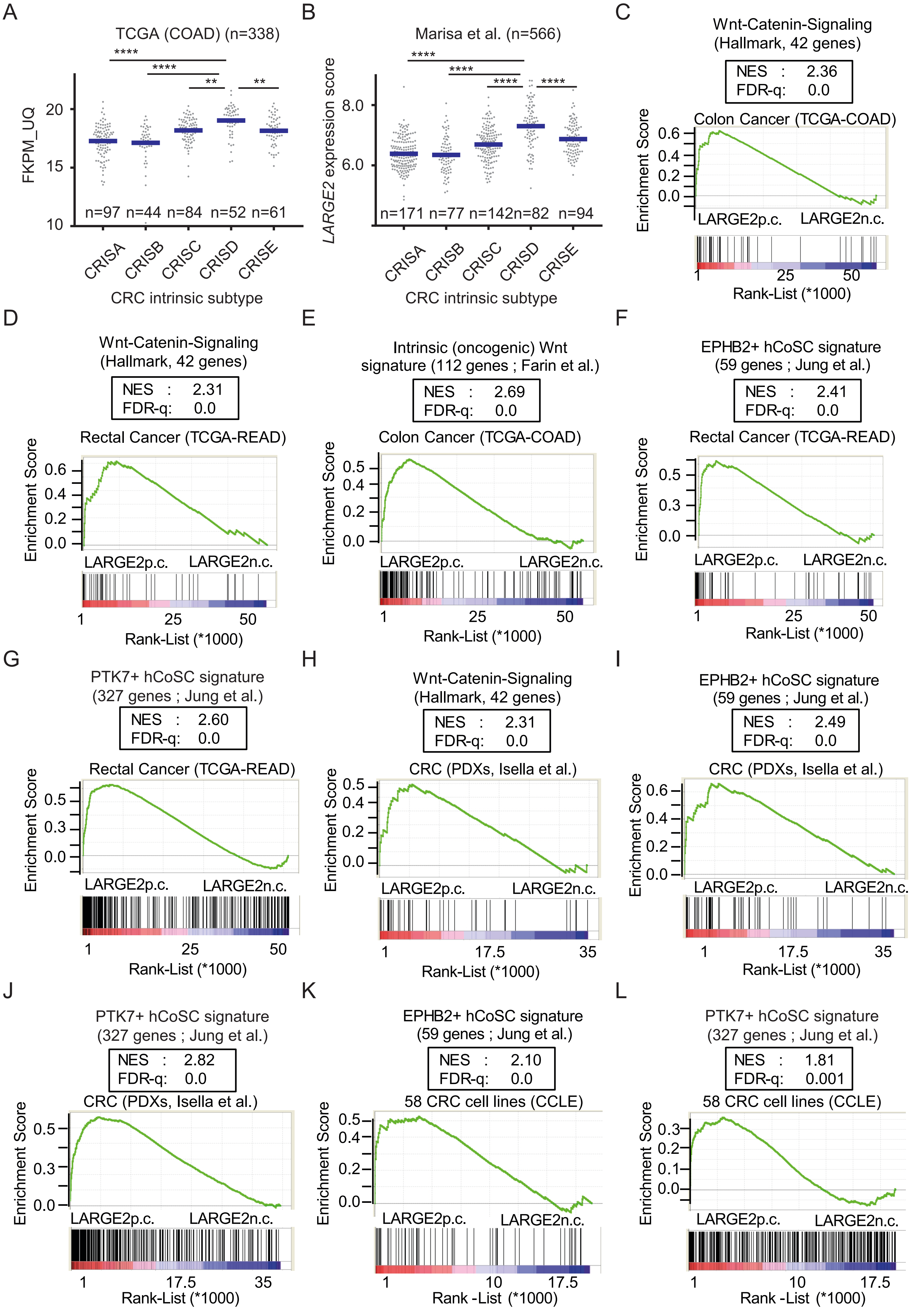
A
TCGA (COAD) (n=338)
****
****
**
**
FKPM_UQ
20
15
10
n=97 n=44 n=84 n=52 n=61
CRISA CRISB CRISC CRISD CRISE
CRC intrinsic subtype
B
Marisa et al. (n=566)
****
****
****
****
LARGE2 expression score
8.0
6.0
n=171 n=77 n=142 n=82 n=94
CRISA CRISB CRISC CRISD CRISE
CRC intrinsic subtype
C
Wnt-Catenin-Signaling (Hallmark, 42 genes)
NES : 2.36
FDR-q: 0.0
Colon Cancer (TCGA-COAD)
Enrichment Score
LARGE2p.c. LARGE2n.c.
Rank-List (*1000)
D
Wnt-Catenin-Signaling (Hallmark, 42 genes)
NES : 2.31
FDR-q: 0.0
Rectal Cancer (TCGA-READ)
Enrichment Score
LARGE2p.c. LARGE2n.c.
Rank-List (*1000)
E
Intrinsic (oncogenic) Wnt signature (112 genes ; Farin et al.)
NES : 2.69
FDR-q: 0.0
Colon Cancer (TCGA-COAD)
Enrichment Score
LARGE2p.c. LARGE2n.c.
Rank-List (*1000)
F
EPHB2+ hCoSC signature (59 genes ; Jung et al.)
NES : 2.41
FDR-q: 0.0
Rectal Cancer (TCGA-READ)
Enrichment Score
LARGE2p.c. LARGE2n.c.
Rank-List (*1000)
G
PTK7+ hCoSC signature (327 genes ; Jung et al.)
NES : 2.60
FDR-q: 0.0
Rectal Cancer (TCGA-READ)
Enrichment Score
LARGE2p.c. LARGE2n.c.
Rank-List (*1000)
H
Wnt-Catenin-Signaling (Hallmark, 42 genes)
NES : 2.31
FDR-q: 0.0
CRC (PDXs, Isella et al.)
Enrichment Score
LARGE2p.c. LARGE2n.c.
Rank-List (*1000)
I
EPHB2+ hCoSC signature (59 genes ; Jung et al.)
NES : 2.49
FDR-q: 0.0
CRC (PDXs, Isella et al.)
Enrichment Score
LARGE2p.c. LARGE2n.c.
Rank-List (*1000)
J
PTK7+ hCoSC signature (327 genes ; Jung et al.)
NES : 2.82
FDR-q: 0.0
CRC (PDXs, Isella et al.)
Enrichment Score
LARGE2p.c. LARGE2n.c.
Rank-List (*1000)
K
EPHB2+ hCoSC signature (59 genes ; Jung et al.)
NES : 2.10
FDR-q: 0.0
58 CRC cell lines (CCLE)
Enrichment Score
LARGE2p.c. LARGE2n.c.
Rank -List (*1000)
L
PTK7+ hCoSC signature (327 genes ; Jung et al.)
NES : 1.81
FDR-q: 0.001
58 CRC cell lines (CCLE)
Enrichment Score
LARGE2p.c. LARGE2n.c.
Rank -List (*1000)

Additional file 4: *LARGE2* gene expression in CRC positively correlates with high Wnt activity and elevated hCoSC gene expression

A,B) *LARGE2* expression in TCGA-COAD (A, n=338) and Marisa et al.(2013) CRC cohort (B, n=566), all stratified into CRC intrinsic subtypes (CRIS) according to Isella et al. (2017). Displayed are the total means, asterisks indicate the significance of differences between individual subtypes compared to CRISD, calculated by one-way ANOVA (** $p < 0.01$, *** $p < 0.001$, **** $p < 0.0001$).

C-G) Gene set enrichment analysis (GSEA) was performed with a signature of *LARGE*-correlated genes derived from the TCGA-COAD and TCGA-READ data sets. Analyses used a Wnt-Catenin-Signaling gene set (Hallmark gene set, Broad Institute), an intrinsic oncogenic Wnt gene set (Michels et al., 2019) (**C-E**), and two sets of genes specifying EPHB2 positive (**F**) or PTK7 positive (**G**) human colonic stem cells (Jung et at., 2011 and 2015).

H-J) GSEA was performed with a LARGE2 gene signature derived from the CRC Pdx dataset, published by Isella et al.(2017), using the Wnt hallmark gene set (**H**) and PTK7 positive and EPHB2 positive human colonic stem cell gene sets (**I,J**).

K,L) GSEA was performed with a LARGE2 gene signature derived from the Cancer cell line encyclopedia (CCLE) CRC cell line (n=58) microarray dataset using the PTK7 positive and EPHB2 positive human colonic stem cell gene sets.
