# Supplementary material for: Wnt-driven LARGE2 mediates laminin-adhesive O-glycosylation in human colonic epithelial cells and colorectal cancer
Source: Cell Commun Signal. 2020 Jun 25;18:102. doi: 10.1186/s12964-020-00561-6 (PMC7315491; doi:10.1186/s12964-020-00561-6)
Supplement: Supplementary file 6 — Additional file 5. Informations on the patient-derived (tumor) organoid models used in this study. [file 12964_2020_561_MOESM5_ESM.pdf]

Table S2_PDO and PDTO panel
relates to Manuscript Figures 4, 5, and 7

Informations on the patient-derived (tumor) organoid models used in this study

Information on primary tissue-derived benign human colorectal organoids

ID	tissue origin	patient background
NM1 /PDO1	colonic mucosa (colon ascendens)	CRC
NM2 /PDO2	rectal mucosa	CRC
NM3 /PDO3	caecal mucosa	CRC
NM4 /PDO4	colonic mucosa (colon ascendens)	CRC

Information on CRC-derived tumor organoids (PDTO: Patient-derived tumor organoid)

ID	tissue origin	location of primary tumor	diagnosis (M0/M1) at surgery (no follow up)	MSI/MSS status	KRAS variant	NRAS variant	BRAF variant	PIK3CA variant
PDTO1	primary tumor	rectum	M1 (liver)	MSS	wt	wt	wt	wt
PDTO2	liver metastasis	colon	M1 (liver)	MSS	wt	wt	wt	wt
PDTO4	primary tumor	caecum	M0	MSS	G12D	wt	wt	wt
PDTO5	liver metastasis	colon	M1 (liver)	MSS	wt	wt	wt	wt
PDTO7	liver metastasis	rectum	M1 (liver)	MSS	wt	wt	wt	wt
PDTO9	primary tumor	colon	M0	MSS	G13D	wt	wt	wt
PDTO10	primary tumor	caecum	M0	MSS	wt	wt	wt	wt
PDTO13	primary tumor	rectum	M0	MSS	wt	G12R	wt	E545A
PDTO16	liver metastasis	colon	M1 (liver)	MSS	wt	wt	wt	wt
PDTO17	primary tumor	colon	M0	MSS	G12R	wt	wt	wt

Additional file 5: Information on the patient-derived (tumor) organoid models used in this study

Patient-derived organoids from human benign large intestinal or CRC tissue were established and molecularly characterized as described in the Materials and Methods section. Numbering is in accordance with our laboratory internal designation of PDTO lines. Missing numbers refer to PDTOs not analyzed in this study and these PDTOs were either microsatellite instable (MSI) or showed a limited ex vivo growth capacity insufficient for the experiments performed in this study.

PDO: patient-derived organoid. PDTO: patient-derived tumor organoid. MSS: microsatellite stable. wt: wild-type. NM: normal mucosa. CRC: colorectal cancer

M0: no distant metastasis detected; M1: positive for liver metastasis
