# Supplementary material for: Wnt-driven LARGE2 mediates laminin-adhesive O-glycosylation in human colonic epithelial cells and colorectal cancer
Source: Cell Commun Signal. 2020 Jun 25;18:102. doi: 10.1186/s12964-020-00561-6 (PMC7315491; doi:10.1186/s12964-020-00561-6)
Supplement: Supplementary file 7 — Additional file 6. O-glycosylation of α-DG is mediated by Wnt-signaling through LARGE2. Related to Fig. 3 and Fig. 4. [file 12964_2020_561_MOESM6_ESM.pdf]

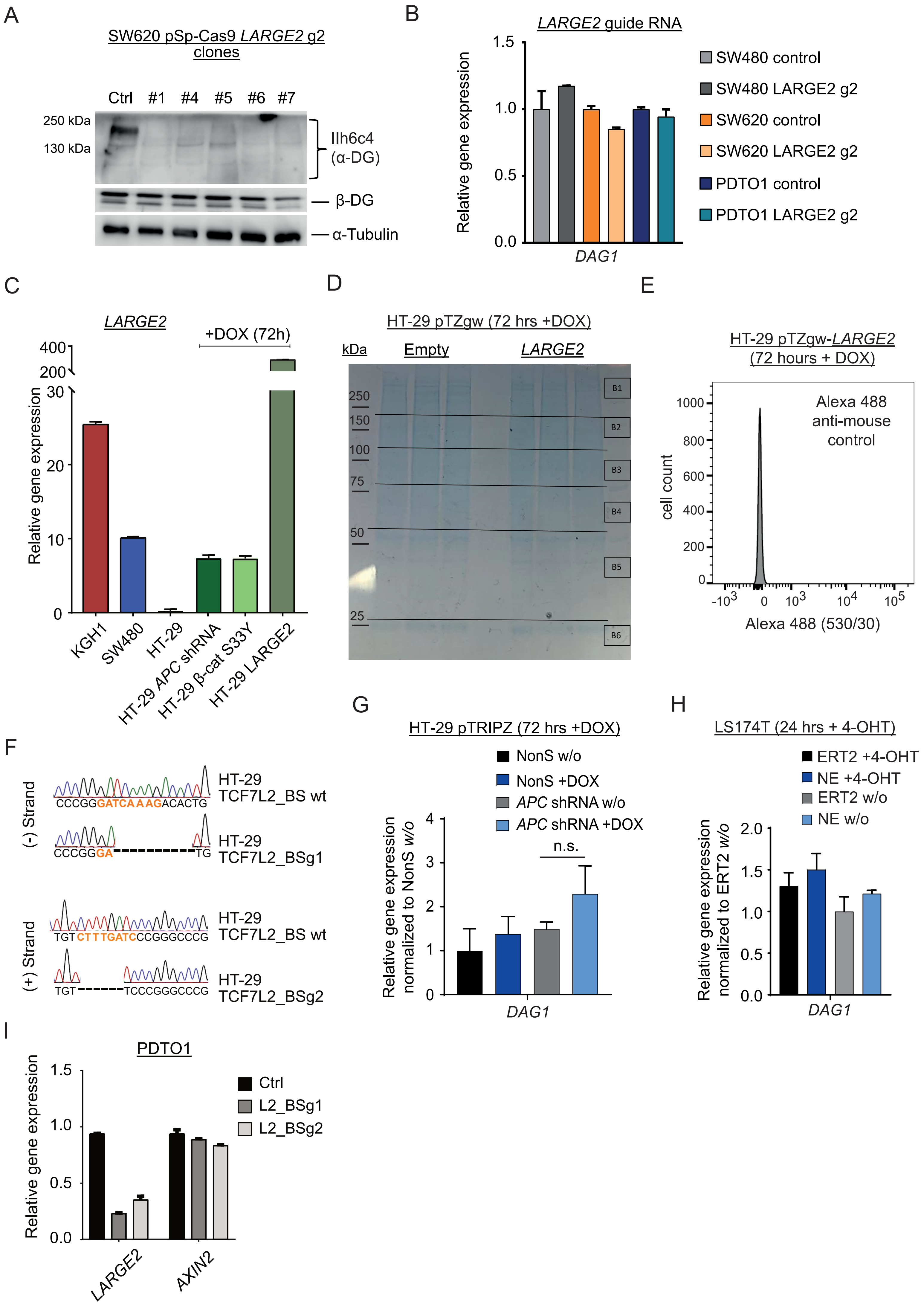
A
SW620 pSp-Cas9 LARGE2 g2 clones
Ctrl #1 #4 #5 #6 #7
250 kDa
130 kDa
IIh6c4 (α-DG)
β-DG
α-Tubulin
B
LARGE2 guide RNA
Relative gene expression
1.5
1.0
0.5
0.0
DAG1
SW480 control
SW480 LARGE2 g2
SW620 control
SW620 LARGE2 g2
PDTO1 control
PDTO1 LARGE2 g2
C
LARGE2
+DOX (72h)
400
200
30
20
10
0
Relative gene expression
KGH1
SW480
HT-29
HT-29 APC shRNA
HT-29 β-cat S33Y
HT-29 LARGE2
D
HT-29 pTZgw (72 hrs +DOX)
kDa
Empty
LARGE2
250
150
100
75
50
25
B1
B2
B3
B4
B5
B6
E
HT-29 pTZgw-LARGE2 (72 hours + DOX)
Alexa 488 anti-mouse control
1000
800
600
400
200
0
cell count
-10^3 0 10^3 10^4 10^5
Alexa 488 (530/30)
F
(-) Strand
CCCGGGATCAAAGACACTG
HT-29 TCF7L2_BS wt
CCCGGGA-----------TG
HT-29 TCF7L2_BSg1
(+) Strand
TGTCTTTGATCCCGGGCCCG
HT-29 TCF7L2_BS wt
TGT-------TCCCGGGCCCG
HT-29 TCF7L2_BSg2
G
HT-29 pTRIPZ (72 hrs +DOX)
NonS w/o
NonS +DOX
APC shRNA w/o
APC shRNA +DOX
n.s.
Relative gene expression normalized to NonS w/o
4
3
2
1
0
DAG1
H
LS174T (24 hrs + 4-OHT)
ERT2 +4-OHT
NE +4-OHT
ERT2 w/o
NE w/o
Relative gene expression normalized to ERT2 w/o
2.0
1.5
1.0
0.5
0.0
DAG1
I
PDTO1
Relative gene expression
1.5
1.0
0.5
0.0
Ctrl
L2_BSg1
L2_BSg2
LARGE2
AXIN2

Additional file 6: O-glycosylation of α-DG is mediated by Wnt-signaling through LARGE2

A) KO of *LARGE2* in CRISPR/Cas9-edited SW620 single clones after transient transfection with a plasmid encoding for Cas9 and guide2. Western Blot analysis of WGA-enriched glycoproteins was used to detect α-DG, WCL were used to detect β-DG and tubulin.

B) qRT-PCR analysis of DAG1 expression after CRISPR/Cas9 mediated KO of LARGE2 in SW480, SW620 cells and PDTO1.

C) Analysis of endogenous *LARGE2* gene expression via qRT-PCR in PDTO1 and CRC cell line SW480, in HT-29 cells wild-type or after conditional expression of APC shRNA or mutant β-catenin (β-cat S33Y), in comparison to the DOX-induced ectopic expression of *LARGE2* in stably transduced HT-29 cells (HT-29 LARGE2). Results are shown as mean ± SD from technical duplicates.

D) Cell lysates from HT-29 cells over-expressing *LARGE2* or control cells (+DOX, 72 hrs) were enriched for glycoproteins, separated via SDS-PAGE and divided into 6 fractions B1-B6 according to their molecular weight for qLC-MS/MS analysis as indicated.

E) Secondary antibody control staining (Alexa 488 IgG) from single cell FACS analysis of HT-29 cells (see Fig. 3F).

F) Sanger sequencing on genomic DNA from HT-29 single cell-derived cell lines, comparing the wild-type (wt) TCF7L2_BS and CRISPR/Cas9-mutated binding sites (BS) after editing with guideRNA1 (BSg1) and guideRNA2 (BSg2). TCF7L2 binding motif is highlighted in orange.

G,H) qRT-PCR analysis of DAG1 expression upon *APC* silencing for 72 hrs in HT-29 (**G**) and Wnt pathway down-modulation in LS174T (**H**) cells. Gene expression is relative to NonS without DOX or ERT2 control without 4-OHT treatment, respectively. Results are shown as mean ± SD (n=3). n.s. not significant.

I) qRT-PCR analysis of indicated genes in PDTO1 after CRISPR/Cas9-mediated mutation of the TCF7L2_BS in intron 1 of *LARGE2* using two different guide RNAs (L2_BSg1 and LS_BSg2). Results are shown as mean ± SD from three technical replicates.
