# Supplementary material for: Wnt-driven LARGE2 mediates laminin-adhesive O-glycosylation in human colonic epithelial cells and colorectal cancer
Source: Cell Commun Signal. 2020 Jun 25;18:102. doi: 10.1186/s12964-020-00561-6 (PMC7315491; doi:10.1186/s12964-020-00561-6)
Supplement: Supplementary file 8 — Additional file 7. DAG1 peptide intensities from qLC-MS/MS analysis after overexpression of LARGE2 in HT-29 cells. Related to Fig. 3D. [file 12964_2020_561_MOESM7_ESM.pdf]

Table S3_DAG1 peptide intensities from qLC-MS/MS analysis page 1

Gene		Position in protein		Intensity Control R1					
Gene names	Sequence	Start position	End position	R1 B01	R1 B02	R1 B03	R1 B04	R1 B05	R1 B06
DAG1	SFRVTIPTDLIASSGDIIK	77	95	0	0	0	0	0	0
DAG1	VTIPTDLIASSGDIIK	80	95	0	0	0	0	134930000	0
DAG1	EALPSWLHWDSQSHTLEGLPLDTDKGVHYISVSATR	102	137	0	0	0	0	0	0
DAG1	GVHYISVSATR	127	137	0	0	0	0	0	0
DAG1	SFSEVELHNMK	216	226	0	0	0	0	91186000	0
DAG1	LVPVVNNR	227	234	0	0	0	0	249210000	0
DAG1	LFDMSAFMAGPGNAK	235	249	0	0	0	0	24582000	0
DAG1	KVVENGALLSWK	250	261	0	0	0	0	43770000	0
DAG1	VVENGALLSWK	251	261	0	0	0	0	37121000	0
DAG1	LGCSLNQNSVPDIHGVEAPAR	262	282	0	0	0	0	62746000	0
DAG1	EGAMSAQLGYPVVGWHIANK	283	302	0	0	0	0	0	0
DAG1	GGEPNQRPELK	490	500	0	0	639550000	0	0	0
DAG1	NHIDRVDAWVGTYFEVK	501	517	0	0	21699000	0	0	0
DAG1	IPSDTFYDHEDTTTDK	518	533	0	0	50938000	0	0	0
DAG1	IPSDTFYDHEDTTTDKLK	518	535	0	0	1055200000	0	0	0
DAG1	LREQQLVGEK	540	549	0	0	480800000	0	0	0
DAG1	EQQLVGEK	542	549	0	0	99329000	0	0	0
DAG1	SWVQFNSNSQLMYGLPDSSHVGK	550	572	0	0	0	0	0	0
DAG1	HEYFMHATDK	573	582	0	0	130450000	0	0	0
DAG1	GGLSAVDAFEIHVHR	583	597	0	0	150300000	0	0	0
DAG1	AKFVGDPALVLNDIHKK	610	626	0	0	46269000	0	0	0
DAG1	FVGDPALVLNDIHK	612	625	0	0	283600000	0	0	0
DAG1	FVGDPALVLNDIHKK	612	626	0	0	1125400000	0	0	0
DAG1	KLAFAFGDR	632	640	0	0	34634000	0	0	0
DAG1	EQIAGLSR	672	679	0	0	0	0	0	761830000
DAG1	EQIAGLSRR	672	680	0	0	0	0	0	27380000
DAG1	RIAEDDGKPR	680	689	0	0	0	0	0	0
DAG1	IAEDDGKPR	681	689	0	0	0	0	0	18456000
DAG1	IAEDDGKPRPAFSNALEPDFK	681	701	0	0	0	0	0	211430000
DAG1	PAFSNALEPDFK	690	701	0	0	0	0	0	46628000
DAG1	PAFSNALEPDFKATSITVTGSGSCR	690	714	0	0	0	0	0	0
DAG1	ATSITVTGSGSCR	702	714	0	0	0	0	0	52295000
DAG1	HLQFIPVVPPR	715	725	0	0	0	0	53559000	380280000
DAG1	LTLEDQATFIK	783	793	0	0	0	0	0	0
DAG1	LTLEDQATFIKK	783	794	0	0	0	0	0	0
DAG1	GVPIIFADELDDSKPPPSSSMPLILQEEK	795	823	0	0	0	0	0	0
DAG1	SPPPYVPP	888	895	0	0	0	0	0	0

Table S3_DAG1 peptide intensities from qLC-MS/MS analysis page 2

Intensity Control R2						Intensity Control R3					
R2 B01	R2 B02	R2 B03	R2 B04	R2 B05	R2 B06	R3 B01	R3 B02	R3 B03	R3 B04	R3 B05	R3 B06
0	0	0	0	67543000	0	0	0	0	0	0	0
0	0	0	0	124130000	0	0	0	0	0	69380000	0
0	0	0	0	0	0	0	0	0	0	0	0
0	0	0	0	0	0	0	0	0	0	0	0
0	0	0	0	54770000	0	0	0	0	0	81254000	0
0	0	0	0	162710000	0	0	0	0	0	0	0
0	0	0	0	31315000	0	0	0	0	0	34035000	0
0	0	0	0	37846000	0	0	0	0	0	0	0
0	0	0	0	28483000	0	0	0	0	0	36326000	0
0	0	0	0	75891000	0	0	0	0	0	53752000	0
0	0	0	0	63425000	0	0	0	0	0	47445000	0
0	0	426040000	0	0	0	0	0	194990000	0	0	0
0	0	0	0	0	0	0	0	0	0	0	0
0	0	0	0	0	0	0	0	0	0	0	0
0	0	725920000	0	0	0	0	0	75216000	0	0	0
0	0	1065900000	0	0	0	0	0	880270000	0	0	0
0	0	524820000	0	0	0	0	0	341560000	0	0	0
0	0	0	0	0	0	0	0	0	0	0	0
0	0	102910000	0	0	0	0	0	28255000	0	0	0
0	0	104700000	0	0	0	0	0	88633000	0	0	0
0	0	0	0	0	0	0	0	35621000	0	0	0
0	0	172500000	0	0	0	0	0	28053000	0	0	0
0	0	820130000	0	0	0	0	0	499660000	0	0	0
0	0	27004000	0	0	0	0	0	22188000	0	0	0
0	0	0	0	0	698560000	0	0	0	0	135170000	775650000
0	0	0	0	0	64810000	0	0	0	0	24871000	66832000
0	0	0	0	0	15461000	0	0	0	0	5180600	28959000
0	0	0	0	0	9765100	0	0	0	0	9033100	0
0	0	0	0	0	131710000	0	0	0	0	0	159140000
0	0	0	0	0	0	0	0	0	0	0	74520000
0	0	0	0	0	0	0	0	0	0	0	11455000
0	0	0	0	0	57443000	0	0	0	0	0	40957000
0	0	0	0	0	514710000	0	0	0	0	15329000	427900000
0	0	0	0	0	0	0	0	0	0	27900000	0
0	0	0	0	0	0	0	0	0	0	29932000	0
0	0	0	0	0	0	0	0	0	0	0	0
0	0	0	0	0	0	0	0	0	0	17323000	0

Table S3_DAG1 peptide intensities from qLC-MS/MS analysis page 3

Intensity Overexpression R1						Intensity Overexpression R2					
R1 B01	R1 B02	R1 B03	R1 B04	R1 B05	R1 B06	R2 B01	R2 B02	R2 B03	R2 B04	R2 B05	R2 B06
0	0	0	0	0	0	0	0	0	0	0	0
19650000	0	0	0	190520000	0	0	0	0	0	183560000	0
0	0	0	0	72782000	0	0	0	0	0	40611000	0
0	0	0	0	27744000	0	0	0	0	0	34353000	0
0	0	0	0	102620000	0	0	0	0	0	116920000	0
0	0	0	0	227450000	0	0	0	0	0	254750000	0
0	0	0	0	0	0	0	0	0	0	23164000	0
0	0	0	0	47439000	0	0	0	0	0	36253000	0
0	0	0	0	43908000	0	0	0	0	0	0	0
0	0	0	0	66938000	0	0	0	0	0	69356000	0
0	0	0	0	83155000	0	0	0	0	0	45680000	0
181060000	37636000	31365000	0	0	0	208030000	111390000	75678000	0	0	0
55251000	0	0	0	0	0	44415000	0	0	0	0	0
0	0	0	0	0	0	0	0	0	0	0	0
309980000	72887000	105410000	0	0	0	438780000	168930000	120460000	0	0	0
789760000	0	185700000	0	0	0	0	85173000	96946000	0	0	0
0	0	0	0	0	0	266060000	0	0	0	0	0
0	0	0	0	0	0	31057000	0	0	0	0	0
37972000	0	0	0	0	0	0	0	0	0	0	0
232430000	71824000	0	0	0	0	163180000	56940000	0	0	0	0
0	0	0	0	0	0	0	0	0	0	0	0
88292000	41928000	0	0	0	0	121520000	44813000	0	0	0	0
520620000	133780000	84959000	0	0	0	645390000	140810000	125280000	0	0	0
36662000	0	0	0	0	0	42714000	0	0	0	0	0
0	0	0	0	54512000	542650000	0	0	0	0	0	569170000
0	0	0	0	0	22438000	0	0	0	0	0	0
0	0	0	0	0	0	0	0	0	0	0	0
0	0	0	0	0	20310000	0	0	0	0	0	11980000
0	0	0	0	0	110890000	0	0	0	0	0	0
0	0	0	0	0	0	0	0	0	0	0	0
0	0	0	0	0	0	0	0	0	0	0	0
0	0	0	0	0	56749000	0	0	0	0	0	0
0	0	0	0	13374000	477030000	0	0	0	0	83386000	52673000
0	0	0	0	0	0	0	0	0	0	30950000	0
0	0	0	0	0	0	0	0	0	0	0	0
0	0	0	0	0	0	0	0	0	0	34372000	0
0	0	0	0	0	0	0	0	0	0	0	0

Table S3_DAG1 peptide intensities from qLC-MS/MS analysis page 4

Intensity Overexpression R3									
R3 B01	R3 B02	R3 B03	R3 B04	R3 B05	R3 B06	MS/MS Count	Length	Mass	Score
0	0	0	0	0	0	1	19	2032.1205	62.739
0	0	0	0	194140000	0	10	16	1641.9189	120.53
0	0	0	0	106200000	0	4	36	4044.997	115.71
0	0	0	0	34669000	0	3	11	1188.6251	69.346
0	0	0	0	103670000	0	15	11	1319.618	214.83
0	0	0	0	298390000	0	7	8	909.5396	106.76
0	0	0	0	31684000	0	6	15	1555.7163	162.36
0	0	0	0	61818000	0	7	12	1342.7609	155.99
0	0	0	0	55041000	0	6	11	1214.6659	140.75
0	0	0	0	99791000	0	7	21	2233.091	218.72
0	0	0	0	113090000	0	8	20	2127.0571	66.777
234330000	43821000	34875000	0	0	0	22	11	1223.6258	183.57
49008000	0	0	0	0	0	3	17	2048.0116	161.99
0	0	0	0	0	0	1	16	1883.8061	126.63
521720000	51600000	174130000	0	0	0	28	18	2124.9851	362.15
553020000	106450000	263690000	0	0	0	16	10	1198.667	96.009
50746000	0	0	0	0	0	7	8	929.48181	164.68
46458000	0	0	0	0	0	2	23	2580.2067	95.138
63504000	0	0	0	0	0	10	10	1277.5499	111.86
145700000	57324000	0	0	0	0	14	15	1606.8216	125.53
0	0	0	0	0	0	2	17	1864.0571	93.269
146090000	40618000	0	0	0	0	19	14	1536.83	184.03
547180000	123550000	172100000	0	0	0	27	15	1664.925	184.31
52787000	0	0	0	0	0	6	9	1023.5502	110.76
0	0	0	0	97950000	734310000	9	8	872.47158	105.25
0	0	0	0	0	37622000	7	9	1028.5727	126.41
0	0	0	0	0	0	2	10	1155.5996	68.355
0	0	0	0	0	25157000	6	9	999.49852	106.04
0	0	0	0	0	159930000	12	21	2316.1386	172.61
0	0	0	0	0	45000000	5	12	1334.6507	199.03
0	0	0	0	0	0	1	25	2612.2541	101.3
0	0	0	0	0	76633000	7	13	1295.614	141.1
0	0	0	0	58290000	686600000	61	11	1301.7608	166.3
0	0	0	0	26808000	0	3	11	1277.6867	125.56
0	0	0	0	0	0	1	12	1405.7817	73.082
0	0	0	0	0	0	1	29	3151.5999	110.03
0	0	0	0	0	0	1	8	852.43815	115.83

Additional file 7: DAG1 peptide intensities from qLC-MS/MS analysis after overexpression of LARGE2 in HT-29 cells

Shown are the DAG1 peptide sequences, as detected by LC-MS/MS (see methods section for details). DAG1 peptide processing is defined as follows: position 1-29: signal peptide; 30-653: α-Dystroglycan; 654-895: β-Dystroglycan. B1 – B6: pre-fractionated samples according to their molecular weight, as described in Figure 3D. Control: Dox-treated HT-29 control cells, Overexpression: Dox-induced LARGE2 expression in HT-29 cells.
