# Supplementary material for: Wnt-driven LARGE2 mediates laminin-adhesive O-glycosylation in human colonic epithelial cells and colorectal cancer
Source: Cell Commun Signal. 2020 Jun 25;18:102. doi: 10.1186/s12964-020-00561-6 (PMC7315491; doi:10.1186/s12964-020-00561-6)
Supplement: Supplementary file 9 — Additional file 8. LARGE2 expression and O-glycosylation of α-DG in human PDOs and intestinal epithelium is enriched in the Wnt-driven stem/progenitor cell compartment. Related to Fig. 5. [file 12964_2020_561_MOESM8_ESM.pdf]

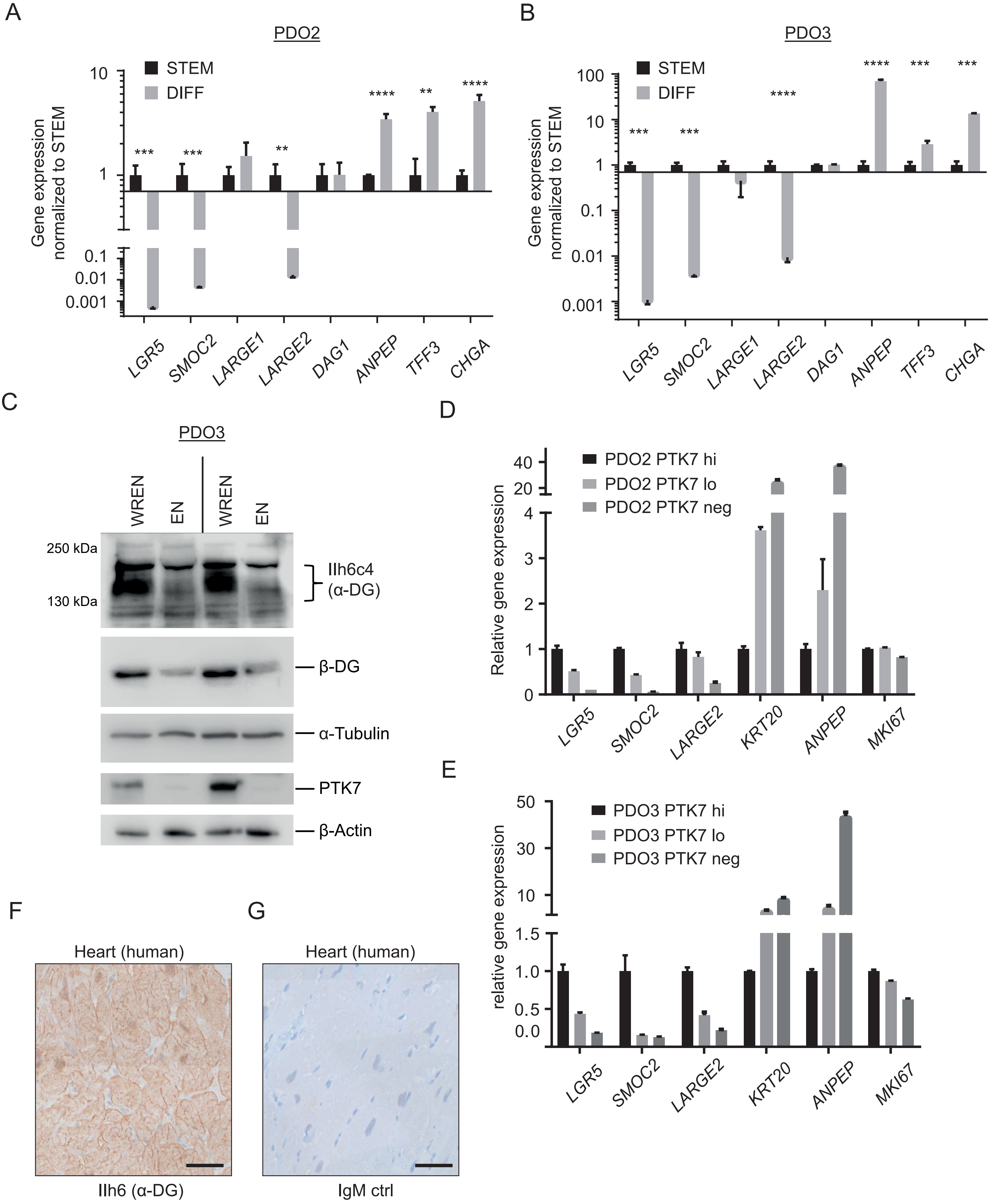

A
PDO2
STEM
DIFF
Gene expression normalized to STEM
10
1
0.1
0.01
0.001
***
***
**
****
**
****
LGR5
SMOC2
LARGE1
LARGE2
DAG1
ANPEP
TFF3
CHGA
B
PDO3
STEM
DIFF
Gene expression normalized to STEM
100
10
1
0.1
0.01
0.001
***
***
****
****
***
***
LGR5
SMOC2
LARGE1
LARGE2
DAG1
ANPEP
TFF3
CHGA
C
PDO3
WREN
EN
WREN
EN
250 kDa
130 kDa
IIh6c4 (α-DG)
β-DG
α-Tubulin
PTK7
β-Actin
D
PDO2 PTK7 hi
PDO2 PTK7 lo
PDO2 PTK7 neg
Relative gene expression
40
20
4
3
2
1
0
LGR5
SMOC2
LARGE2
KRT20
ANPEP
MKI67
E
PDO3 PTK7 hi
PDO3 PTK7 lo
PDO3 PTK7 neg
relative gene expression
50
30
10
1.5
1.0
0.5
0.0
LGR5
SMOC2
LARGE2
KRT20
ANPEP
MKI67
F
Heart (human)
IIh6 (α-DG)
G
Heart (human)
IgM ctrl

Additional file 8: *LARGE2* expression and O-glycosylation of α-DG in human PDOs and intestinal epithelium is enriched in the Wnt-driven stem/progenitor cell compartment

A,B) qRT-PCR analysis of indicated genes in additional colonic organoid lines derived from normal mucosa (PDO2 and 3) and originating from different patients. PDOs were maintained in STEM (WREN) or DIFF (EN) medium for 72 hours. Results are shown as mean ± SD from three technical replicates. ** $p < 0.01$, *** $p < 0.001$, **** $p < 0.0001$.

C) Immunoblot analysis of WGA-AE purified O-glycosylated α-DG from PDO3, maintained for 72 hours in either WREN or EN media. WCL was used to detect β-DG and tubulin.

D,E) mRNA expression analysis via qRT-PCR of PDO2 and PDO3 single cell fractions after FACS sorting according to the surface abundance of the human CoSC marker PTK7. **Neg**: PTK7 negative fraction, **lo**: PTK7 low, **hi**: PTK7 high. Results are shown as mean ± SD from technical duplicates.

F,G) Immunohistochemistry staining on FFPE sections of human heart muscle tissue, using IIh6 antibody for α-DG staining (**F**) and IgM as negative control (**G**). Scale bar indicates 50 μm.
