# Supplementary material for: Wnt-driven LARGE2 mediates laminin-adhesive O-glycosylation in human colonic epithelial cells and colorectal cancer
Source: Cell Commun Signal. 2020 Jun 25;18:102. doi: 10.1186/s12964-020-00561-6 (PMC7315491; doi:10.1186/s12964-020-00561-6)
Supplement: Supplementary file 10 — Additional file 9. LARGE2 expression in mouse adenoma and human engineered adenoma organoid (ADO) pairs carrying different APC truncation mutations. Related to Fig. 5J and Fig. 6. [file 12964_2020_561_MOESM9_ESM.pdf]

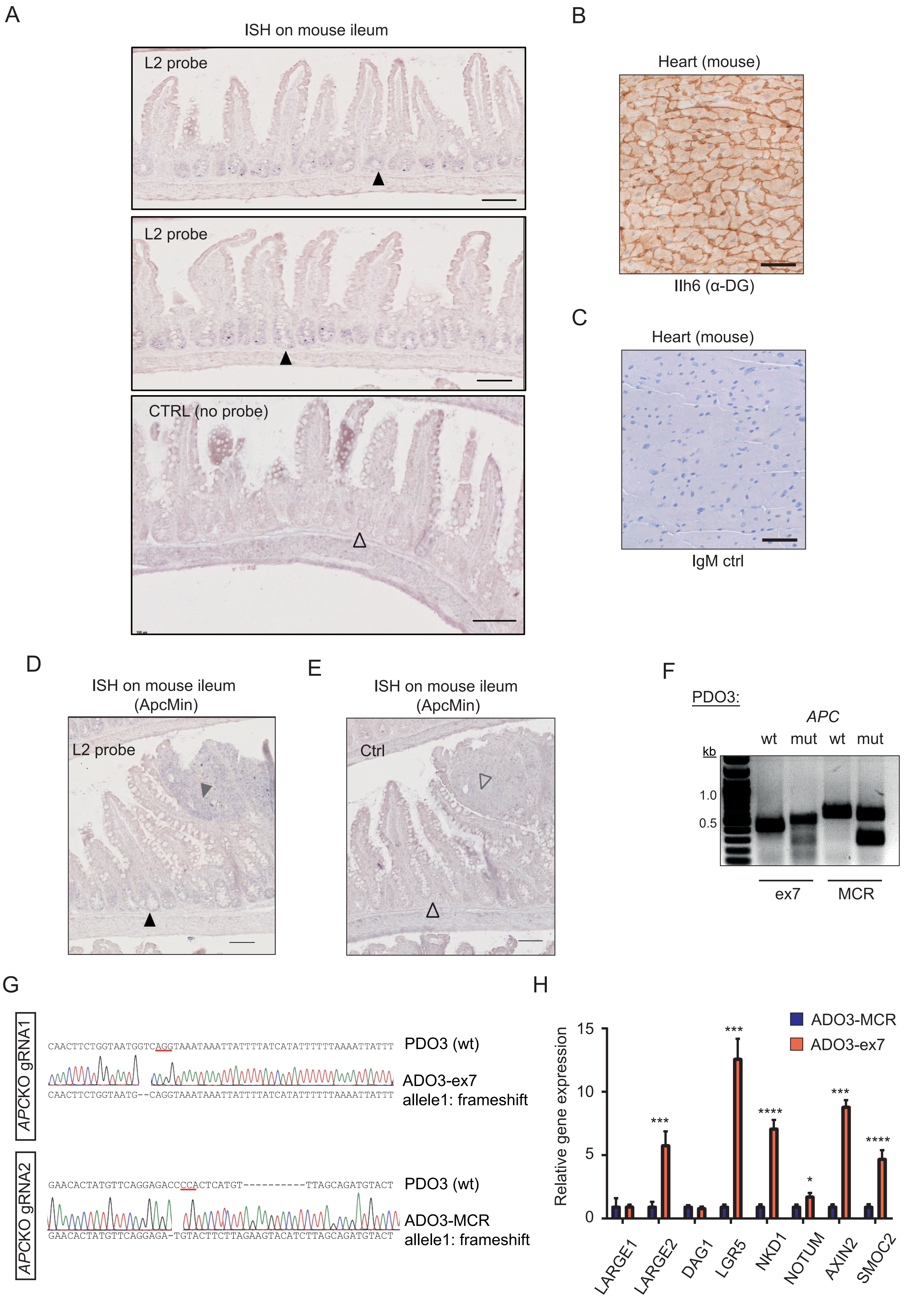
A
ISH on mouse ileum
L2 probe
L2 probe
CTRL (no probe)
B
Heart (mouse)
IIh6 (α-DG)
C
Heart (mouse)
IgM ctrl
D
ISH on mouse ileum
(ApcMin)
L2 probe
E
ISH on mouse ileum
(ApcMin)
Ctrl
F
PDO3:
APC
wt mut wt mut
kb
1.0
0.5
ex7
MCR
G
APCKO gRNA1
CAACTTCTGGTAATGGTCAGGTAAATAAATTATTTTATCATATTTTTTAAAATTATTT
PDO3 (wt)
CAACTTCTGGTAATG--CAGGTAAATAAATTATTTTATCATATTTTTTAAAATTATTT
ADO3-ex7
allele1: frameshift
APCKO gRNA2
GAACACTATGTTCAGGAGACCCCACTCATGT----------TTAGCAGATGTACT
PDO3 (wt)
GAACACTATGTTCAGGAGA-TGTACTTCTTAGAAGTACATCTTAGCAGATGTACT
ADO3-MCR
allele1: frameshift
H
ADO3-MCR
ADO3-ex7
Relative gene expression
15
10
5
0
***
***
****
*
***
****
LARGE1
LARGE2
DAG1
LGR5
NKD1
NOTUM
AXIN2
SMOC2

Additional file 9: *LARGE2* expression in mouse adenoma, and human engineered adenoma organoid (ADO) pairs carrying different *APC* truncation mutations

A) In-situ hybridization on mouse ileum tissue from FFPE sections with a *Large2* specific probe (top and middle) or without probe (control, bottom). Scale bars indicate 100 µm.

B,C) Immunohistochemistry staining on FFPE sections of mouse heart muscle tissue, using IIh6 antibody as positive control (**B**) for α-DG staining and IgM as negative control (**C**). Scale bar indicates 50 µm.

D,E) In-situ hybridization of a *Large2* probe (**D**) and control probe (**E**) on mouse ileum from APCmin mice. Black arrowhead indicates specific staining at the crypt base; grey arrowhead indicates staining in Wnt-driven adenoma. Clear arrowheads show lack of staining in the control experiment.

F,G) Mutation detection assay on genomic DNA derived from additional human normal mucosa organoids (wt) or *APC* mutated adenoma organoids PDO3 (mut, ex7, MCR) (**F**) and sanger sequencing profile (**G**) of both *APC* mutated organoid lines (Top: ADO3-ex7, Bottom: ADO3-MCR). Shown are deletions and frameshifts indicative of CRISPR/Cas9-mediated mutations in *APC* in PDO3.

H) qRT-PCR analysis of the indicated genes in adenomas engineered by CRISPR/Cas9-mediated targeting of *APC* in normal mucosa (ADO3-MCR versus ADO3-ex7). Shown is the mean ± SD (n=3). * $p < 0.01$, *** $p < 0.001$, **** $p < 0.0001$
