# Supplementary material for: Wnt-driven LARGE2 mediates laminin-adhesive O-glycosylation in human colonic epithelial cells and colorectal cancer
Source: Cell Commun Signal. 2020 Jun 25;18:102. doi: 10.1186/s12964-020-00561-6 (PMC7315491; doi:10.1186/s12964-020-00561-6)
Supplement: Supplementary file 11 — Additional file 10. LARGE2/α-DG expression in primary and liver metastatic CRC. Related to Fig. 7. [file 12964_2020_561_MOESM10_ESM.pdf]

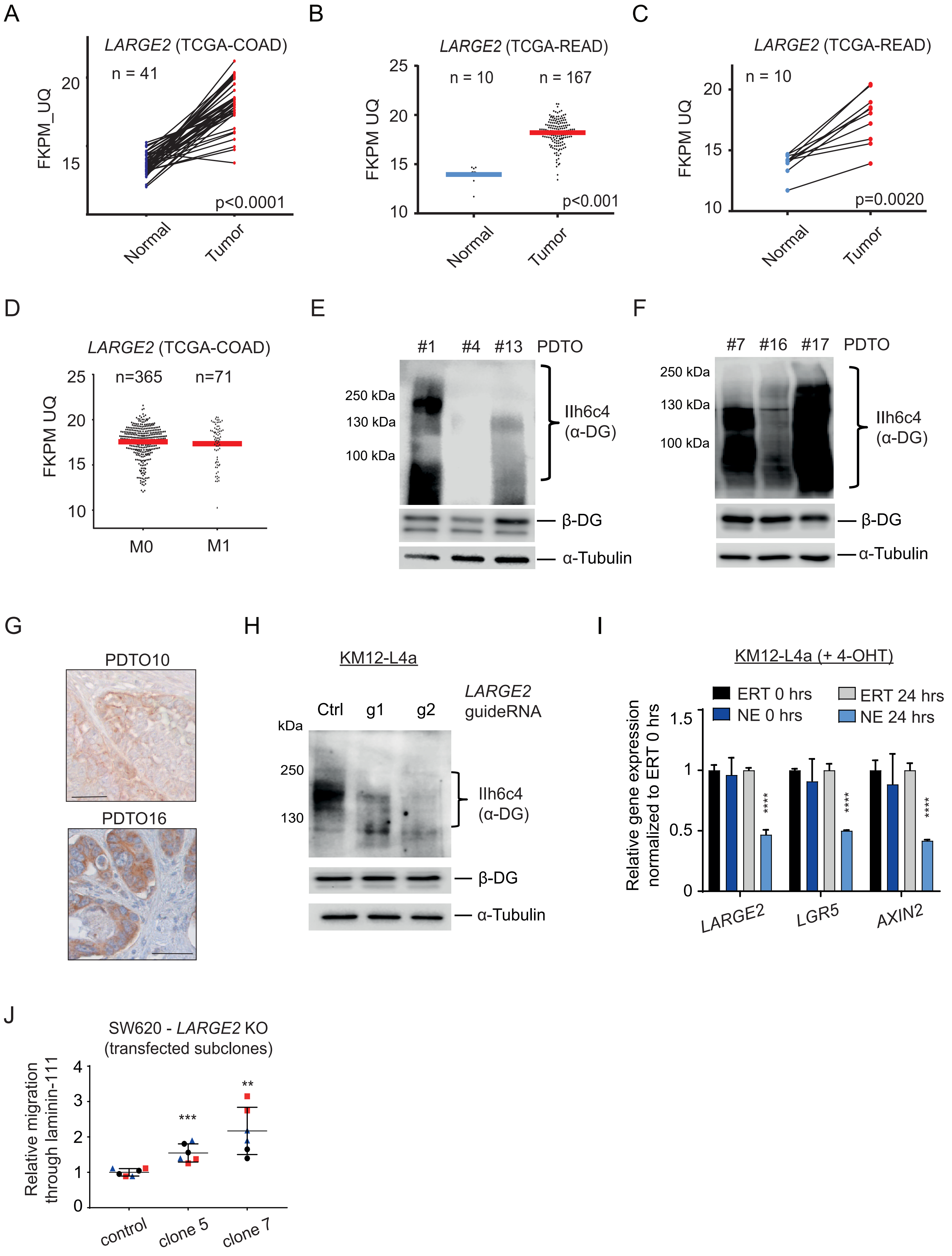
A
LARGE2 (TCGA-COAD)
n = 41
FKPM_UQ
20
15
p<0.0001
Normal
Tumor
B
LARGE2 (TCGA-READ)
n = 10
n = 167
FKPM UQ
25
20
15
10
p<0.001
Normal
Tumor
C
LARGE2 (TCGA-READ)
n = 10
FKPM UQ
20
15
10
p=0.0020
Normal
Tumor
D
LARGE2 (TCGA-COAD)
n=365
n=71
FKPM UQ
25
20
15
10
M0
M1
E
#1 #4 #13 PDTO
250 kDa
130 kDa
100 kDa
IIh6c4 (α-DG)
β-DG
α-Tubulin
F
#7 #16 #17 PDTO
250 kDa
130 kDa
100 kDa
IIh6c4 (α-DG)
β-DG
α-Tubulin
G
PDTO10
PDTO16
H
KM12-L4a
Ctrl g1 g2
LARGE2 guideRNA
kDa
250
130
IIh6c4 (α-DG)
β-DG
α-Tubulin
I
KM12-L4a (+ 4-OHT)
ERT 0 hrs
NE 0 hrs
ERT 24 hrs
NE 24 hrs
Relative gene expression normalized to ERT 0 hrs
1.5
1
0.5
0
****
LARGE2
LGR5
AXIN2
J
SW620 - LARGE2 KO (transfected subclones)
Relative migration through laminin-111
4
3
2
1
0
***
**
control
clone 5
clone 7

Additional File 10: *LARGE2/*α-DG expression in primary and liver metastatic CRC

A-C) *LARGE2* expression analysis on TCGA datasets**:** (**A**) comparison of COAD patient-matched normal human mucosa samples and tumor tissues, (**B**) READ normal tissue and tumor tissue and (**C**) patient-matched READ tumor and normal samples.

D) *LARGE2* gene expression analysis on the TCGA-COAD dataset, comparing tissue samples M0 (not metastasic) and M1 (metastatic) CRC cases.

E,F) Immunoblot analysis of O-glycosylated α-DG in WGA-AE purified glycoprotein fractions from a panel of primary and liver metastatic CRC PDTOs. WCL was used to detect β-DG and tubulin.

G) Immunohistochemistry analysis of O-glycosylated α-DG (IIh6 antibody) on formalin-fixed paraffin-embedded patient derived tissue samples (matching primary tissues to PDTOs 10 and 16).

H) Immunoblot analysis of glycosylated α-DG after KO of *LARGE2* via CRISPR/Cas9 in KM12-L4a cells using two different guideRNAs. WCL was used to detect β-DG and tubulin.

I) Gene expression analysis of indicated genes via qRT-PCR from KM12-L4a-NE or -E cells after treatment with 400 nM 4-OHT for 24 hours. Expression was compared to –ERT cells, or to –NE cells without 4-OHT treatment. Mean values ± SD (n=3) are provided. **** $p < 0.0001$

J) Transwell migration assays of CRC cells through laminin-111 coated membranes. SW620 cell lines harboring a *LARGE2* knockout were compared to control cells. ** $p < 0.01$, *** $p < 0.001$.
