# Supplementary material for: Wnt-driven LARGE2 mediates laminin-adhesive O-glycosylation in human colonic epithelial cells and colorectal cancer
Source: Cell Commun Signal. 2020 Jun 25;18:102. doi: 10.1186/s12964-020-00561-6 (PMC7315491; doi:10.1186/s12964-020-00561-6)
Supplement: Supplementary file 12 — Additional file 11 Information on the FFPE colorectal cancer tissue samples used for LARGE2 gene expression analysis. Related to Fig. 7C. [file 12964_2020_561_MOESM11_ESM.pdf]

Table S4_M0 M1 RNA cohort
related to Figure 7C

Liver metastatic CRC	Gender (m=0/f=1)	Localisation 1 Primarius	Localisation 2 Primarius	Localisation 3 Primarius	Grading	pT-Stadium	pN-Status	pM-Status	R-Status	L-Status	V-Status
**M1 (ID)**											
41C	0	1	67	1	2	3	1	X	0		
17C	0	2	8	1	2	3	0	0	0		
11C	1	0	4	1	2	4	0	0	0	0	0
28C	1	1	67	1	2	3	2		0	1	0
40C	0	2	9	1	2	3	1	0	0	1	
58C	0	2	8	1	2	3	2	1	0	1	0
21C	0	2	9	1	2	3	1	0	1		
79C	0	2	7	1	2	3	1	1			
10C	0	0	6	1	2	4	0	X			
48C	0	0	0	0	3	4	2	X			
39C	1	0	5	1	3	3	1	X			
34C	0	2	9	1	2	2	0	0	0	0	1
**Non-metastatic CRC**	**Gender (m=0/f=1)**	**Localisation 1 Primarius**	**Localisation 2 Primarius**	**Localisation 3 Primarius**	**Grading**	**pT-Stadium**	**pN-Status**	**pM-Status**	**R-Status**	**L-Status**	**V-Status**
**M0 (ID)**											
34B	0	0	6	1	2	2	1	0	0		
79B	0	2	9	1	2	3	0	0	1		
10B	0	0	6	1	2	4	0	0			
11B	1	0	6	1	2	4	0	0			
71B	0	0	0	0	2	3	2	0			
47B	1	0	1	0	3	3	1	0	0		
58B	0	1	67	1	2	3	1	0			
40B	0	2	7	1	2	3	0	0	0	0	0
19B	0	0	0	0	2	3	0	0	0	0	0
69B	0	0	1	0	2	3	1	0			
21B	0	2	9	1	2	3	0	0	0		
48B	0	0	1	0	3	4	0	0			

Localisation 1 Primarius (0=Colon; 1= rectosigmoid junction, 2=Rectum)
Localisation 2 Primarius (Cecum = 0; Appendix = 00; Colon ascendens = 1; right Colonflexure = 2; Colon transversum = 3;
left Colonflexure = 4; Colon descendens = 5; Sigma = 6; 67 = rectosigmoid junction;
upper third of the rectum = 7; middle third of the rectum = 8; lower third of the rectum = 9)
Localisation 3 Primarius (0=right; 1=left)
pT-Stadium postoperative (p) (T1= 1; T2= 2; T3 = 3; T4 = 4)
pN-Status postoperative (p) (=LN-Status) (N0=no LN diseased ; N1=1; N2=2)
pM-Status postoperative (p) (M0 = no distant metastasis; M1 = metastasis to distant organs)
R-Status postoperative (R0 = 0, R1 = 1; R2 = 2)
L-Status postoperative (invasion into lymphatic vessels =1; no invasion = 0)
V-Status postoperative (Invasion into vein =1; no invasion = 0)

Additional file 11: Information on the FFPE colorectal cancer tissue samples LARGE2 gene expression analysis

Clinical information of the FFPE colorectal cancer tissue samples, M1 (liver metastatic) or M0 (non-metastatic), which were used for RNA isolation and Taqman-based qRT-PCR analyses of *LARGE2* gene expression (see Figure 7C).
