# Supplementary material for: Wnt-driven LARGE2 mediates laminin-adhesive O-glycosylation in human colonic epithelial cells and colorectal cancer
Source: Cell Commun Signal. 2020 Jun 25;18:102. doi: 10.1186/s12964-020-00561-6 (PMC7315491; doi:10.1186/s12964-020-00561-6)
Supplement: Supplementary file 13 — Additional file 12. Information on genetic status of several genes within the TCGA CRC cohort from TCGA PanCancer Atlas. [file 12964_2020_561_MOESM12_ESM.pdf]

Gene Name	Missense Mutation	Truncating Mutation	Fusion	Amplification	Deep Deletion
DAG1	1.52%	0.38%			0.38%
POMT1	2.47%	0.19%		0.19%	
POMT2	2.47%	0.19%			
POMGNT1	1.71%	0.38%			
POMGNT2	2.47%	0.19%		0.19%	
MGAT5B	2.66%	0.95%		0.76%	
B3GALNT2	0.38%			0.57%	
POMK	0.76%	0.38%	0.19%	3.61%	0.19%
FKTN	1.14%	0.76%			0.19%
FKRP	0.57%				
RXYLT1	0.38%	0.57%			
B4GAT1	1.33%				0.19%
LARGE1	3.04%	0.38%		0.19%	0.76%
LARGE2	1.71%	0.76%		0.19%	
CHST10	3.04%	0.57%			
DPM1	0.57%	0.19%		5.70%	
DPM2	0.19%				
DPM3				0.57%	
DOLK	1.33%	0.19%			
CRPPA	1.52%	0.57%		0.38%	
HK1	2.47%	0.19%	0.19%	0.38%	0.19%
MPI	0.76%	0.38%			
PMM2	0.38%	0.19%			0.19%
GMPPB	0.38%			0.38%	
FURIN	2.47%	0.76%		0.38%	0.19%

TCGA PanCaner Atlas **n=526**

Summary for Colorectal (TCGA PanCan)
Gene altered in 32.7% of 526 cases

Alteration	Frequency
Mutation	18.44% (97 cases)
Fusion	0.38% (2 cases)
Amplification	11.03% (58 cases)
Deep Deletion	1.71% (9 cases)
Multiple Alterations	1.14% (6 cases)

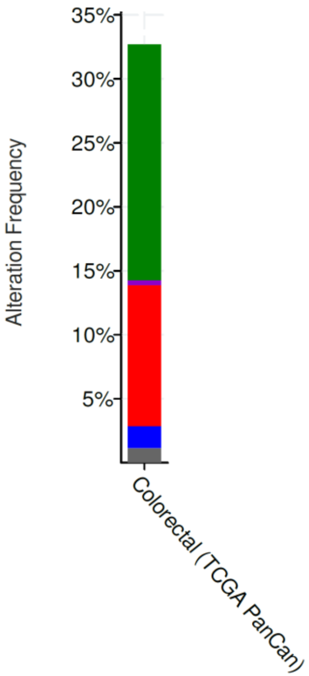

Additional file 12: Mutational status of genes involved in α-DG glycosylation in CRC

We analyzed the genetic status of the indicated genes within the TCGA cohort of 526 colorectal cancer cases (PanCancer Atlas) accessible via the cBioPortal (www.cbioportal.org). Percentages give the frequency of a given genetic alteration found in 526 CRC cases. Note that *LARGE1* and *LARGE2* plus 23 factors known to be involved in α-DG glycosylation were each affected in only a small (3 percent and less) fraction of tumors. Overall, approximately 18.5% of CRC cases harbour mutations in one or several of these genes, and 1.7% of patients were affected by deep deletions on one of the analyzed gene loci.
