# Supplementary material for: Wnt-driven LARGE2 mediates laminin-adhesive O-glycosylation in human colonic epithelial cells and colorectal cancer
Source: Cell Commun Signal. 2020 Jun 25;18:102. doi: 10.1186/s12964-020-00561-6 (PMC7315491; doi:10.1186/s12964-020-00561-6)
Supplement: Supplementary file 14 — Additional file 13. List of used Primers, Oligonucleotides and Plasmids used in this study. [file 12964_2020_561_MOESM13_ESM.pdf]

Additional File 13: Used Primer, Oligonucleotides and Plasmids in this study

Primer

qPCR	Forward	Reverse
**APC**	TCCTAGAGGAGCCAAGCCAT	TTGAGTTTCGGCCAGGAGAC
**AXIN2**	ATGGGATGATCTGTTGCAGAGGGA	TGTCATTTCCACGAAAGCACAGCG
**LARGE2**	GGCCCTCACGCCTTACGT	GTAGCGCAGGGTCTCGAATG
**LGR5**	AATCCCCTGCCCAGTCTC	CCCTTGGGAATGTATGTCAGA
**ASCL2**	GTGAAGCTGGTGAACTTGGGC	CAGCGTCTCCACCTTGCTCA
**NKD1**	ACTTTCGGCTGGAAGTGGC	GTGACCTTGCCGTTGTTGTC
**SP5**	CAGGCCTTTCTCCAGGACC	GGTGTAAGGGGAAGCTCGTG
**NOTUM**	CTGGTGGAACGCAAACATGG	CACACGGTCCACATTCAGGA
**CTNNB**	AGCTGACCAGCTCTCTCTTCA	CCAATATCAAGTCCAAGATCAGC
**WNT11**	TCTTTGGGGTGGCACTTCTC	CTGCCGAGTTCACTTGACGA
**CA12**	GACCTGCACAGTGACATCCT	GCTGGCGTCAGGATAAAGGT
**LGALS4**	GGCTCAACGTGGGAATGTCT	ACCGCGGATTGAAGTGGAAG
**SMOC2**	AACTGGTCTGCGTGCATAA	CTCTCAGACTGTCCTCCAAATG
**ANPEP**	TTGACCGCTCCGAGGTCTAT	CTCGCTGTACTGGTCCATCA
**TFF3**	GCAGGAAGCAGAATGCACCT	AGCTGAGATGAACAGTGCCT
**CHGA**	CAAGACCTCGCTCTCCAAGG	CTCTTCCACCGCCTCTTTCA
**KRT20**	ACTCCAGACACACGGTGAACT	TGAAGTCCTCAGCAGCCAG
**LARGE1**	TTCTGGAACAGAGCTGCACG	TCCTCGGCCTCCCTCATAAT
**PPIA**	AGCATGTGGTGTTTGGCAAA	TCGAGTTGTCCACAGTCAGC
**B2m**	TCCATCCGACATTGAAGTTG	ACACGGCAGGCATACTCAT
**B3GNT1_1**	CCGCATGCCCATGAACAAAA	CGCAGGCCTGGCTGATTC
**POMGNT2**	CAAACCTGTGCTGGCTGC	GGTGAAAGCCTGAAGCCTGG
**B3GALNT2**	CCTGCAGATCAGTTGGCCT	CAGCCCCGATGAAGTGTCTT
**RXYLT1**	CTTTGCCCCCTACAGCCTC	GACTGTTCTTGCGGCGATTT
**POMT1**	CCCCGAAGTAAGAACTCCGT	GCTCAGTAACCCCATCCCAG
**POMT2**	TCAGACAAAGTGTGCCTCCG	CTCATCCCAACAGATGTGCG
**FKTN**	TGGCCCTTTTAACGCTGACA	TTAACTGCACGCCACTGTGT
**FKRP**	CCATTGCTCCAAGATGGCG	AGTAGAAACAGGGGGCTTGC
**KI67**	ACGAGACGCCTGGTTACTATC	GCTCATCAATAACAGACCCATTTAC
**DAG1**	GGAGAACCCAACCAGCGCCCAGAGC	CGGGTGATATTCTGCAGGGTGATGG

Taqman probe assay Ids (Applied Biosystems, Thermo Fisher Scientific)

**LGR5**	Hs00173664_m1
**SMOC2**	Hs00405777_m1
**LARGE2**	Hs00293342_m1
**B2M**	Hs99999907_m1
**KRT20**	Hs00300643_m1
**MKI67**	Hs00606991_m1
**ANPEP**	Hs00174265_m1
**PPIA**	Hs99999904_m1

guideRNAs for gene knockouts

**LARGE2 guide1**	GGCCCCCGGGTCTCCGTCGA
**LARGE2 guide2**	GAAGAGCATGGACTTCACCA
**TCF7L2_BS guide1**	GCCCGGGATCAAAGACACTG
**TCF7L2_BS guide2**	CCCGCAGTGTCTTTGATCCC

**ChIP qPCR**		
**LARGE2_intron1_1**	GAAGGCTTCAGGTGCTCCG	GGGATCAAAGACACTGCGGG
**LARGE2_intron1_2**	CAGAGCAGGTGTCCCAGAC	GGATCAAAGACACTGCGGGA
**LARGE2 upstream_1**	ACAGCGATCACTACCCTGACT	CCCTGACCTTTGATCAGTTCC
**LARGE2 upstream_2**	TCATAGGTCAGGGCCCAGAA	CAGTGCGGGGCTTCAGTTAC
**LGR5**	AGGTCTGGTGTGTTGCTGAG	GAGTGACGTGGGGAAGTACT
**MYC control**	AAAAACGGGGTCAGAAGTCAGGAA	AGGTAAAGATTGGGGAAGCAGCAA
**16q22**	CTACTCACTTATCCATCCAGGCTAC	ATTTCACACACTCAGACATCACAG
**AltR mut Assay**		
**LARGE2 guide 1**	GATTCCTCCTGTTCGGTGGG	GCGTGGAAAAGCTCTTTGCA
**LARGE2 guide 2**	GGACATCTGCGGGAATCACA	GCGTGGAAAAGCTCTTTGCA
**APC exon7**	CGACCGCCAATCGTACTGGAGG	GACAGCACATTGGTACTGAATGC
**APC exon15**	TGTAATCAGACGACACAGGAAGCAGA	TGGACCCTCTGAACTGCAGCAT
**cRNA sequences for APC targeting**		
**guide 1 (Exon 7)**	GGCAACTTCTGGTAATGGTC	IDT technologies
**guide 2 (Exon 15, MCR)**	GTACATCTGCTAAACATGAG	IDT technologies
**Primers for gateway cloning**		
**CTNNB1-S33Y**	aaaaagcaggcttcaccATGGCTACTCAAGCTGATTTGATG	agaaagctgggtgTTACAGGTCAGTATCAAACCAGGC
**attB - universal primers**	GGGGACAATTTGTACAAAAAAGCAGGCTTCAC	GGGGACCACTTTGTACAAGAAAGCTGGGTG
**Large2 in situ probe primer**		
**Large2 probe**	CTCTATGGACCGGCTGCAGATGCTG	GAGAATTAGACTAGCCCCAAACCAGC
**TCF7L2 fragment for pBV-Luc**		
**TCF7L2**	GCCGAATTCGCACAATCACGACGATCTG	GGGCTCAGGTTTCACCTCAAAGCTTCCG
**TCF7L2 mut**	GTGTCGAGCTCCCGCAGTGTCTTTGGCCCCGGGCCCGGGGACACTGG	GAGGGAGGAGGAGGGGGCCGGAATGCAGG

**Plasmids**	**Description**	**Distributor**
**LARGE2 ORF**	In pENTR221 Gateway full ORF clone	GPCF, DKFZ Heidelberg
**pMD2.G**	2nd generation envelope vector for virus production	A. Trumpp, DKFZ Heidelberg
**psPAX2**	2nd generation packaging vector for virus production	A. Trumpp, DKFZ Heidelberg
**pSpCas9(BB)-2A-GFP**	Cas9 from S. pyogenes with 2A-EGFP, and cloning backbone for sgRNA	Addgene plasmid #48138
**pLenACRISPR-E**	Introduces sgRNA into a lentiviral vector (LentiCRISPR V2) which contains eSpCas9 and puromycin cassette	Addgene plasmid #78852
**pTRIPZ non silencing**	Glycerol stock, negative TRIPZ Inducible Lentiviral shRNA Control	Dharmacon, RHS4743
**pTRIPZ APC shRNA 1-3**	Glycerol set, TRIPZ Inducible Lentiviral Human APC shRNA 1-3	Dharmacon, RHS5087
**pBV-Luc**	luciferase reporter plasmid with very low basal activity	Addgene plasmid #16539
**pV2luc2**	Encodes for Luciferase and Venus fluorescent protein	A. Trumpp, DKFZ Heidelberg
**FUW-NE and FUW-ERT2**	Encode for CTNNB1-binding domain of TCF7L2 fused to hormone-binding domain of estrogen receptor (ERT2) (NE) or the ERT2 domain alone	Eduard Batlle, IRB Barcelona
