# Supplementary material for: Wnt-driven LARGE2 mediates laminin-adhesive O-glycosylation in human colonic epithelial cells and colorectal cancer
Source: Cell Commun Signal. 2020 Jun 25;18:102. doi: 10.1186/s12964-020-00561-6 (PMC7315491; doi:10.1186/s12964-020-00561-6)

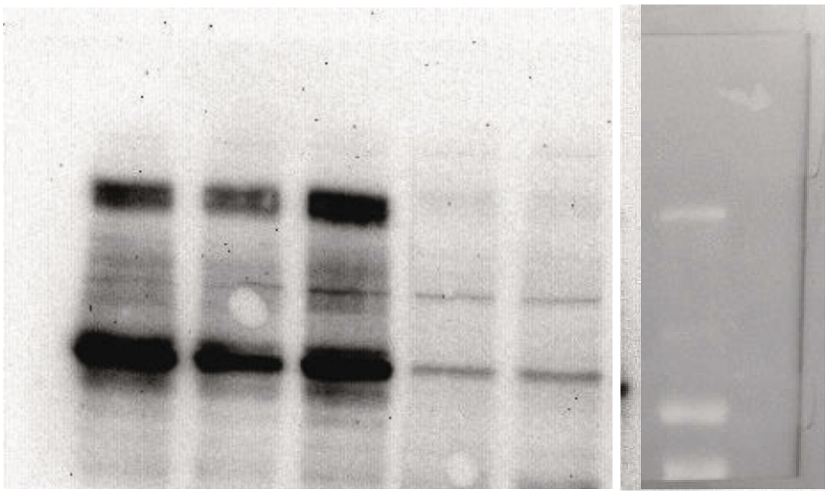

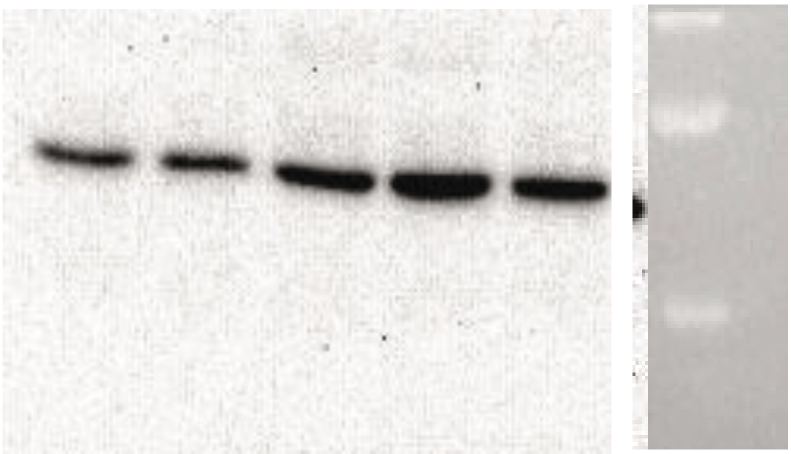

Figure 3B

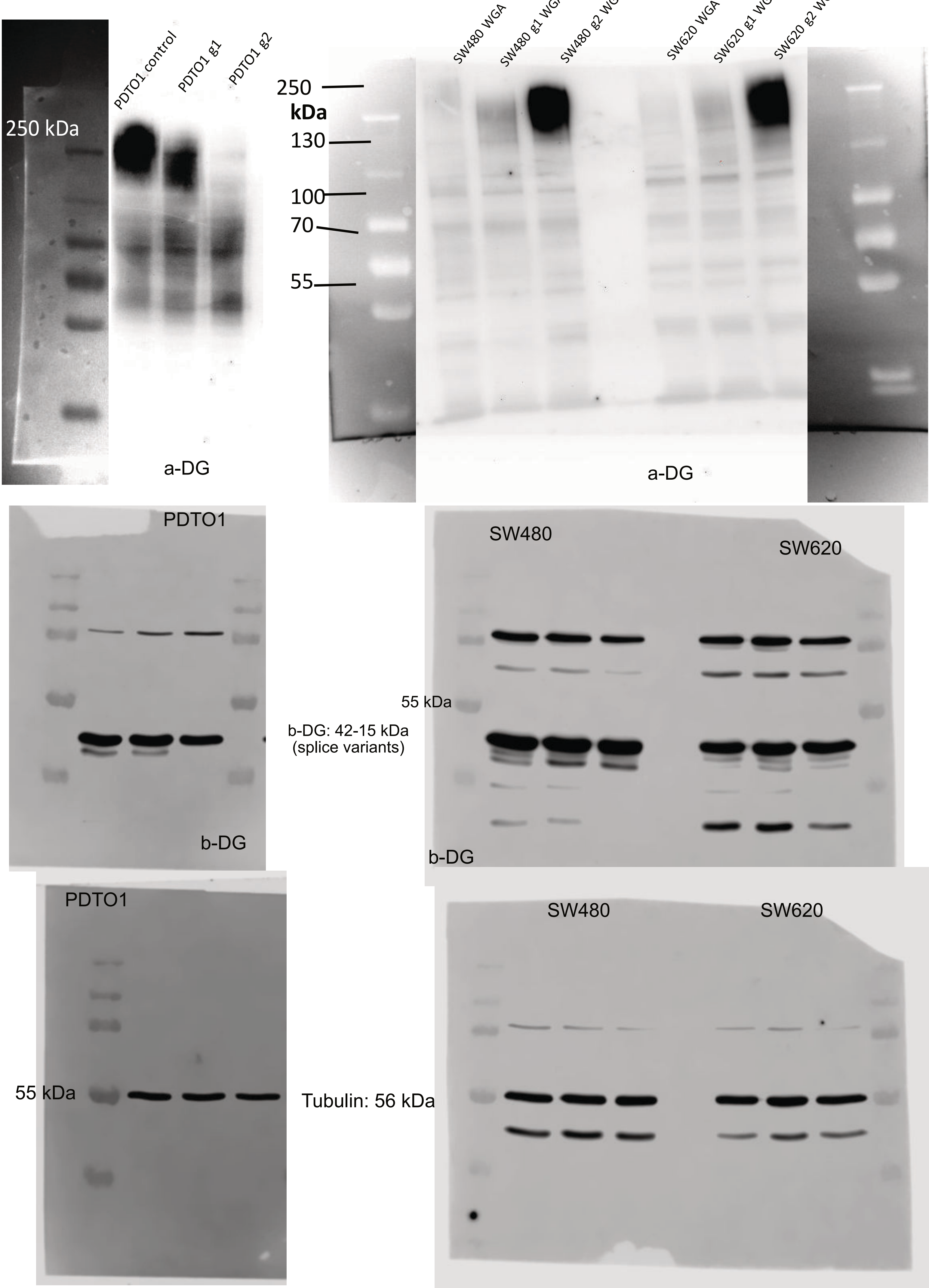

PDTO1 control
PDTO1 g1
PDTO1 g2
250 kDa
a-DG
SW480 WGA
SW480 g1 WGA
SW480 g2 WGA
SW620 WGA
SW620 g1 WGA
SW620 g2 WGA
250
kDa
130
100
70
55
a-DG
PDTO1
b-DG
SW480
SW620
55 kDa
b-DG: 42-15 kDa
(splice variants)
b-DG
PDTO1
55 kDa
Tubulin: 56 kDa
SW480
SW620

Figure 3C

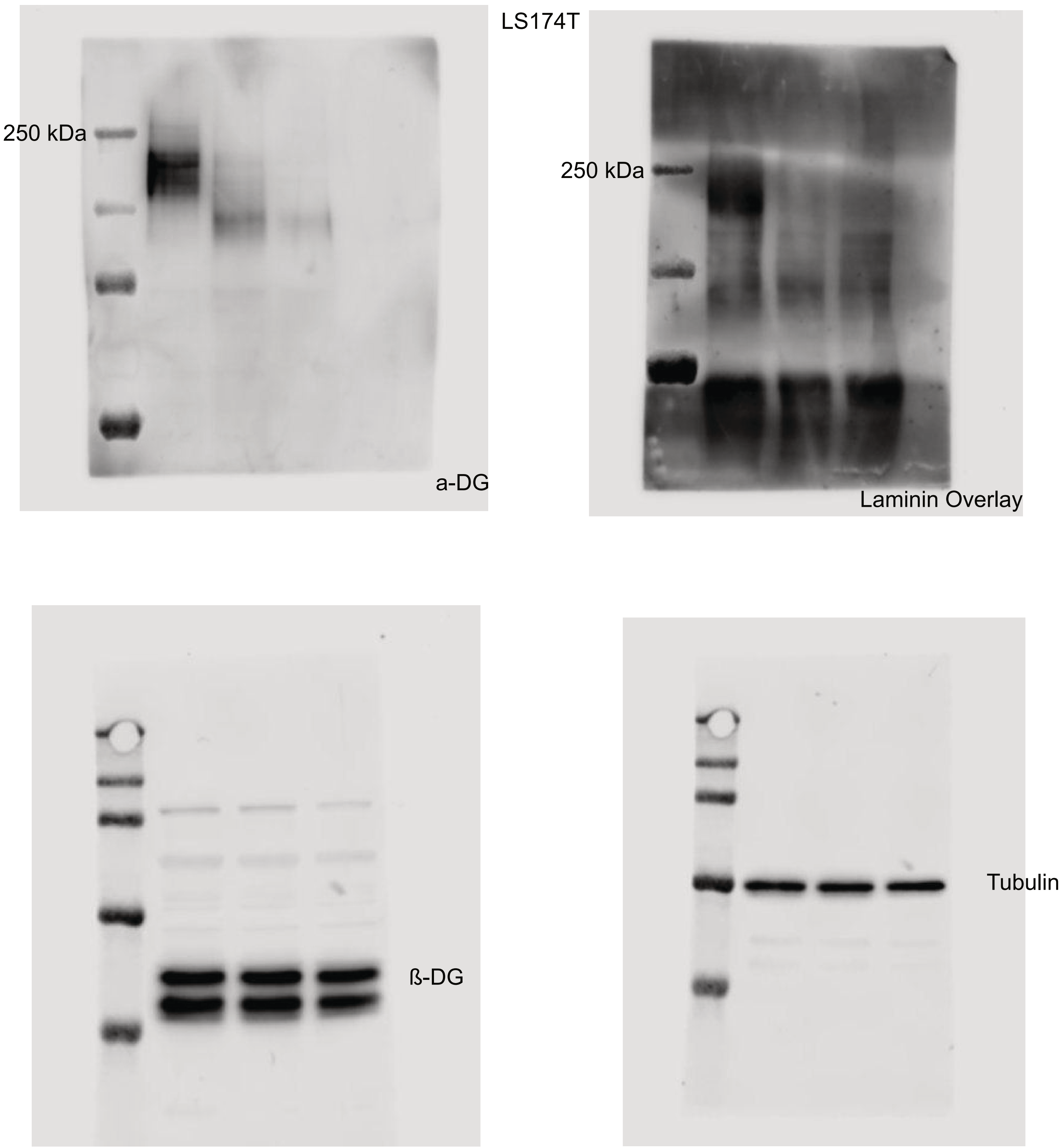

Figure 3E

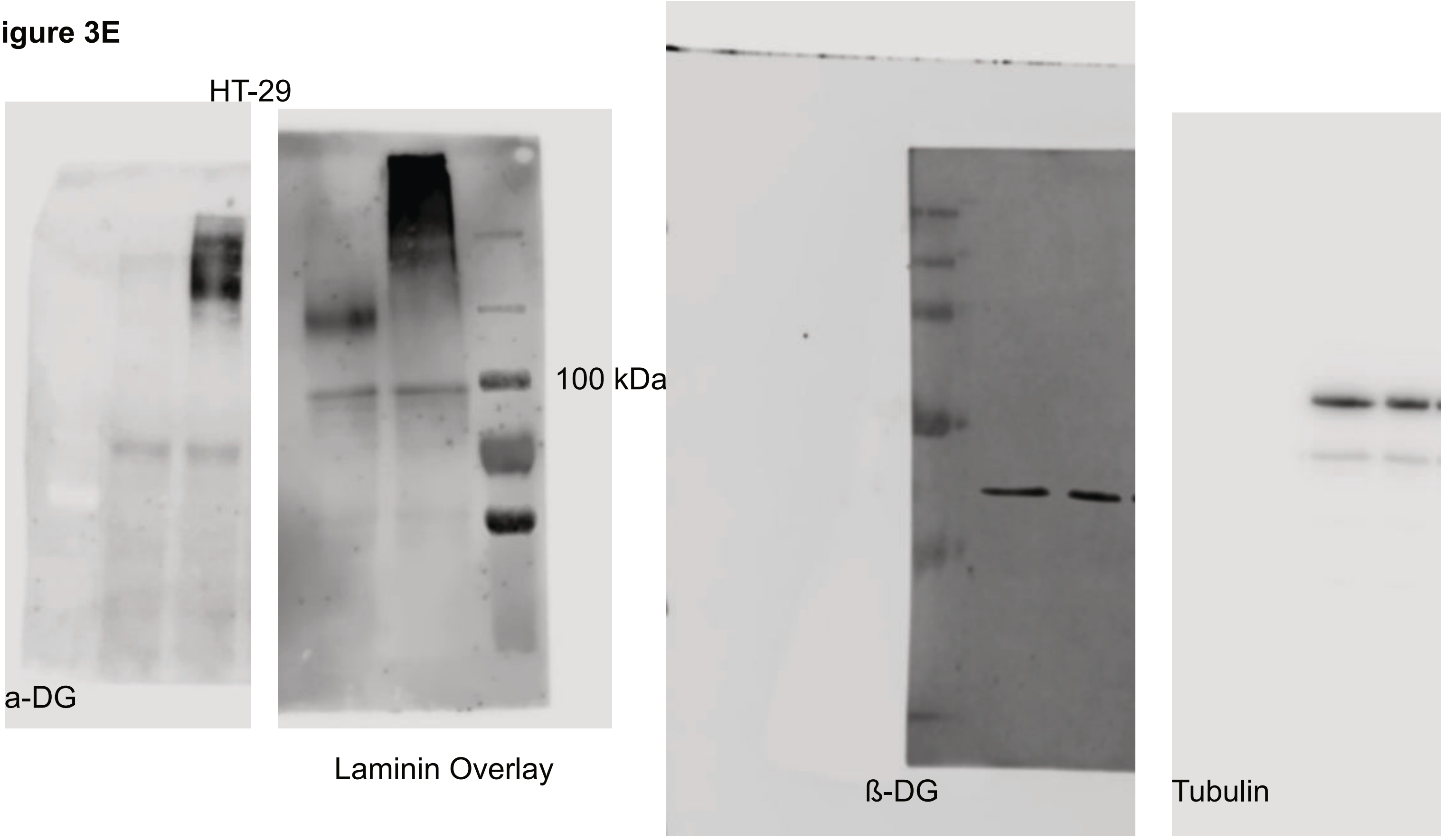

Figure 4A

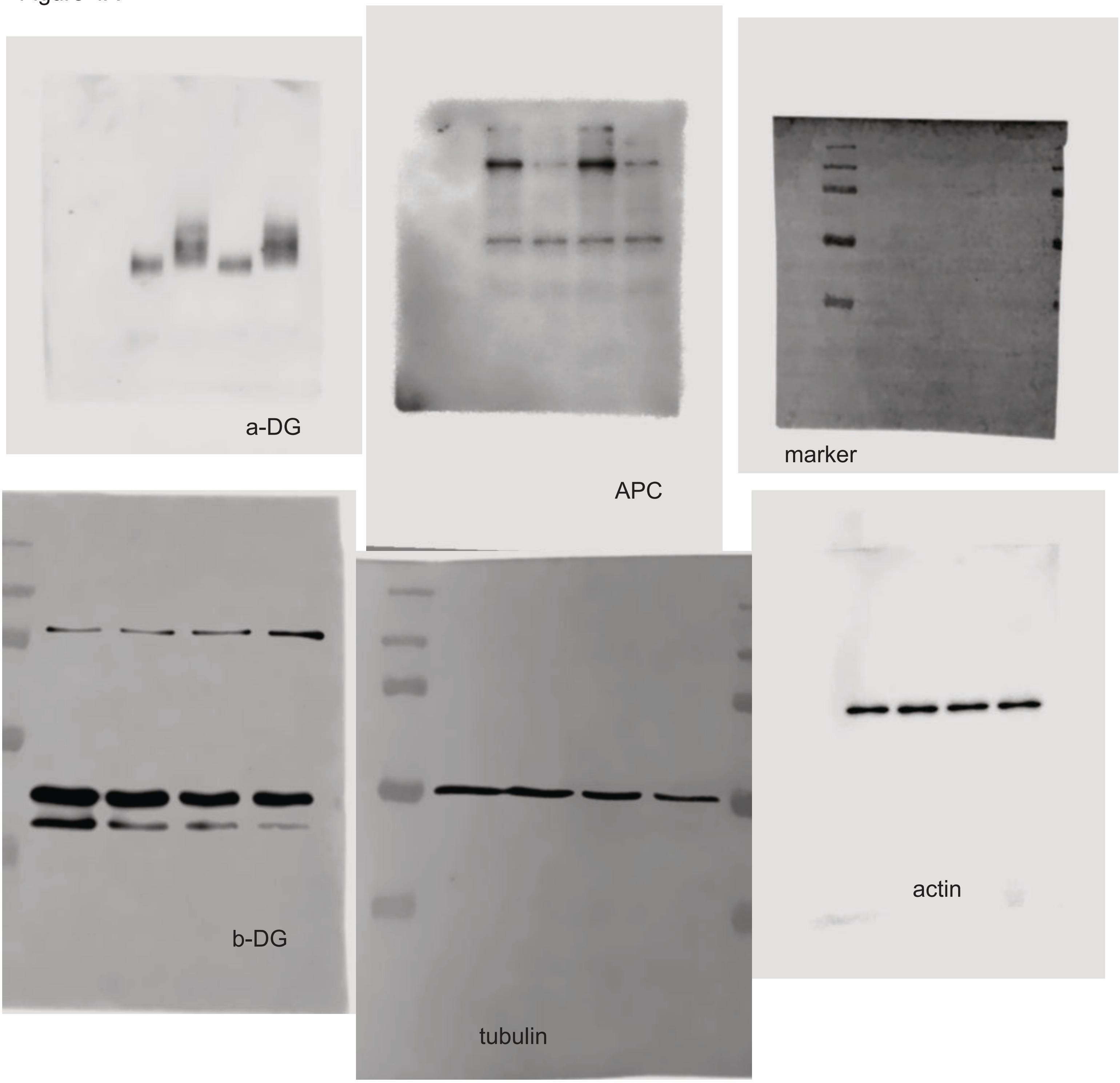

Figure 4B

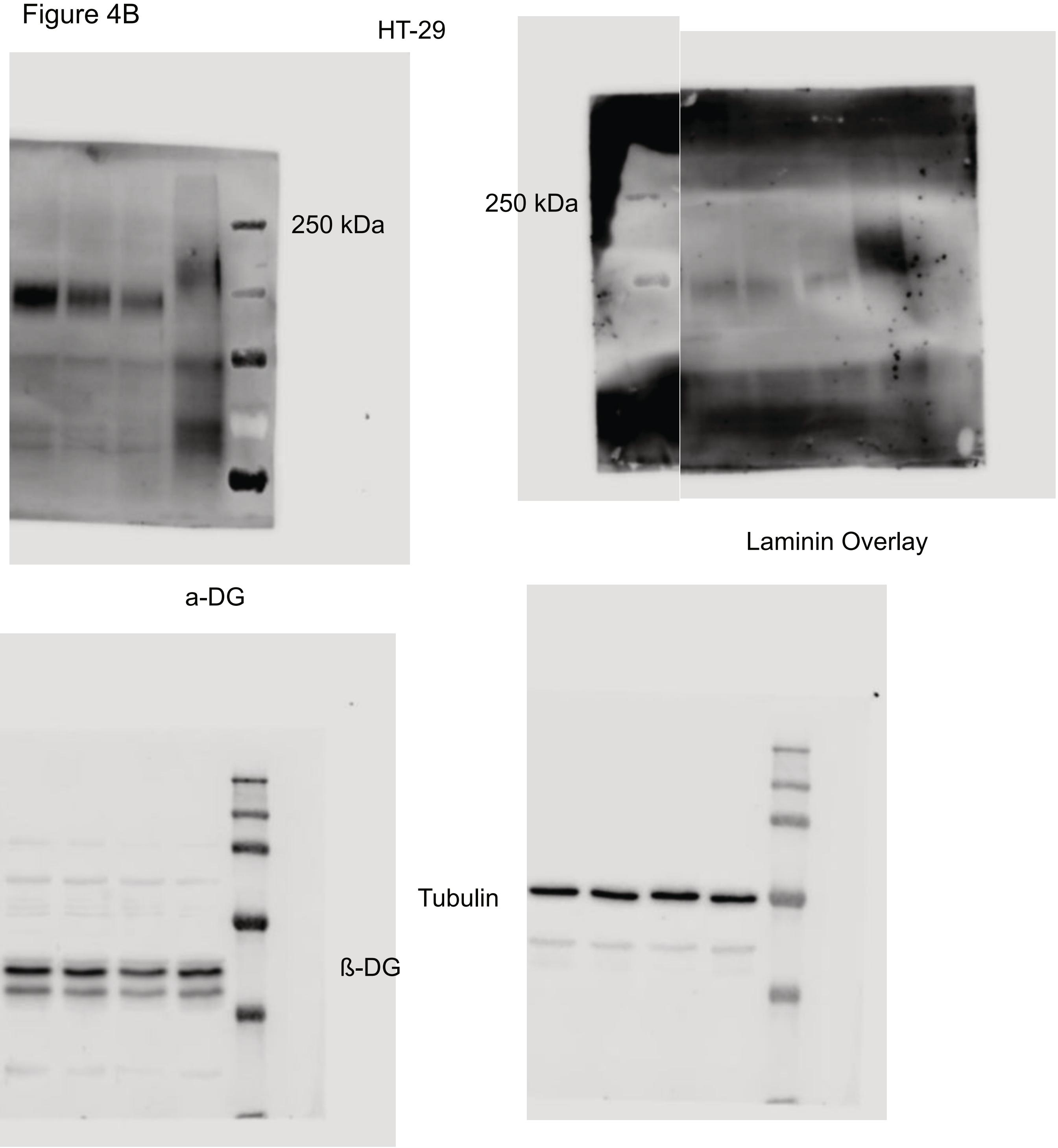

Figure 4C

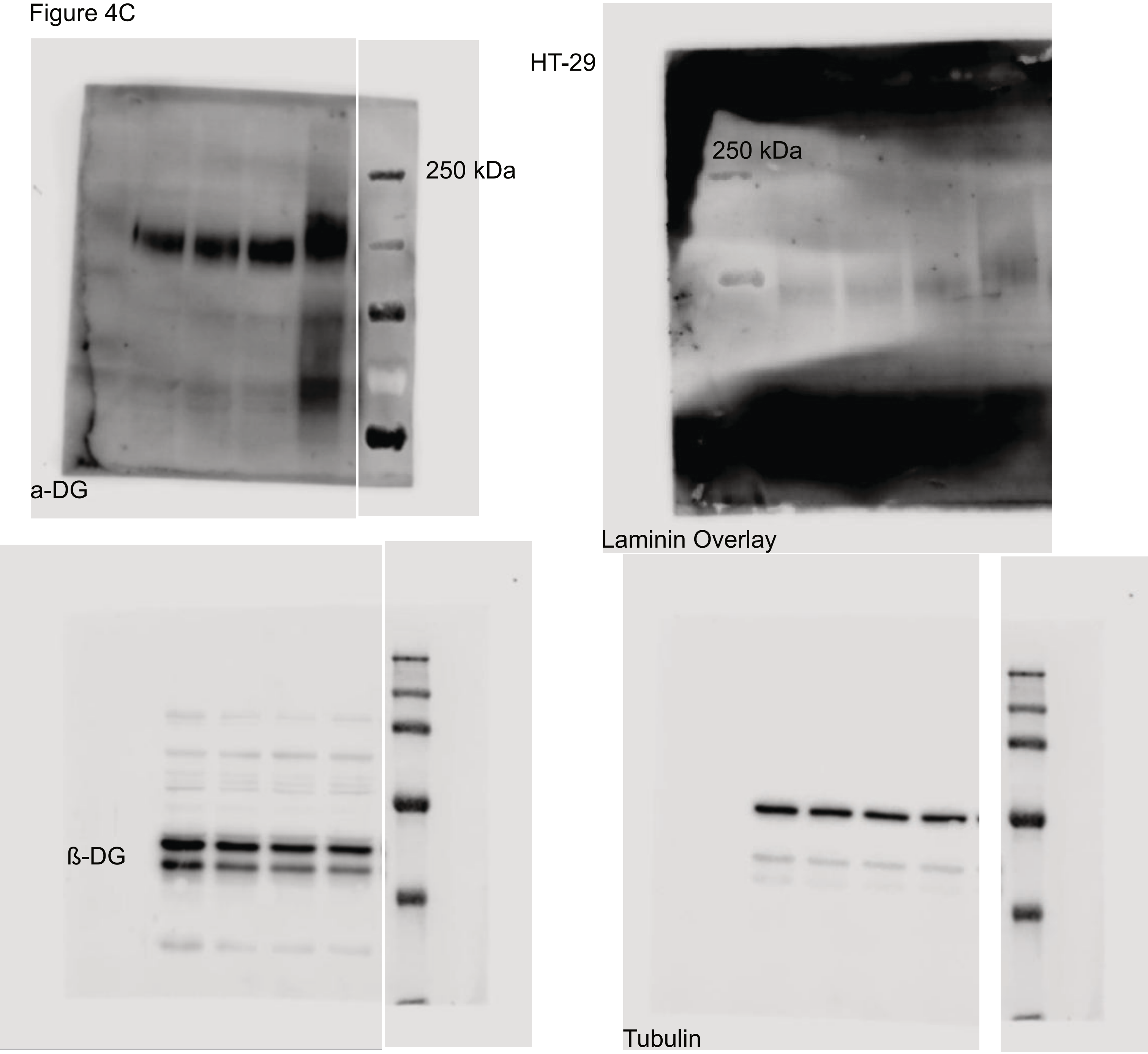

Figure 4E

HT-29 TCF7L2BS

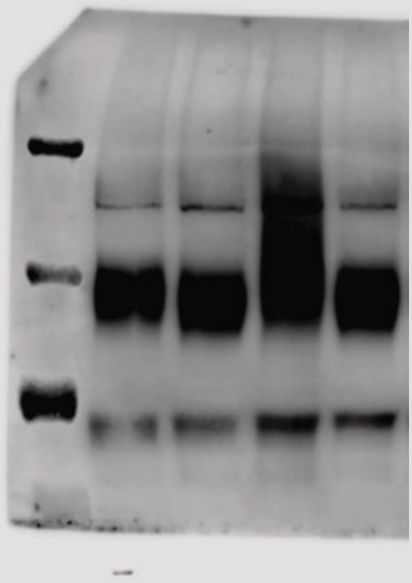

aDG

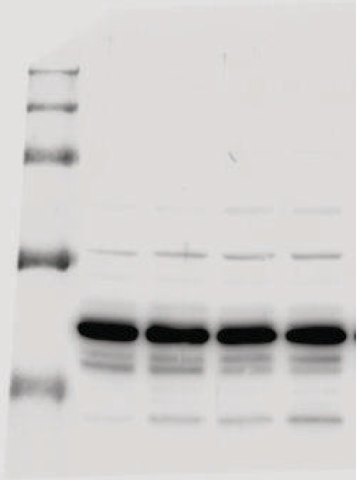

bDG

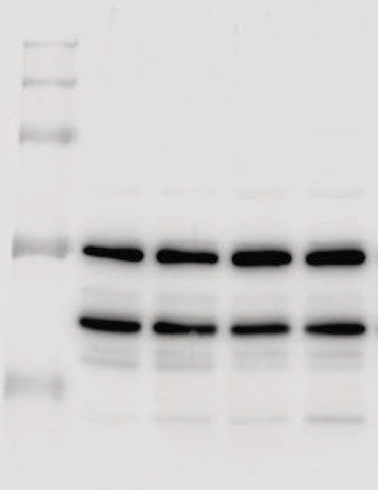

Tubulin

Figure 4F

LS174T

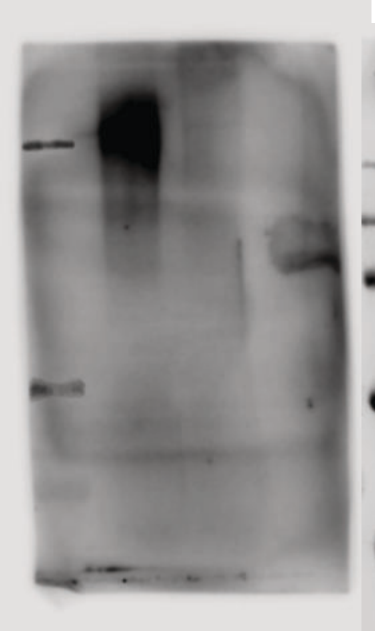

aDG

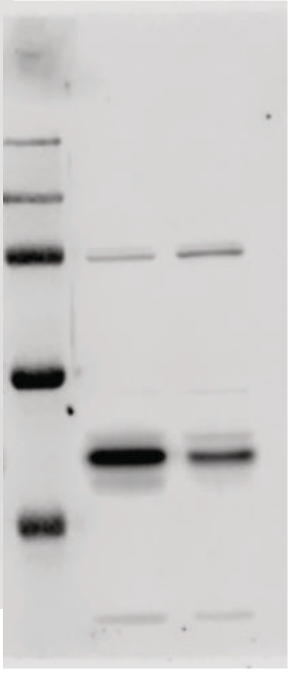

b-DG

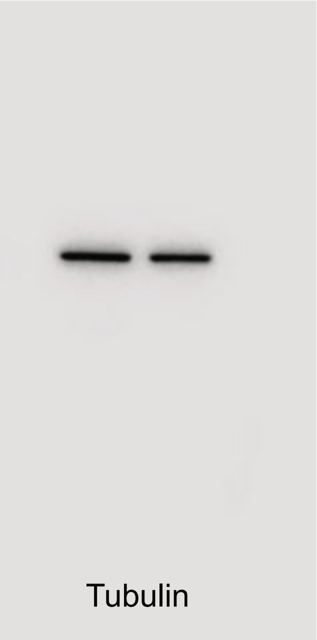

Figure 4G

SW480 ERT - NE

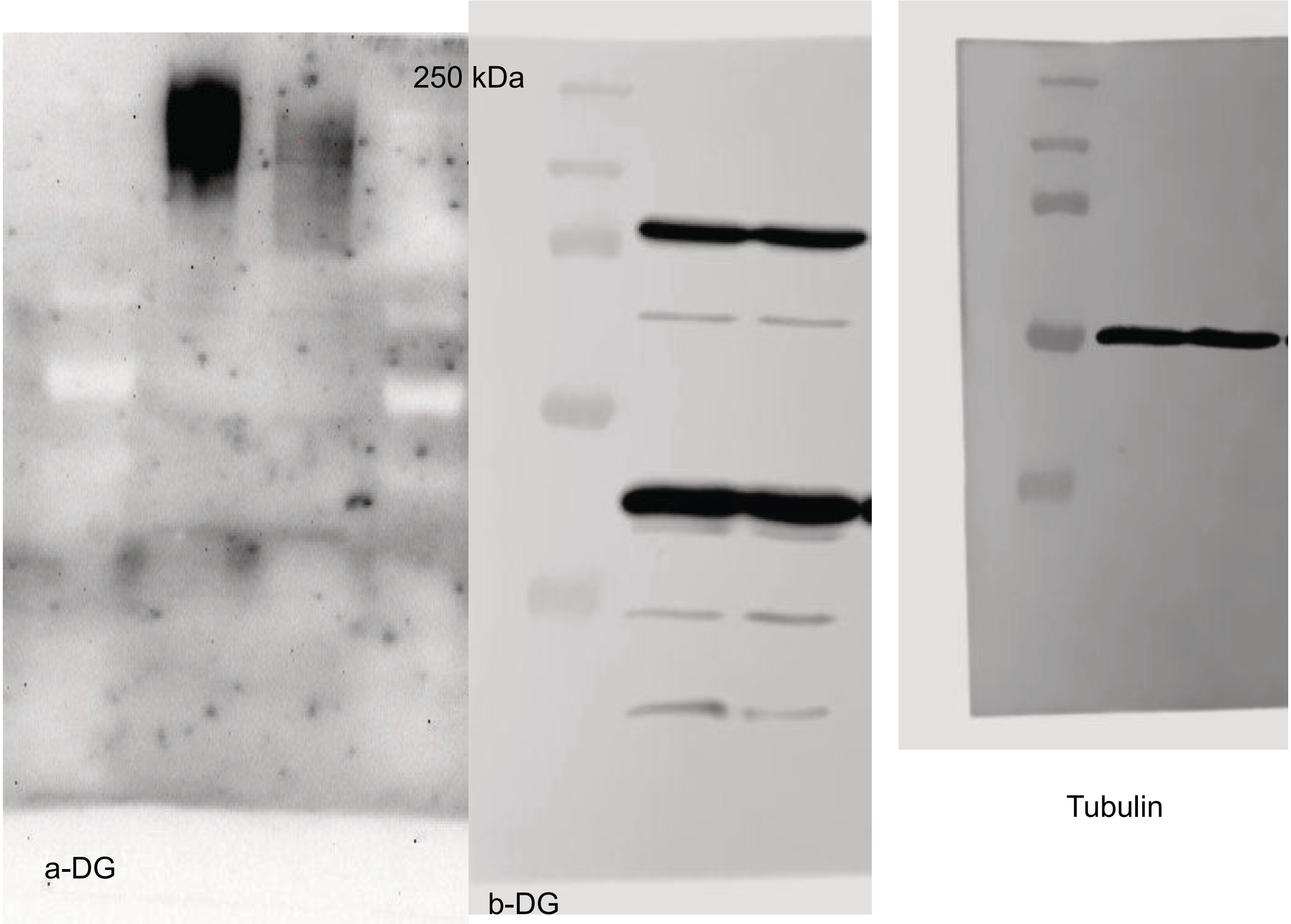

Figure 4H

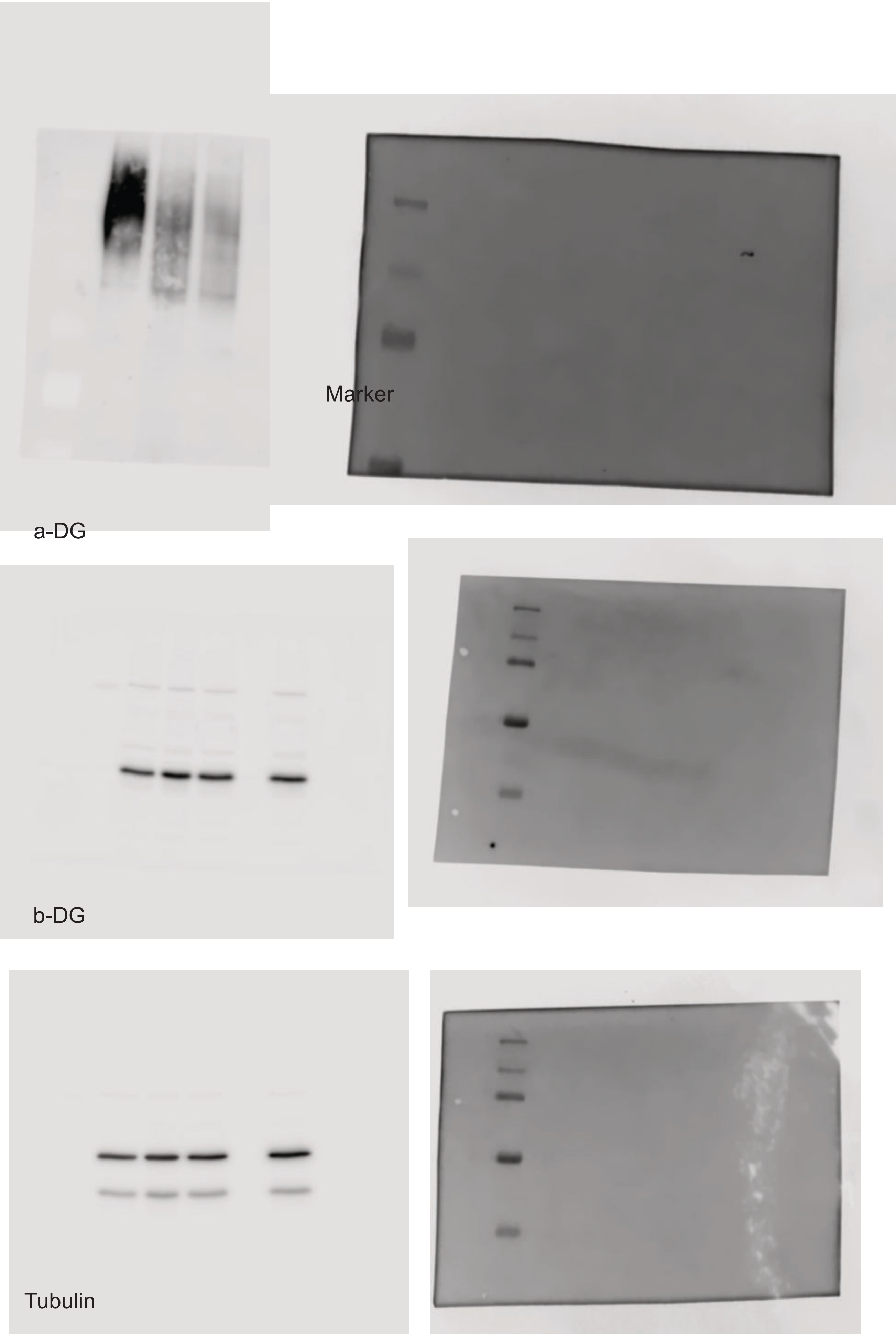

Figure 4I

PDTO1 TCF7L2BS

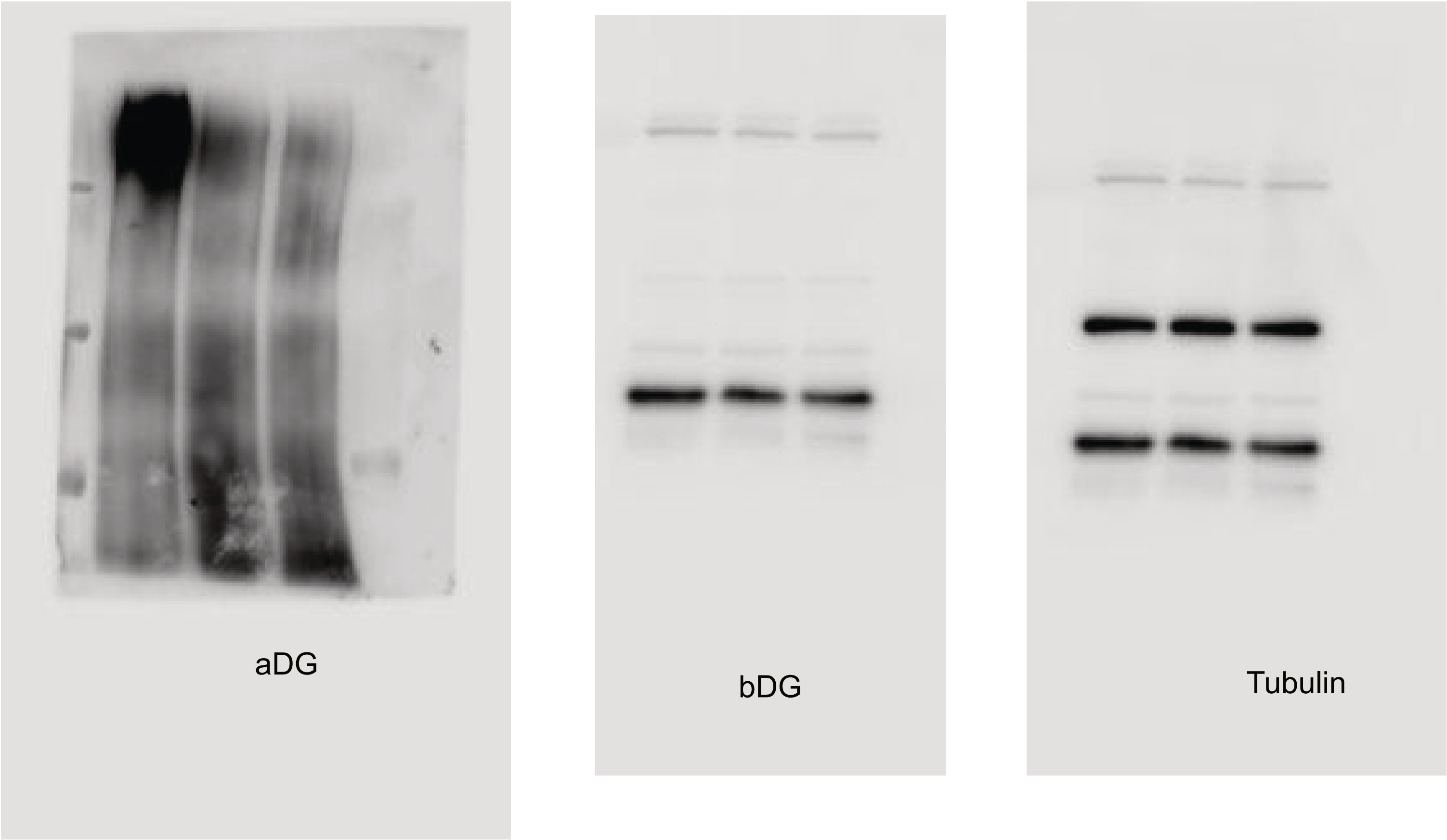

Figure 5C

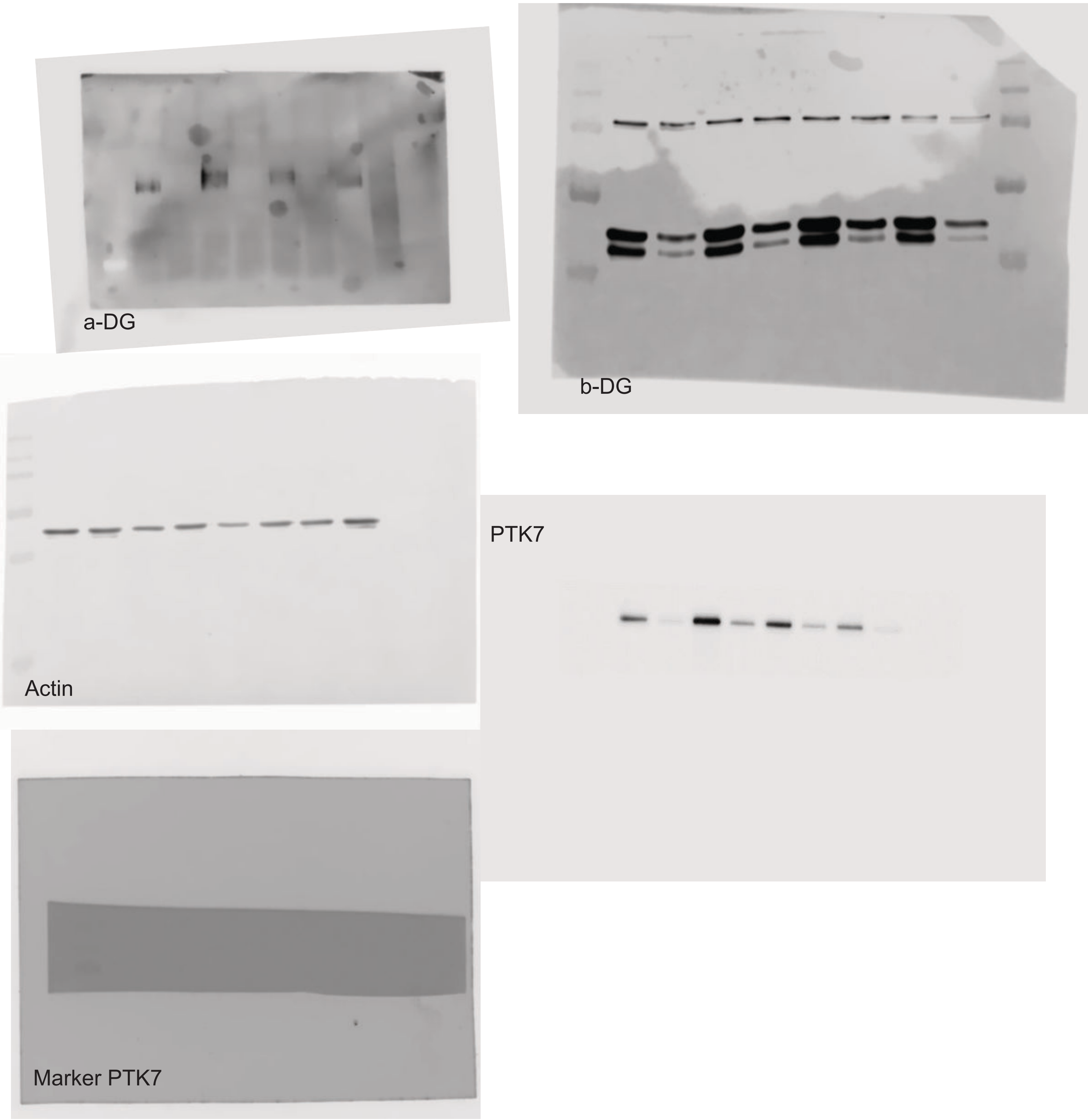

Figure 6J

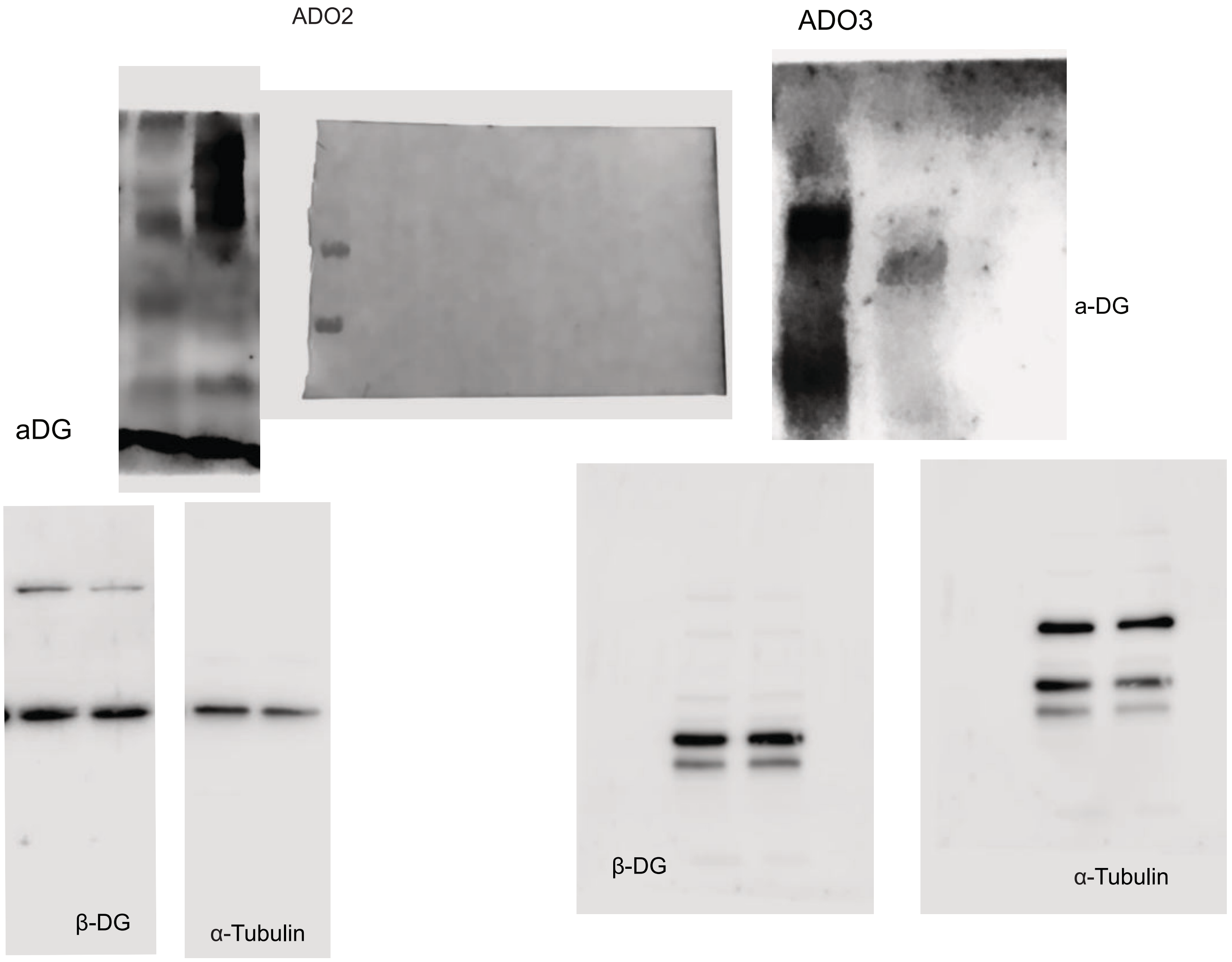

Figure 7D

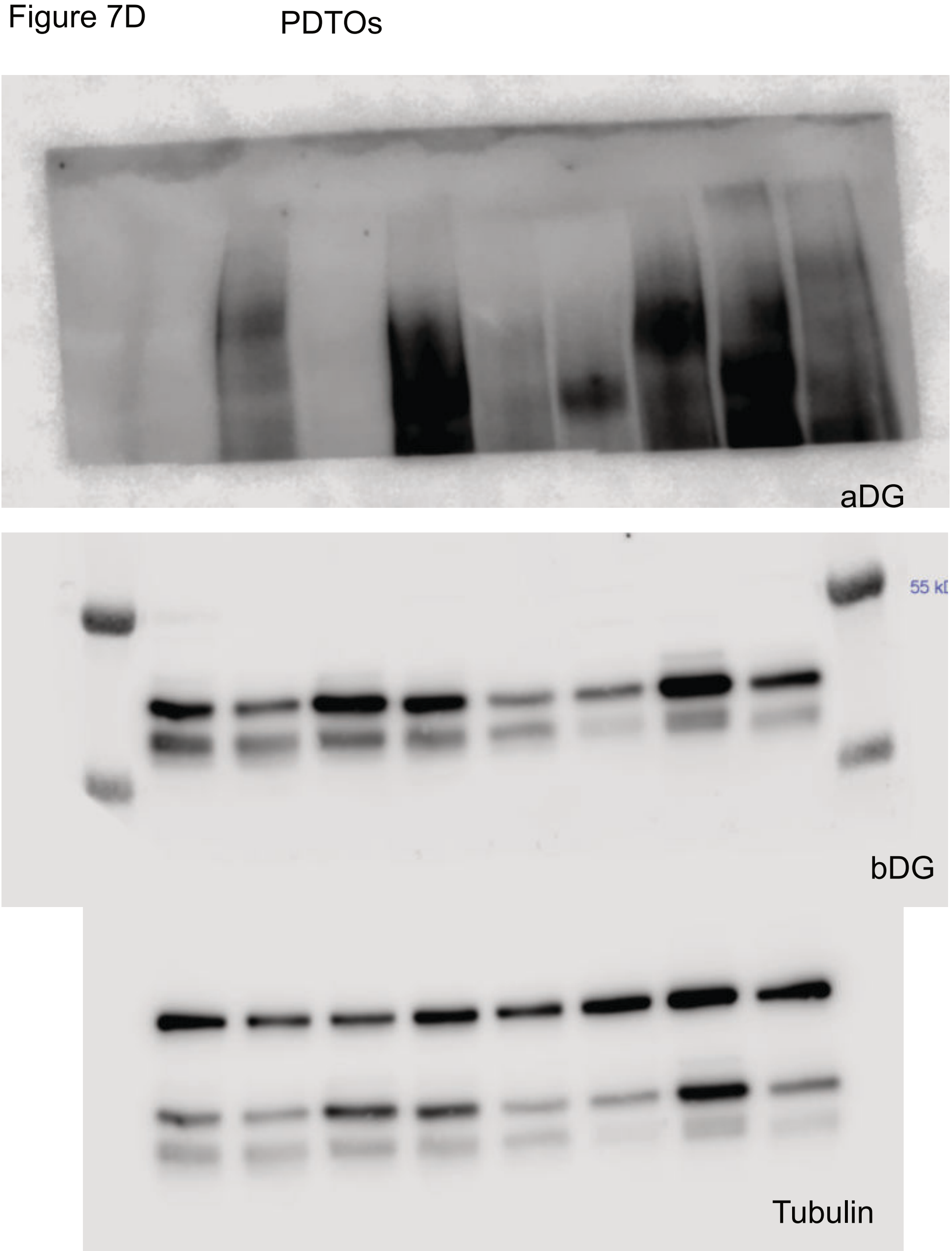

Figure 7F

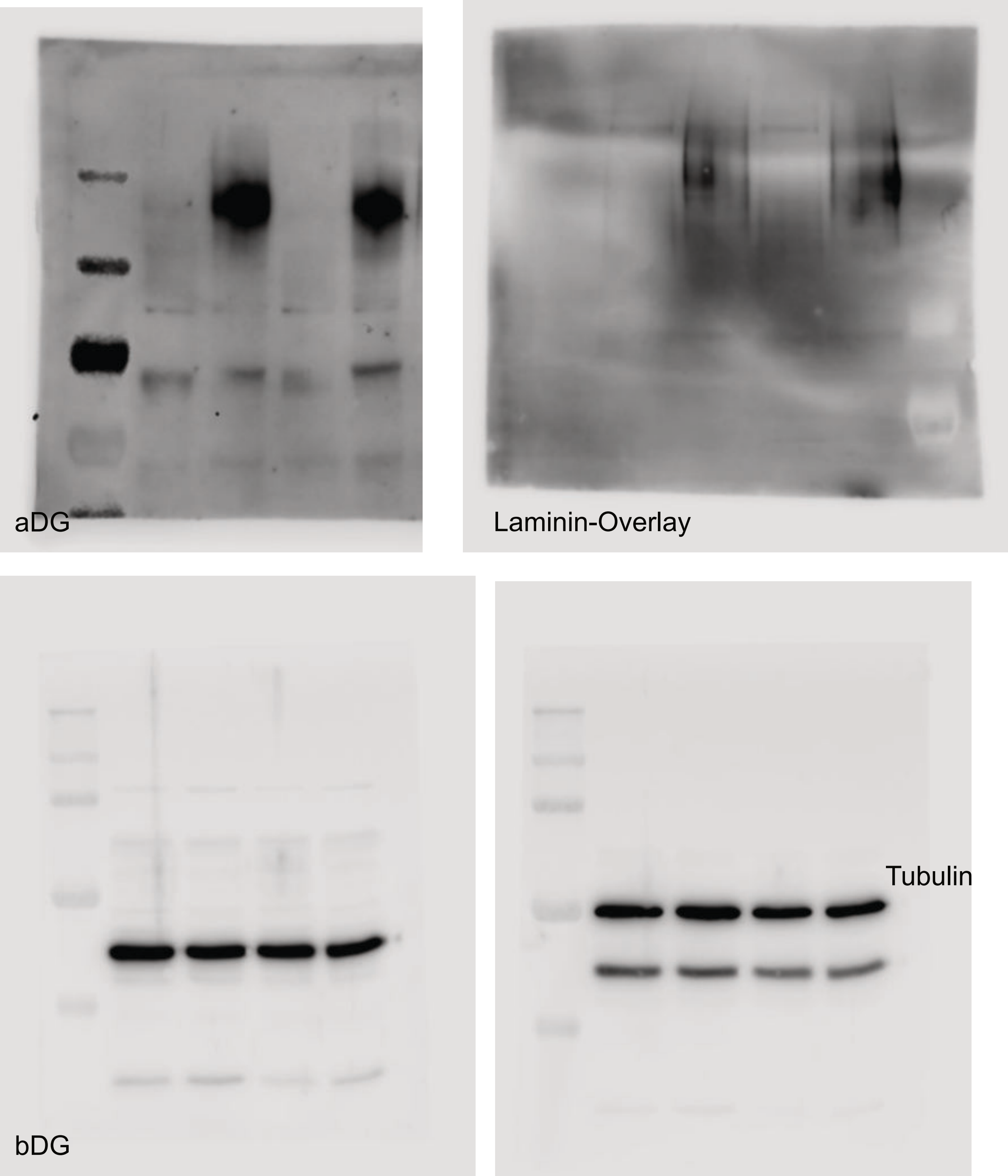

Figure 7G

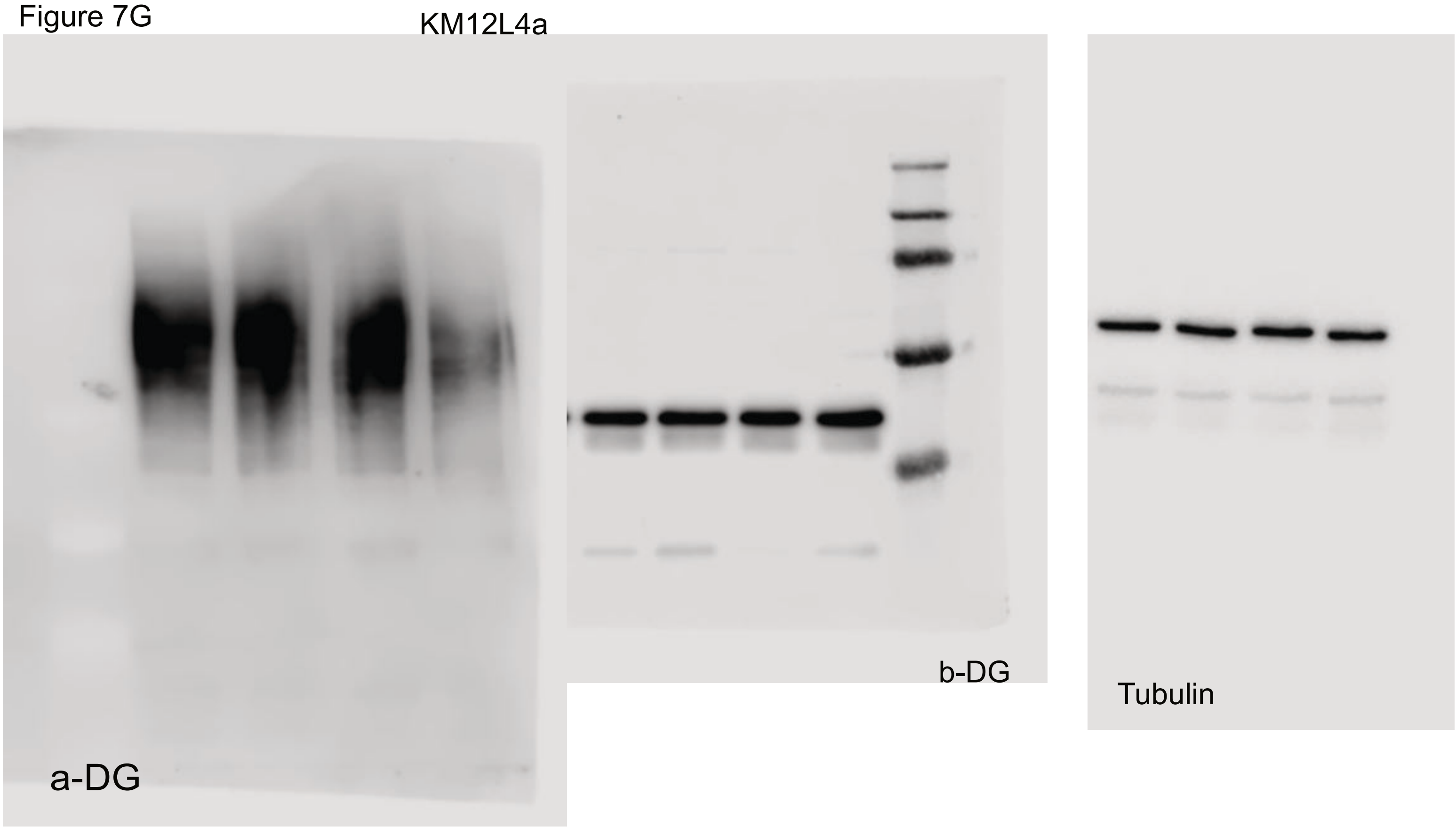

Additional File 2B

Colo 205 sh APC

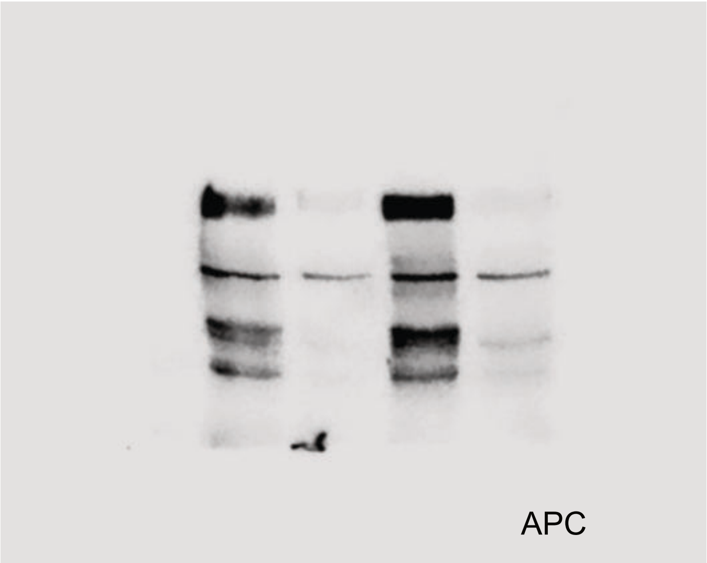

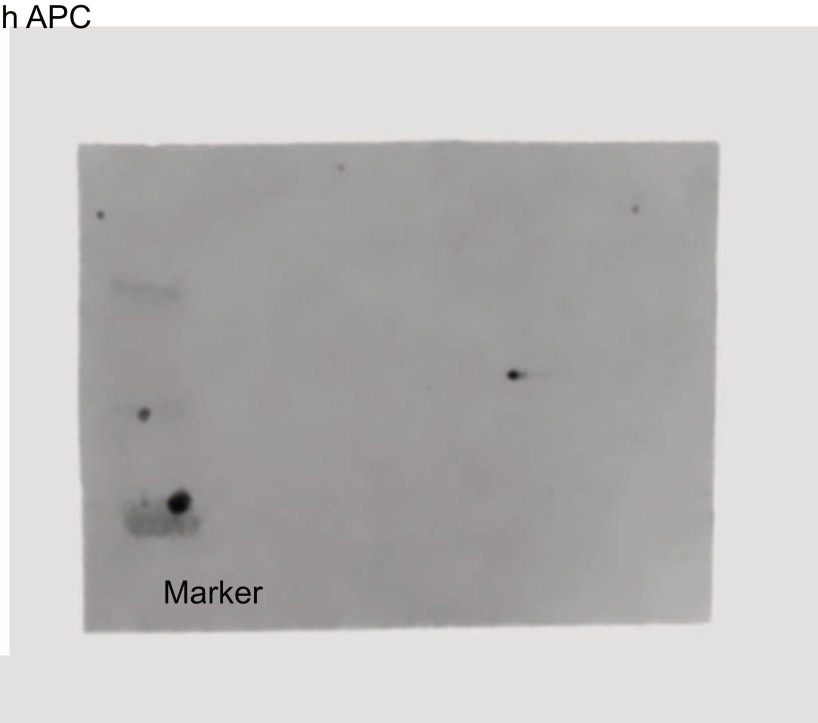

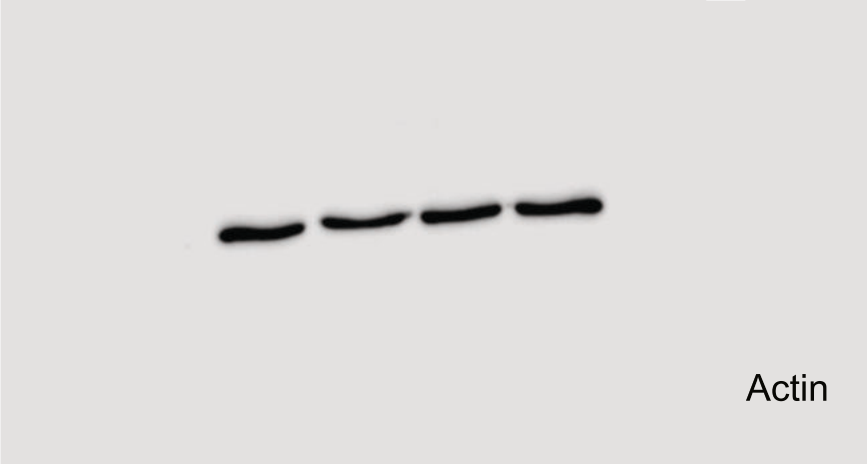

Additional file 6A SW620 pSpCas9 single clones

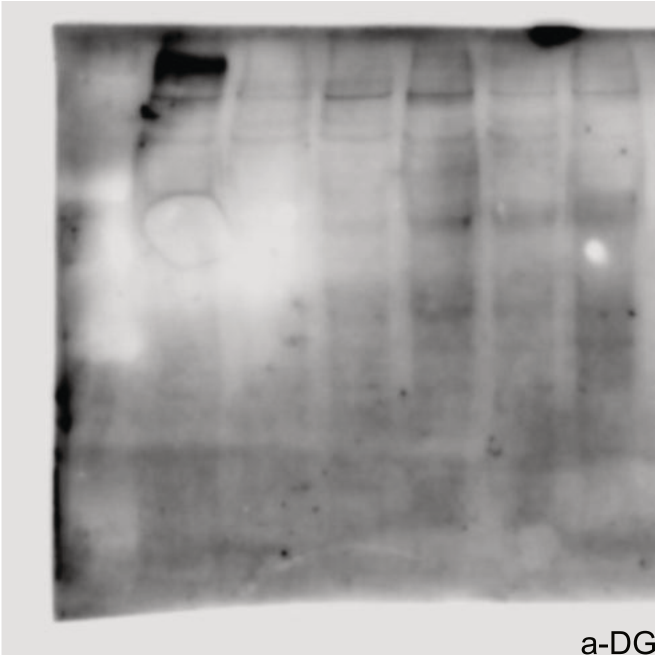

a-DG

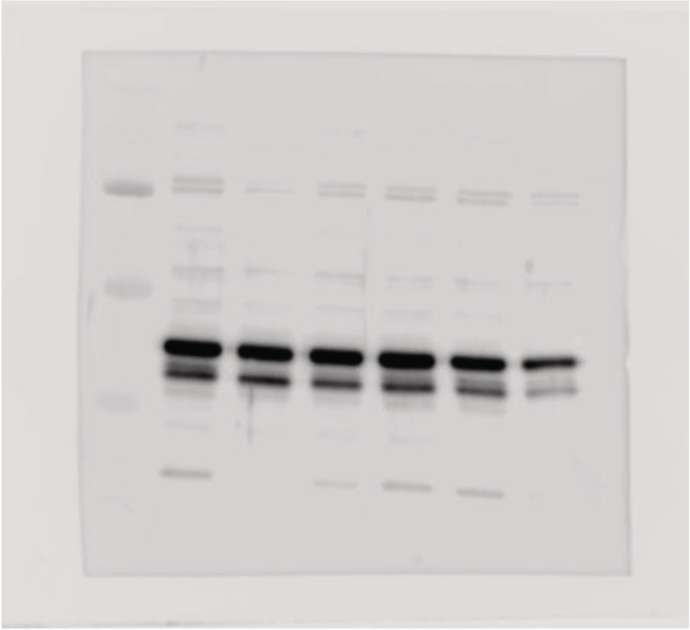

ß-DG

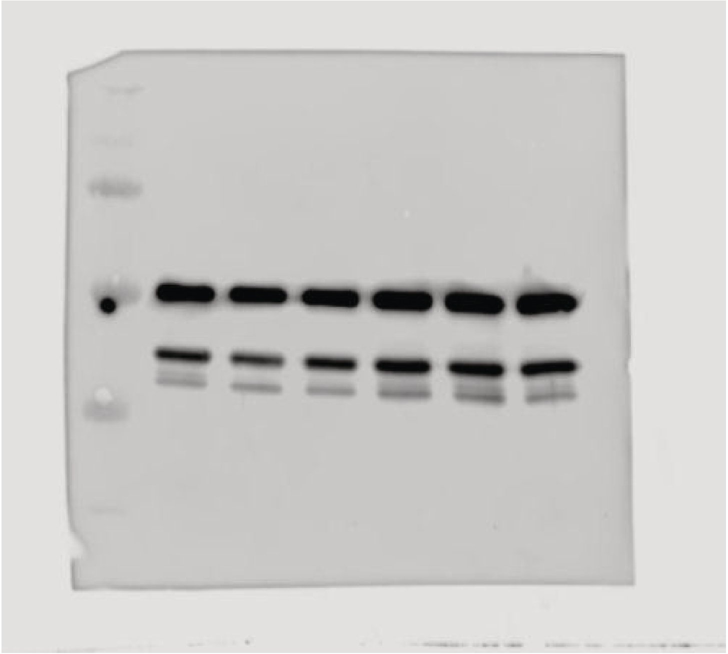

Tubulin

Additional file 8C

PDO3

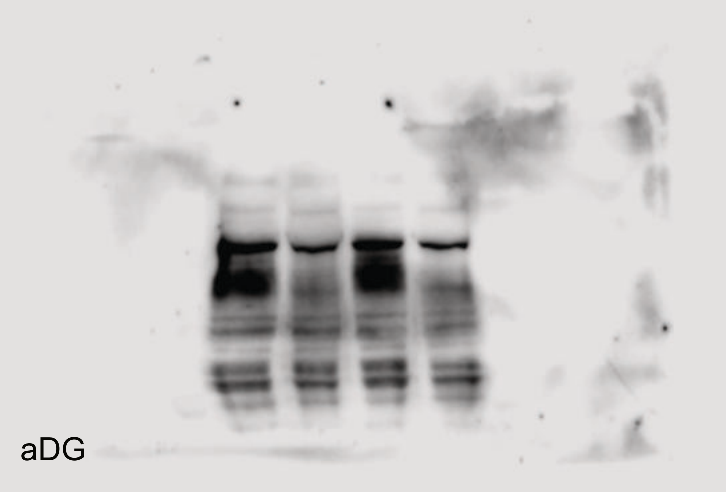

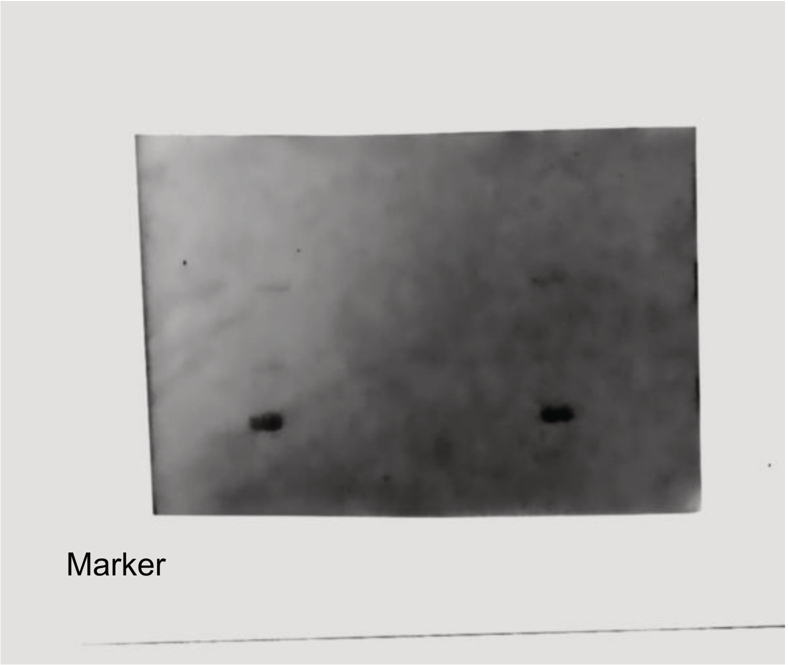

PTK7

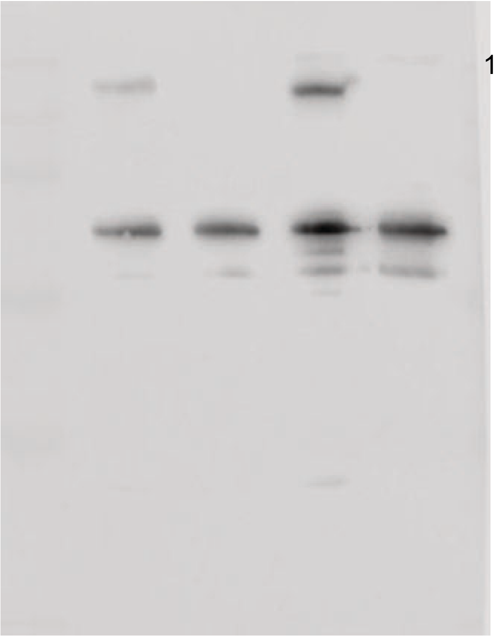

160 kda

β-Actin

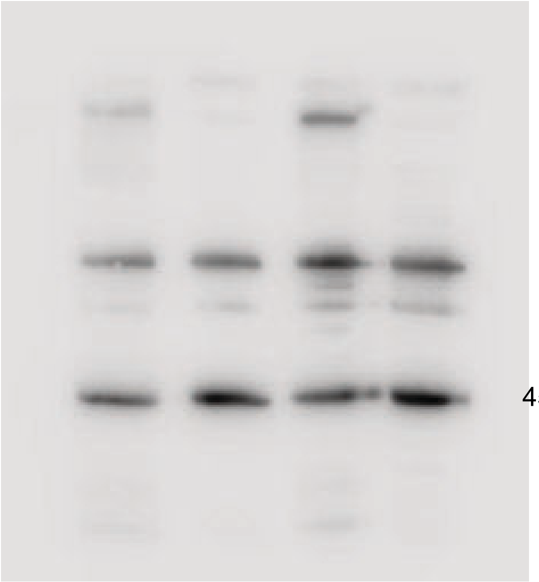

45 kda

β-DG

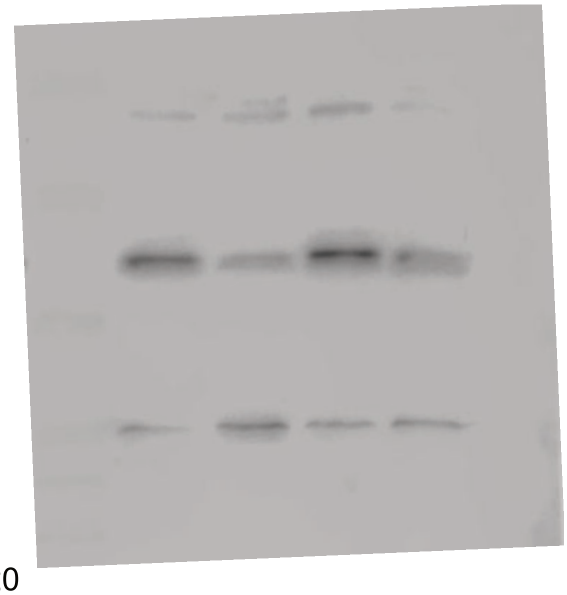

α-Tubulin

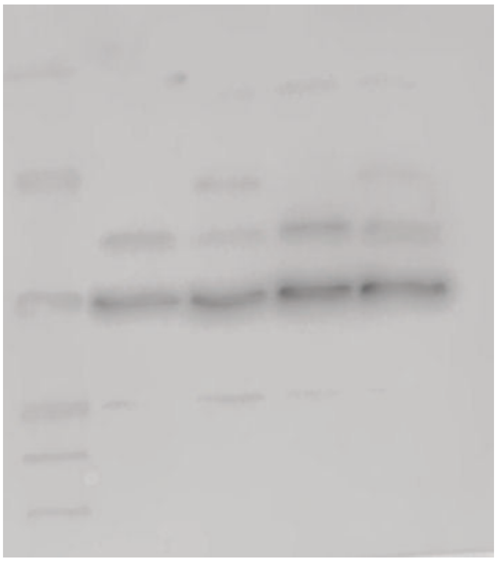

Additional file 10 E

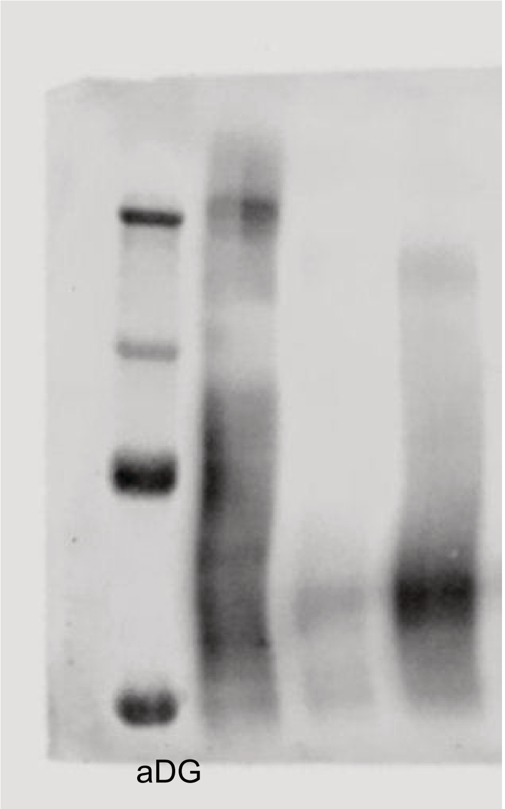

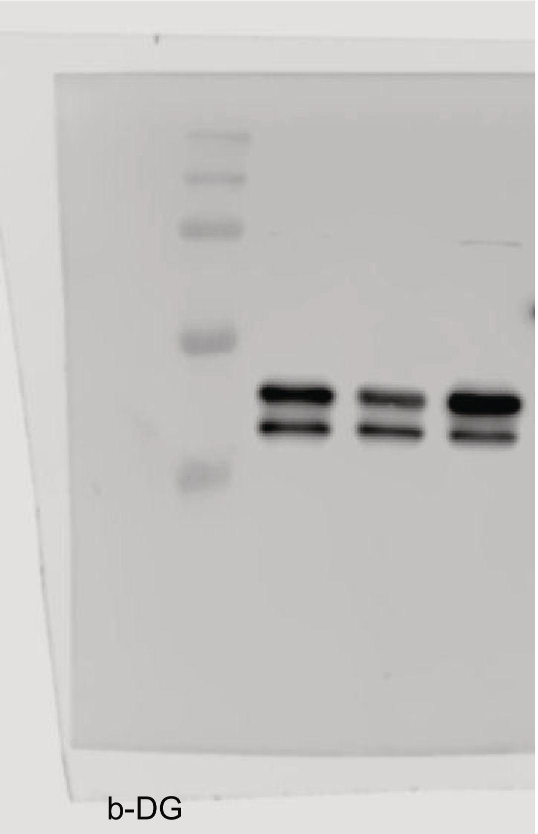

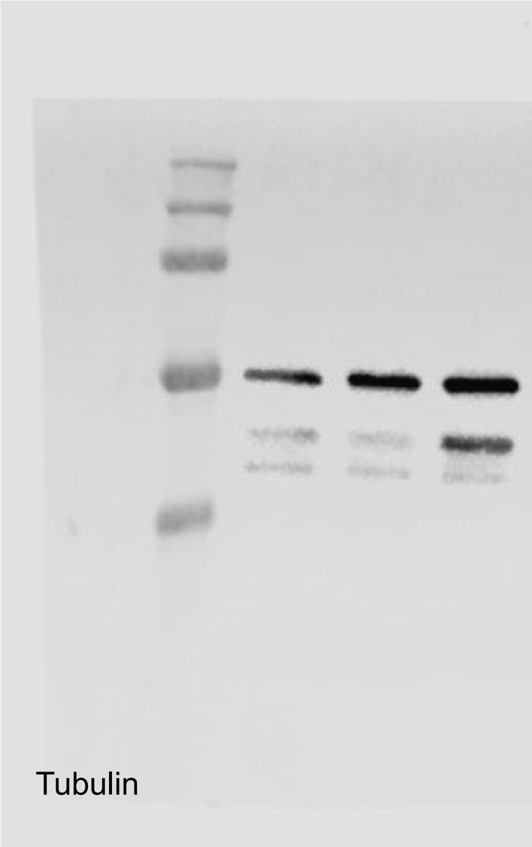

Additional file 10 F

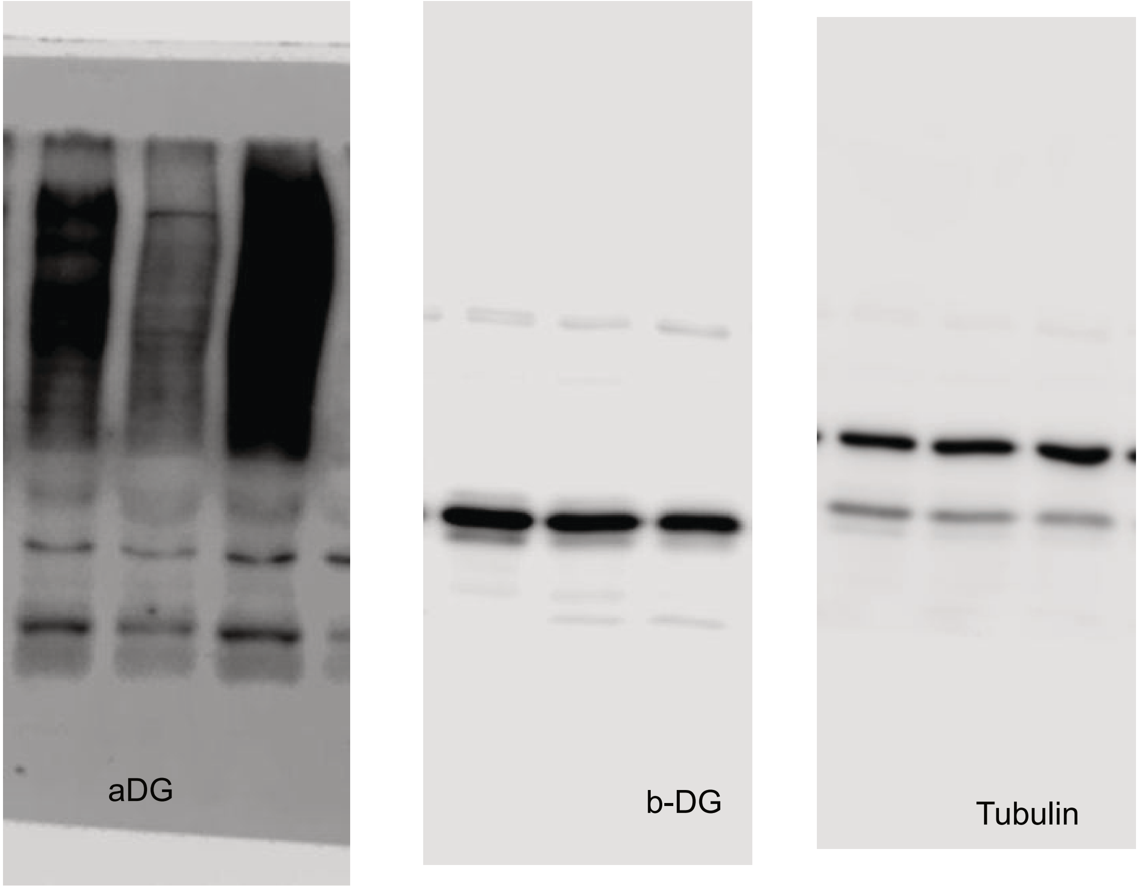

Additional file 10 H

KM12L4a KO

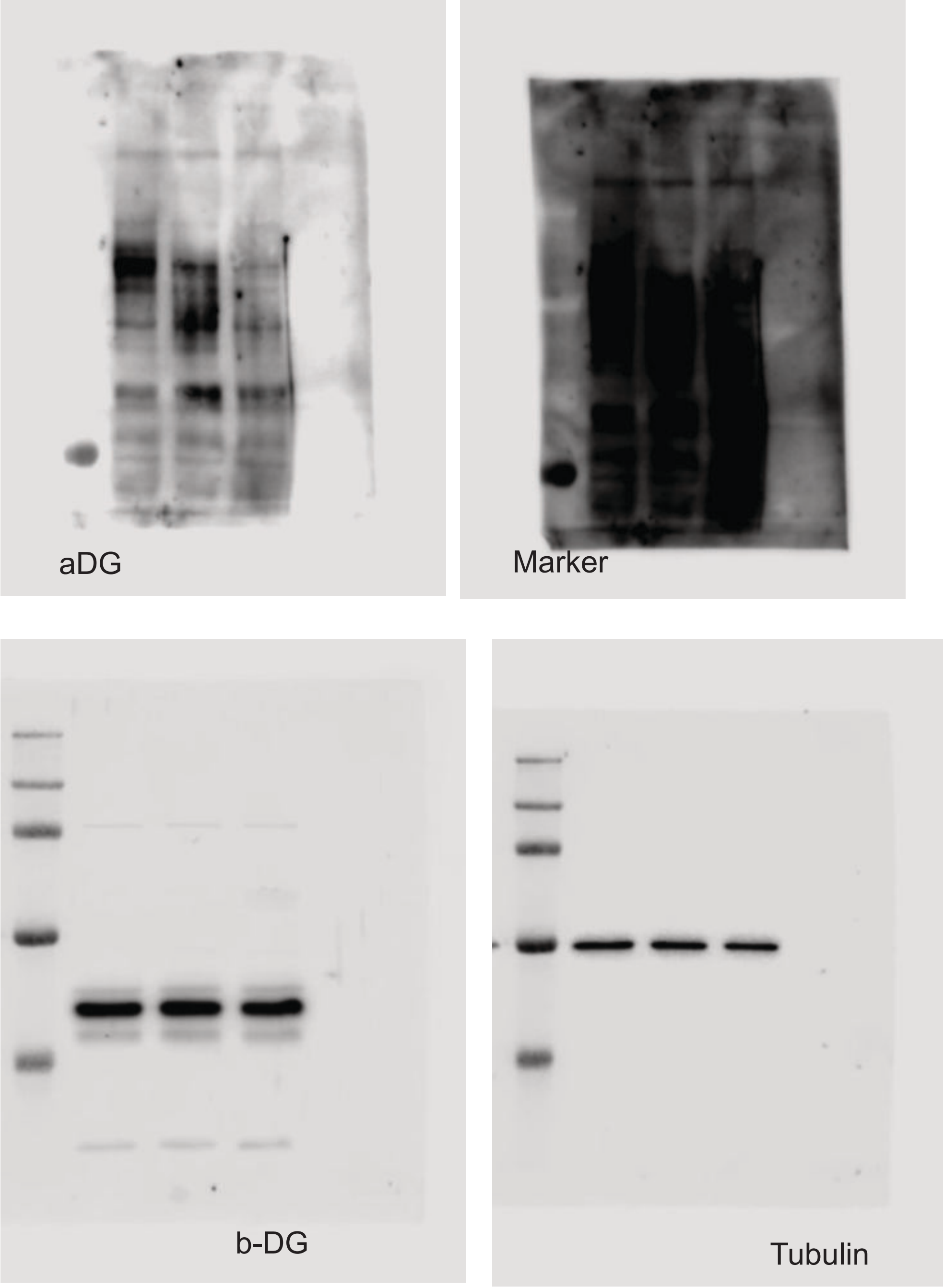

Supplement: Supplementary file 15 — Additional file 14. Uncropped images of immunoblot membranes. [file 12964_2020_561_MOESM14_ESM.pdf]
